# Surface Model and Tomographic Archive of Fossil Primate and Other Mammal Holotype and Paratype Specimens of the Ditsong National Museum of Natural History, Pretoria, South Africa

**DOI:** 10.1371/journal.pone.0139800

**Published:** 2015-10-06

**Authors:** Justin W. Adams, Angela Olah, Matthew R. McCurry, Stephany Potze

**Affiliations:** 1 Department of Anatomy and Developmental Biology, Faculty of Medicine, Nursing and Health Sciences, Monash University, Clayton, Victoria, Australia; 2 Department of Biological Sciences, Faculty of Sciences, Monash University, Clayton, Victoria, Australia; 3 Geosciences, Museum Victoria, Carlton, Victoria, Australia; 4 Plio-Pleistocene Palaeontology Section, Department of Vertebrates, Ditsong National Museum of Natural History, Pretoria, South Africa; University of Illinois at Urbana-Champaign, UNITED STATES

## Abstract

Nearly a century of paleontological excavation and analysis from the cave deposits of the Cradle of Humankind UNESCO World Heritage Site in northeastern South Africa underlies much of our understanding of the evolutionary history of hominins, other primates and other mammal lineages in the late Pliocene and early Pleistocene of Africa. As one of few designated fossil repositories, the Plio-Pleistocene Palaeontology Section of the Ditsong National Museum of Natural History (DNMNH; the former Transvaal Museum) curates much of the mammalian faunas recovered from the fossil-rich deposits of major South African hominin-bearing localities, including the holotype and paratype specimens of many primate, carnivore, and other mammal species (Orders Primates, Carnivora, Artiodactyla, Eulipotyphla, Hyracoidea, Lagomorpha, Perissodactyla, and Proboscidea). Here we describe an open-access digital archive of high-resolution, full-color three-dimensional (3D) surface meshes of all 89 non-hominin holotype, paratype and significant mammalian specimens curated in the Plio-Pleistocene Section vault. Surface meshes were generated using a commercial surface scanner (Artec Spider, Artec Group, Luxembourg), are provided in formats that can be opened in both open-source and commercial software, and can be readily downloaded either via an online data repository (MorphoSource) or via direct request from the DNMNH. In addition to providing surface meshes for each specimen, we also provide tomographic data (both computerized tomography [CT] and microfocus [microCT]) for a subset of these fossil specimens. This archive of the DNMNH Plio-Pleistocene collections represents the first research-quality 3D datasets of African mammal fossils to be made openly available. This simultaneously provides the paleontological community with essential baseline information (e.g., updated listing and 3D record of specimens in their current state of preservation) and serves as a single resource of high-resolution digital data that improves collections accessibility, reduces unnecessary duplication of efforts by researchers, and encourages ongoing imaging-based paleobiological research across a range of South African non-hominin fossil faunas. Because the types, paratypes, and key specimens include globally-distributed mammal taxa, this digital archive not only provides 3D morphological data on taxa fundamental to Neogene and Quaternary South African palaeontology, but also lineages critical to research on African, other Old World, and New World paleocommunities. With such a broader impact of the DNMNH 3D data, we hope that establishing open access to this digital archive will encourage other researchers and institutions to provide similar resources that increase accessibility to paleontological collections and support advanced paleobiological analyses.

## Introduction

**Fig 1:**
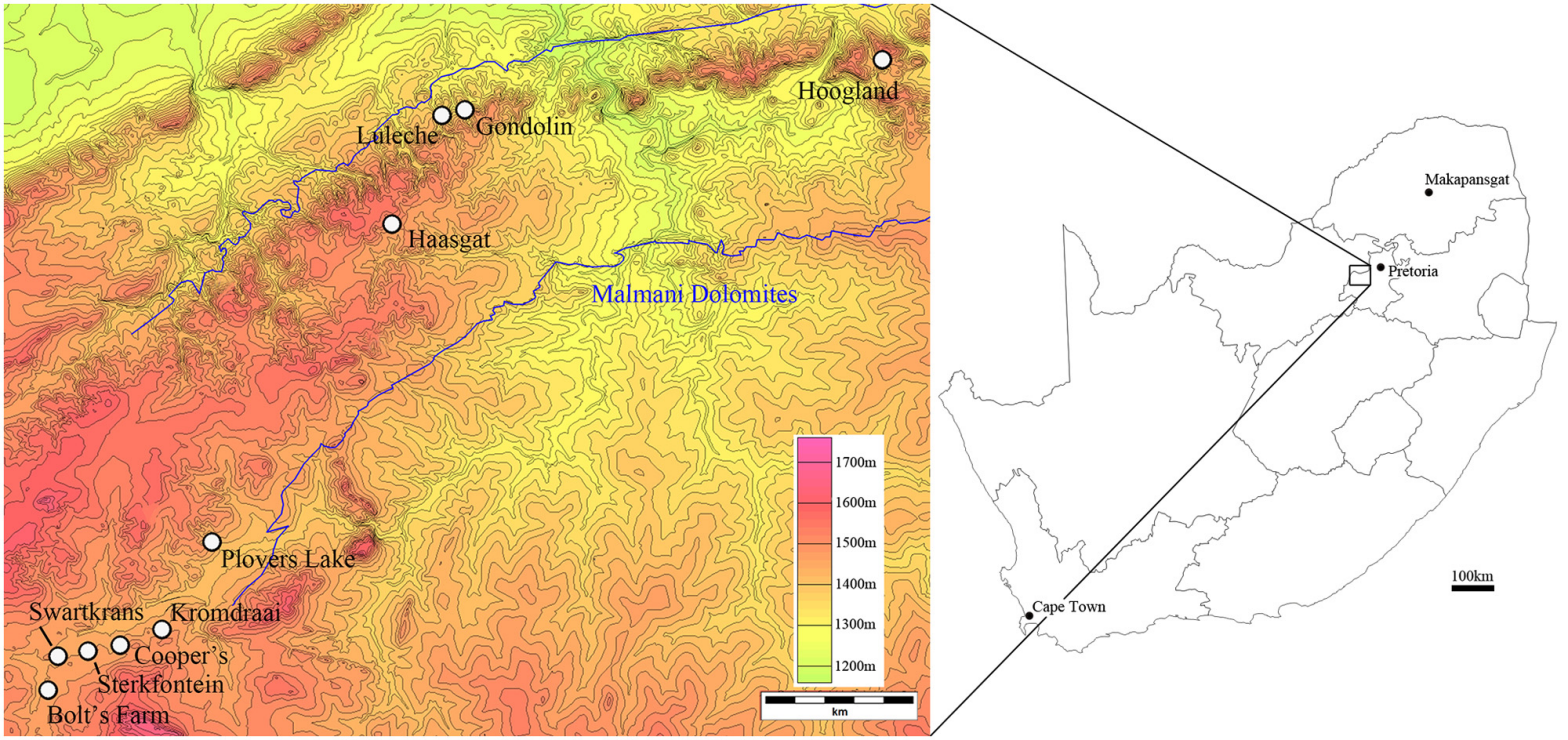
Topographic map of terminal Pliocene and Pleistocene fossil sites with fossil collections in the Ditsong National Museum of Natural History (left, contour lines equal 20m) relative to a schematic map of South Africa (right).

The Ditsong National Museum of Natural History (DNMNH; the former Transvaal Museum) is one of the primary repositories of Pliocene and Pleistocene fossils in South Africa, including much of the hominin and non-hominin faunas excavated from the paleokarstic deposits at Sterkfontein, Swartkrans, Kromdraai, Bolt’s Farm, Gondolin, Luleche, and Hoogland; and parts of the Taung, Cooper’s, Plover’s Lake, and Haasgat sites (Fig 1) [1–7]. Included within these site assemblages are mammal holotype and paratype specimens that have been curated at the Museum since the early paleontological collections by Robert Broom in the 1930s (see [8–53] in S1 Table)[1,10,19]. Alongside the broader fossil collections from these localities, these holotype and paratype specimens are the foundation for understanding the structure of southern African mammal communities during the late Neogene and early Quaternary, adaptations and speciation events, and underlie decades of paleobiological and paleoecological interpretations of the region during this time period [1–52; 53–57].

This publication describes a new open-access database of three-dimensional (3D) surface scan and tomographic data (computerized tomography [CT] and microfocus [microCT]) of the non-hominin primate and other faunal specimens curated in the Plio-Pleistocene Palaeontology Section (Department of Vertebrates) of the DNMNH. Establishing this non-hominin digital data resource for paleontological research compliments the recent publication of the surface and tomographic archive of Kromdraai B hominin specimens [58–59], and addresses several of the authors’ outstanding research and logistical goals. First, to provide an updated record of all the late Pliocene and Pleistocene mammal holotype and paratype specimens to the greater paleontological community. Second, to make baseline digital data available on these specimens that, while playing significant roles in past and current paleontological research, have had substantial variability in the quality and quantity of the primary/secondary description, measurements and imagery in the published literature. Third, to generate a 3D record of these specimens in their current state of preservation, particularly as most have not been previously molded and made available for researchers outside of the collections, and some of the specimens are extremely fragile (several have become damaged since their initial description). Fourth, to widely disseminate high-resolution digital data to minimize unnecessary duplication of data collection by researchers and improve accessibility in light of the funding and logistic challenges of overseas data collection (particularly for individual/small samples of specimens). This is one of few open-access, research-focused digitisation initiatives we are aware of from an African institution (the only other published to date being [58–59]) and is consistent with several global collection digitisation initiatives (e.g., British Museum [http://sketchfab.com/britishmuseum]; Cyark [http://www.cyark.org]; Digital Morphology Museum, KUPRI [http://dmm.pri.kyoto-u.ac.jp/dmm/WebGallery/index.html]; Digital Archive of Fossil Hominoids [http://www.virtual-anthropology.com/3d_data/3d-archive]; European Research Synchrotron Facility [http://paleo.esrf.eu]; European Virtual Anthropology Network-Society [http://www.evan-society.org]; Kenya National Museums [http://www.africanfossils.org]; [Lincoln 3D Scans [http://lincoln3dscans.co.uk]; Metropolitan Museum of Art [http://www.thingiverse.com/met/about]; MorphoSource platform [http://www.morphosource.org/]); NESPOS Neanderthal Studies Professional Online System [http://www.nespos.org/]; 3D Petrie Museum [http://www.ucl.ac.uk/3dpetriemuseum]; Presious [http://www.presious.eu]; PRIMO, NYCEP PRImate Morphometrics Online database [http://primo.nycep.org]; Smithsonian X3d [http://3d.si.edu/]) that are providing novel methods for institutional collections access. And fifth, to encourage a phase of imaging-based paleobiological research across a range of South African fossil faunas consistent with the current, complex morphological methods (e.g., geometric morphometrics) that have become standard in analysing hominin and other primate species [60–69].

## Materials and Methods

The Plio-Pleistocene Section of the Department of Vertebrates, Ditsong National Museum of Natural History has granted permission for digitisation of their reposited fossil specimens. The DNMNH Plio-Pleistocene Palaeontology Section collections include thousands of taxonomically-identifiable fossil mammal specimens [1, 4–7, 70]. Here, we have begun our digital archive with all 89 non-hominin holotype, paratype and key mammalian specimens (Orders Primates, Carnivora, Artiodactyla, Eulipotyphla, Hyracoidea, Lagomorpha, Perissodactyla, and Proboscidea) held in the Plio-Pleistocene Section vault (‘Broom Room’; S1 Table). This archive of digitised specimens will be expanded annually by incorporating additional surface models and tomographic datasets of non-hominin specimens from the greater Section collections, particularly those mammalian groups (e.g. Order Artiodactyla, Order Perissodactyla) with minimal representation in the vault subset.

There are several methods and technologies for generating 3D digital data of museum specimens, each which vary in resolution, accuracy, speed of capture and specimen size capacity. Here we have made use of the commercially-available Artec Spider surface scanner (Artec Group, Luxembourg; http://www.artec3d.com/hardware/artec-spider/) as the primary method of digitising visible surface morphology. The Artec Spider was selected for use in our digitising initiative for several reasons. First, it has a manufacturer-stated point accuracy (0.05mm) and mesh resolution (0.1mm) that is consistent with, or greater than, the manufacturer-stated point accuracy and resolution of other commercially-available, transportable full-color surface scanners (e.g., NextEngine, NextEngine Inc., USA [http://www.nextengine.com/products/scanner/specs#]; Mephisto EX-PRO, 4DDynamics, Belgium [http://www.4ddynamics.com/3D-scanners/ex-pro/]; HDI Advance, LMI Technologies, Canada [http://lmi3d.com/products/hdi-advance#]; Go!SCAN, Creaform, Canada [http://www.creaform3d.com/en/metrology-solutions/handheld-portable-3d-scanner-goscan-3d]; SLS–2, DAVID Group, Germany [http://www.david-3d.com/en/products/sls-2]; also see comparisons in [71]). Second, the scanner is a lightweight (0.85kg) handheld device that can operate without tripod or linked turntable, and it does not require pre-scan calibration or reflective surface targets that could impact preservation of the fossil. Third, the integration of a blue LED light source improves the capture of geometry from dark/black surfaces (e.g. manganese-stained fossils from karstic deposits) that are difficult to capture with white light scanners [72]. And fourth, the Artec Spider captures both geometry and texture (color) data (1.3 megapixel, 24-bits per pixel), allowing us to provide researchers with important visual cues about the specimens (e.g., cortical weathering, staining, plaster and glue reconstruction/repair).

Surface mesh generation with the Artec Spider made use of the native scanner software (Artec Studio 9) and followed the standard scan acquisition and workflow process outlined in the manufacturer provided user documentation (Artec Group, 2013). In order to fully capture the external morphology, each individual fossil specimen was scanned in multiple passes with significant overlap in object coverage to allow for successful individual scan alignment and registration. Generally, fossils were placed on medium-density polyurethane foam that not only supported and protected the specimen during scanning, but also exhibited minimal reflectivity to the scanner light source (e.g., an essentially ‘invisible’ platform that sped up post-scan processing). Completed individual scan passes were automatically subjected to a fine serial registration prior to being manually edited to remove noise and/or background geometry. Because individual scans lack relative position information, each edited scan pass was then aligned (rigid alignment) based on user-defined points (minimally 3 shared points). These roughly aligned scans were then globally registered (default settings: minimal distance between adjacent feature points: 10mm; number of algorithm iterations: 100) to produce a single coordinate system for captured points. Any remaining outlying points were then removed and a single polygonal 3D mesh fused using the Sharp Fusion algorithm (resolution of triangulation grid: 0.2mm). Most of the resulting 3D meshes were retained at the originally-generated polygon count, although some of the larger specimen meshes were reduced using the Mesh Simplification algorithm (triangle quantity method; target number: 3.5 million triangles) to reduce file size without compromising accuracy. Finally, texture was applied to the 3D mesh using the texture atlas method (4096 x 4096) which generates a single texture file linked to the mesh.

Three of the Artec Spider-generated meshes (SK 554: *Dinopithecus ingens*; KA 58: *Crocuta crocuta* [occipital fragment only]; KA 48: *Procavia transvaalensis*) required post-processing to manually reconstruct regions not collected during scanning to yield a watertight surface. The STL polygon meshes were imported into Geomagic Studio 2014 (3D Systems, USA) and edited to correct for scan artefacts and fill areas of the mesh surface. These regions not captured by the Artec Spider do not represent traditionally measured or analysed features and we do not expect this alteration to impact analysis outcomes (SK 554: endocranial surfaces of the reconstructed neurocranial vault; KA 58: posterior cranial fossa superior to the tentorium cerebelli; KA 48: posterior right orbit broken and open to the endocranial surface). By default, the reconstructed polygon meshes are available in the digital archive; however, unreconstructed STLs of these specimens can be made available upon request.

The 3D meshes generated by the Artec Spider have been saved (and are available) as a geometry-only stereolithography file (STL) and a geometry-and-texture virtual reality modelling language 2.0 file (VRML; with linked portable network graphics [PNG] texture file) directly from Artec Studio 9. Both the STL and VRML file types have been opened in several software programs to ensure compatibility (Rhinoceros 5, Robert McNeel and Associates, USA; Geomagic Studio 2014; Meshlab 1.3.3 [www.meshlab.sourceforge.net]). In order to provide a ‘single file’ version of the textured mesh that would also be compatible with 3D morphometric analysis software packages (e.g., Landmark Editor 3.6, Institute for Data Analysis and Visualization [73]), as well as compatible with the MorphoSource database 3D viewer (which does not currently support VRML), a textured polygon file (PLY) was created from the VRML in Meshlab 1.3.3 (using the ‘Transfer Color: Texture to Vertex’ filter).

In addition to the Artec Spider-produced surface meshes we are also making available tomographic datasets produced from prior research projects on the DNMNH Plio-Pleistocene Section collections (S1 Table: underlined specimens; also additional specimens not provided as surfaces, see S1 Table: Footnote ‘f’). Computerized tomography scans of fossil suid specimens were produced in 2006 using a Phillips Brilliance^TM^ 180P_Z_ (1mm slice depth, 0.5mm interslice distance [voxel size: 0.42mm x 0.42mm x 0.50mm]; 140 kV, 122 mA) at the Donald Gordon Medical Centre (University of the Witwatersrand Medical School, Johannesburg). MicroCT scans of fossil primate specimens were produced in 2013 and 2014 using the Nikon XTH 225 ST micro-focus X-ray tomography machine at the Micro-Focus X-Ray Tomographic Facility (MIXRAD) at the South African Nuclear Energy Corporation (Necsa) (Pretoria). Resulting isometric voxel sizes for these primate specimens are listed in S1 Table. Because of difficulties encountered in resolving the endocranial contours of the SK 559 (*Dinopithecus ingens*), CO 100 (*Papio angusticeps*), and STS 394a&b (*Cercopithecoides williamsi*) specimens with the Artec Spider we have made use of the microCT data to generate polygonal mesh surfaces. Scan data was manually thresholded to produce an isosurface of the specimen in Avizo Standard 8.1(Visualization Sciences Group, FEI Company, USA) that excluded background noise but was inclusive of any matrix. The surface generation module (‘constrained smoothing’) was used to generate the polygonal mesh, which was then simplified to ~ 3.5 million faces to reduce file size. The resulting STL was then imported into Artec Studio 9 and used as the surface for texture application (where the imported microCT STL was aligned to the Artec Spider surface scans gathered from these specimens and all visible surfaces textured using the texture atlas method described above).

As discussed by Skinner et al. (2013) in their publication of the Kromdraai B hominin surface and tomographic archive, both equipment-induced effects and user-defined methods influence the resulting surface model from currently employed imaging [58]. Variations in surface scanning protocols, halo effects, thresholding CT/microCT datasets, and other methodological choices (global registration, fusion and mesh simplification algorithm settings) mean that subtly different polygonal meshes could be produced from these same specimens. While it is difficult to directly gauge the fidelity of the resulting digital surfaces (derived from either tomographic datasets or surface scanners) to the original fossil specimens, we have undertaken a limited assessment of the validity of the Artec Spider generated 3D surface meshes to demonstrate their potential applicability for palaeobiological research against the more widely used and published sources for 3D surface data: those generated by thresholding CT and microCT datasets (Table 1). Surface scan-derived polygonal mesh surfaces (STL) from six of the specimens included in our archive were imported into Geomagic Studio 14 and aligned (N-point Alignment; number of points used varied across specimens) and globally registered (Global Registration module; default settings) with a polygonal mesh surface (STL) of the same specimen generated from either the archived CT or microCT datasets (Table 1). Once aligned and registered, the variance between the meshes (e.g., distances between the aligned vertices of CT/microCT mesh and the surface scan mesh) produced were assessed using the Deviation module (default settings; CT or microCT mesh as the ‘Reference’ and surface scanned mesh as the ‘Test’). This module quantifies the deviation (in millimetres) between the aligned vertices of the two meshes and reports the minimum and maximum distances (in this case, reflecting the negative or positive metric deviations of the surface scan mesh surface from the CT/microCT mesh), the average distance, the standard deviation, and the root-mean-square (RMS) estimate (Table 1).

**Table 1:** Comparisons of fossil specimen surfaces derived from the Artec Spider with those derived from CT/microCT (distances in millimetres).

Specimen	Threshold Values for Surface Mesh	Average Distance	Minimum Distance	Maximum Distance	Standard Deviation	RMS Estimate
KA 194 (microCT)	32–1257	-0.38	-2.06	3.44	0.36	0.53
BF 1 (Left Mandible; CT)	-230–3095	-0.17	-2.72	2.34	0.21	0.26
BF 1 (Maxilla; CT)	-486–3095	-0.23	-2.48	3.70	0.24	0.34
SK 4005 (CT)	1027–4095	-0.07	-2.24	3.07	0.24	0.25
KA 89A (Cranium; CT)	-261–3095	-0.12	-2.66	4.89	0.40	0.41
KA 89B (Mandible; CT)	-480–3095	-0.20	-2.31	1.07	0.15	0.25

## Results

**Fig 2:**
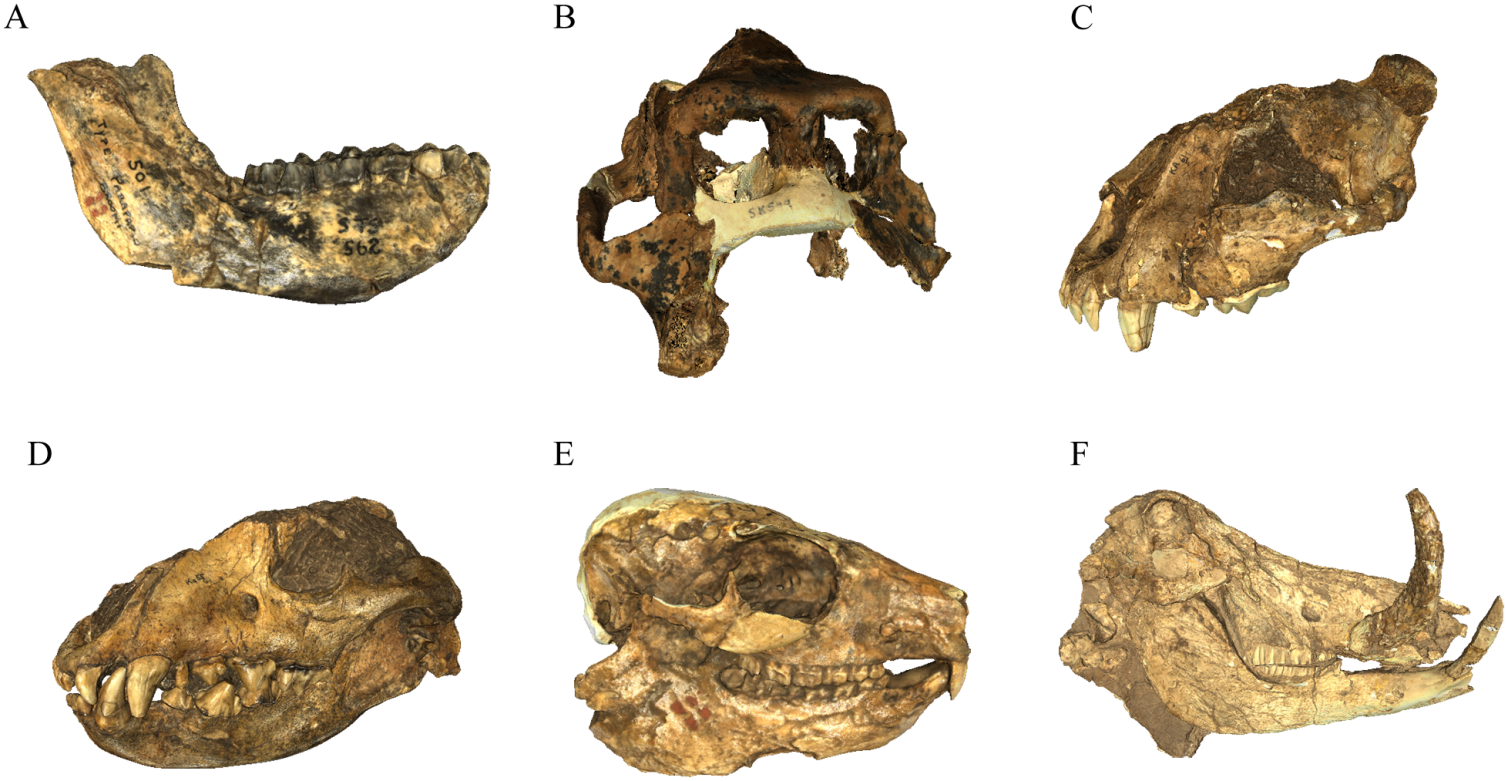
Screenshots of some representative specimens from the DNMNH archive. A, *Parapapio broomi* (STS 562); B, *Papio* (*Dinopithecus*) *ingens* (SK 599); C, *Dinofelis piveteaui* (KA 61); D, *Pachycrocuta brevirostris* (KA 55); E, *Procavia antiqua* (ST 105); F, *Phacochoerus antiquus* (BF 3–335). Images of all archive specimens are available online through MorphoSource and at http://www.sapalaeo.com/dnmnh-archive.

The list of specimens curated in the DNMNH Plio-Pleistocene vault and available through our digital archive as polygonal mesh surfaces (PLY, STL, VRML) and/or tomographic dataset is presented in S1 Table (with representative screenshots provided in Fig 2). In lieu of redescribing this wide range of specimens within this publication or in S1 Table, we have instead provided the primary (and secondary) references to the previously published original/expanded specimen and taxonomic descriptions as a resource for guiding use of the offered data. Our archive of digital data (consisting of a screenshot of the textured polygonal mesh, each version of the surface mesh, and any available tomographic data) are openly available for researchers and can be accessed through two different paths. First, all STL, PLY and tomographic data has been uploaded to the MorphoSource platform (http://www.morphosource.org; DOI for all files provided in S2 Table) as a project administered by two of the current authors (JWA, SP) and can be requested through the MorphoSource website as direct download. Second, screenshots and details of the archive project, along with direct email links to request digital data download permission of all file types (STL, PLY, VRML, CT, microCT), are provided through our research group website (http://www.sapalaeo.com/dnmnh-archive). Independent of the origin of the request, researchers are required to complete a digital data agreement form (including a brief research proposal) directly to the curator of the Plio-Pleistocene Section (Stephany Potze: potze@ditsong.org.za; stephany.potze@gmail.com [preferred]; digital data agreement form available via email or at http://www.sapalaeo.com/dnmnh-archive). This policy of documenting specimen use is consistent with current on-site and digital fossil access protocols of the DNMNH Plio-Pleistocene Palaeontology Section, ensures adherence to the guidelines for acceptable use of the specimens (e.g., strictly non-commercial, research and educational purposes only), and allows the Museum to retain records of specimen use across research projects essential for complying with South African government Acts governing the use of curated heritage objects. Researchers who make use of this surface and tomographic archive are also asked to cite this publication as the source of the digital data, which serves to both acknowledge the personal and institutional resources underlying the project and allows the Plio-Pleistocene Palaeontology Section to monitor the use and publication of their collections. Researchers making use of the microCT data generated from the Nuclear Energy Corporation of South Africa (Necsa) are asked to acknowledge in publication: ‘MicroCT data from the Necsa MIXRAD facility was generated using a Nikon XTH 225 ST micro-focus X-ray tomography system funded by the Department of Science and Technology and National Research Foundation.’

**Fig 3:**
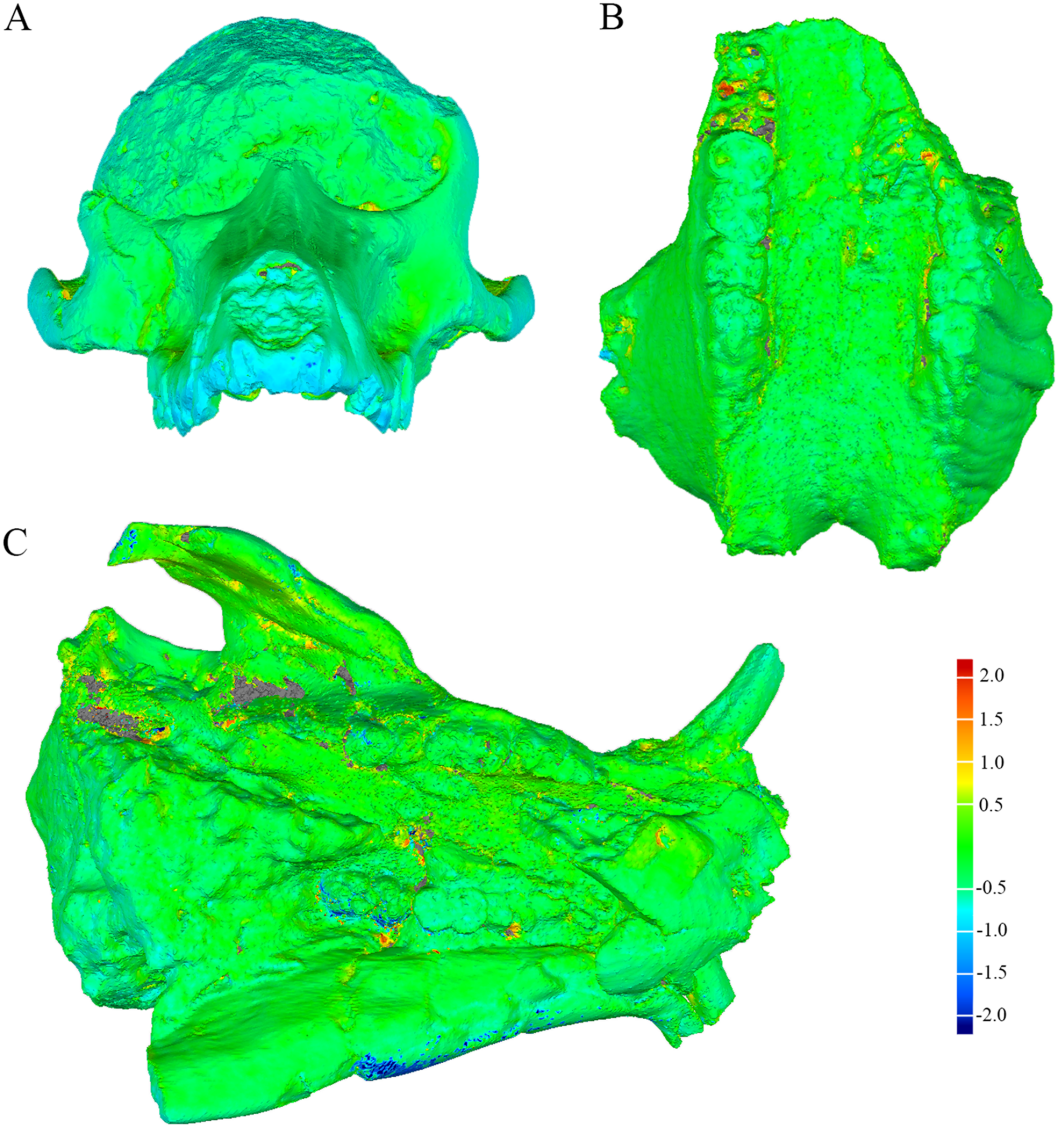
Screenshots of surface deviation maps of specimen meshes derived from tomographic datasets and the Artec Spider scanner. The color spectrum scale represents positive/negative deviations in millimetres. Positive deviations reflect the Artec Spider-derived surface being inflated relative to the CT or microCT surface; negative deviations reflected regions where the Artec Spider-derived surface is depressed relative to the CT or microCT surface. A, anterior view of KA 194 *Papio angusticeps* cranium. B, inferior view of SK 4005 *Phacochoerus antiquus* maxilla. C, inferior view of KA 89 *Phacochoerus antiquus* skull (available via Supporting Information).

As an example of the data available from this archive the computerised tomography dataset (TIFF stack; S1 Dataset) and surface meshes (STL, PLY; S2–S4 Datasets) for the type specimen of *Phacochoerus antiquus* (KA 89; Order Artiodactyla: Family Suidae) have been provided as Supporting Information. The results of our limited assessment of the validity of the surfaces generated through the Artec Spider and CT/microCT are summarized in Table 1, with selected screenshots of the surface distance maps provided in Fig 3. The average surface distances are all below 0.4mm and the root mean square (RMS) estimates are low. The minimum and maximum distances, however, indicate the presence of some strong metric variance between paired surface meshes derived from the Artec Spider and tomographic datasets. Our visual inspection of the distance maps indicates that the regions with the most metric deviation between surfaces are extremely limited and are also those in ‘inaccessible’ parts of the specimen (e.g., the deep termination of alveolus surrounding dentition, deep pockets of matrix or undercuts; Fig 3). Although these results suggest minimal difference in the surfaces derived from these methods, ultimately we advocate the same caution as [59] (and summarised above) that each researcher will have to determine if the archived surfaces and datasets are of sufficient fidelity and appropriate for their specific methods and hypothesis testing.

## Discussion

Testing complex functional, ontogenetic and phylogenetic hypotheses from organismal anatomy necessitates comprehensive datasets and analysis methods that adequately and consistently compare expressed morphology [60–69, 74–75]. Improved access to imaging technologies, advances in personal computing power, and software and statistical methods for 3D data capture and analysis over the past 30 years have significantly expanded the available ‘digital toolkit’ for paleontologists (among other disciplines) to address such complex hypotheses in their research. While not supplanting the central role of primary description or linear metrics, the integration of these imaging-based 3D datasets and methods over the decades have yielded (and continue to open) novel anatomical features and biological systems for paleontological inquiry [76–90].

Whether initiated by researchers or institutions, the generation of accessible 3D digital data archives is increasingly serving a vital role in both preserving and opening collections of heritage objects, biological and paleontological specimens to the general public and scientists (online archives cited above; [58–59]). For the scientific community increased access to object 3D data can (depending on the scope, resolution and imaging method) enhance study impact by increasing analysis sample size and diversity and/or reduce the time and financial costs related to data collection. We view the comprehensive scope and resolution of the DNMNH digital archive we have produced and described here as having a significant impact for both paleontological research logistics and outcomes. In terms of research logistics, the scanned fossil specimens included in the archive represent a significant advance towards providing greater access to the Plio-Pleistocene Palaeontology Section collections. With the budgetary limitations of competitive grant funding in paleontological research, overseas data collection at African institutions can be prohibitive; particularly for pilot projects, postgraduate (doctoral) research, or analyses of lineages where only single individuals or small sample sizes are curated in the collections. Although the surfaces and tomographic data presented here may not be suitable for all research projects requiring digital imaging of specimen morphology, we suggest that this archive will minimally provide a resource for undertaking preliminary paleobiological analyses (increasingly important in obtaining competitive grant funding) and obviate some duplication of equipment, time, effort and resources. Future additions to this open archive (both through surface scanning and increased addition of microtomographic imaging) will further enhance the impact by focusing on mammalian groups that are not represented by the DNMNH holotypes/paratypes but are well-represented in the larger Section fossil collections.

Critically, in terms of the potential impacts of this data on research outcomes, the DNMNH digital archive provides the first baseline 3D data on types, paratypes, and key specimens of widely distributed extinct carnivore groups (*Dinofelis*, *Megantereon*, *Pachycrocuta*) [35], the latest occurrence of the once widely-distributed hunting hyenas (*Chasmaporthetes* and *Lycyaenops*) [35], and the earliest representatives of the extant baboon (Genus *Papio*) and warthog (Genus *Phacochoerus*) in the fossil record [43, 91–92]. As such, this digital archive of the DNMNH vault specimens not only provides basic 3D morphological data on taxa fundamental to Neogene and Quaternary South African paleontology, but also for mammalian lineages integral within African, Old World, and New World paleocommunities [56, 93–94]. With such a potentially broader impact of the 3D data offered here, and echoing prior digital fossil archive projects [58–59] and the goals of data access platforms like MorphoSource, we hope that providing open access to this digital archive (in its current form and through future expansion) will encourage other researchers and institutions to provide similar resources that increase collections accessibility and support advanced paleobiological analyses.

## Supporting Information

S1 Dataset Computerised tomography dataset for the type specimen of *Phacochoerus antiquus* (KA 89; Order Artiodactyla: Family Suidae). (ZIP)

S2 Dataset Full colour surface mesh (PLY) for the cranium of the type specimen of *Phacochoerus antiquus* (KA 89a; Order Artiodactyla: Family Suidae). (ZIP)

S3 Dataset Surface mesh (STL) for the cranium of the type specimen of *Phacochoerus antiquus* (KA 89a; Order Artiodactyla: Family Suidae). (ZIP)

S4 Dataset Surface meshes (STL, PLY) for the mandible of the type specimen of *Phacochoerus antiquus* (KA 89b; Order Artiodactyla: Family Suidae). (ZIP)

S1 Table Surface scan database of non-hominin holotype, paratype and major faunal specimens from the Plio-Pleistocene Section collections, Ditsong National Museum of Natural History. Holotype specimens are in **bold**. Those specimens with additional available tomographic data are underlined.
(DOCX)

S2 Table DOI for the surface meshes and tomographic data in the Ditsong National Museum of Natural History Archive lodged with MorphoSource. (DOCX)
