# Supplementary figures and images for: Surface Model and Tomographic Archive of Fossil Primate and Other Mammal Holotype and Paratype Specimens of the Ditsong National Museum of Natural History, Pretoria, South Africa (part 2 of 2)
Source: PLoS One. 2015 Oct 6;10(10):e0139800. doi: 10.1371/journal.pone.0139800 (PMC4595468; doi:10.1371/journal.pone.0139800)

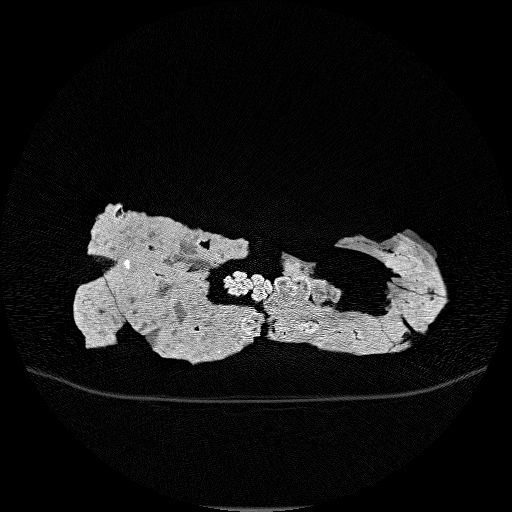

Supplement: S1 Dataset — (ZIP) [file pone.0139800.s001.zip › KA89/KA89A/KA890385.tif]

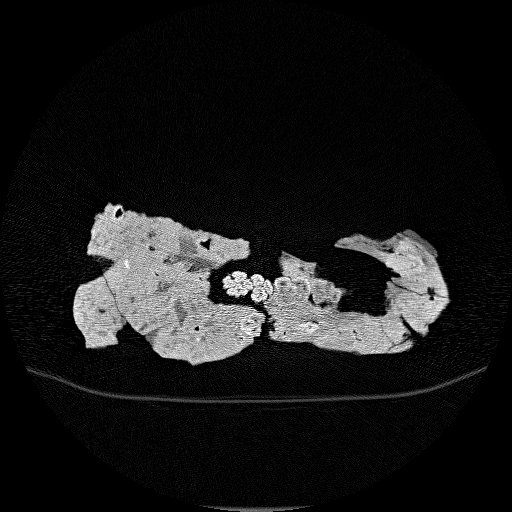

Supplement: S1 Dataset — (ZIP) [file pone.0139800.s001.zip › KA89/KA89A/KA890386.tif]

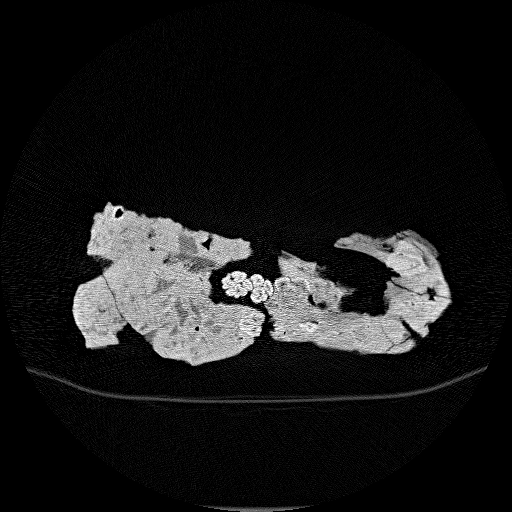

Supplement: S1 Dataset — (ZIP) [file pone.0139800.s001.zip › KA89/KA89A/KA890387.tif]

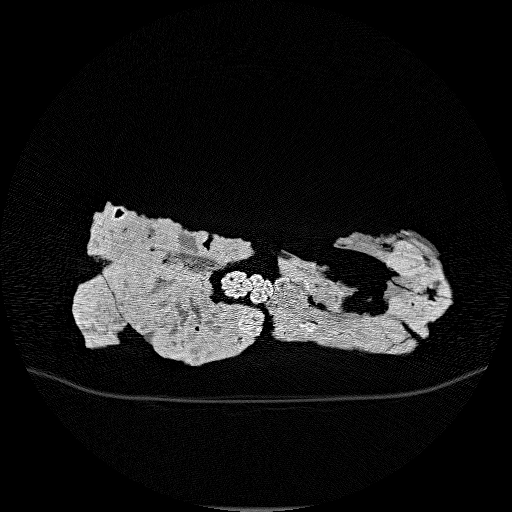

Supplement: S1 Dataset — (ZIP) [file pone.0139800.s001.zip › KA89/KA89A/KA890388.tif]

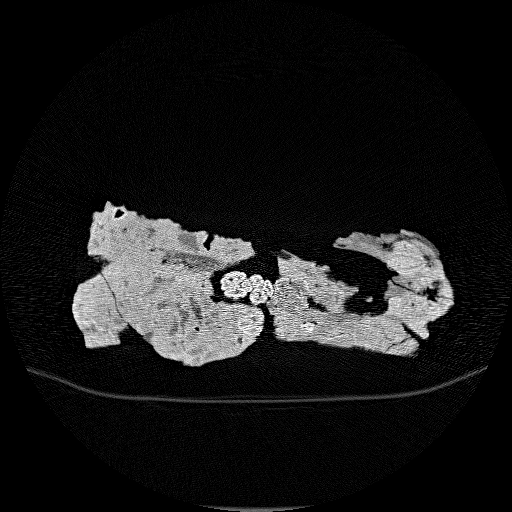

Supplement: S1 Dataset — (ZIP) [file pone.0139800.s001.zip › KA89/KA89A/KA890389.tif]

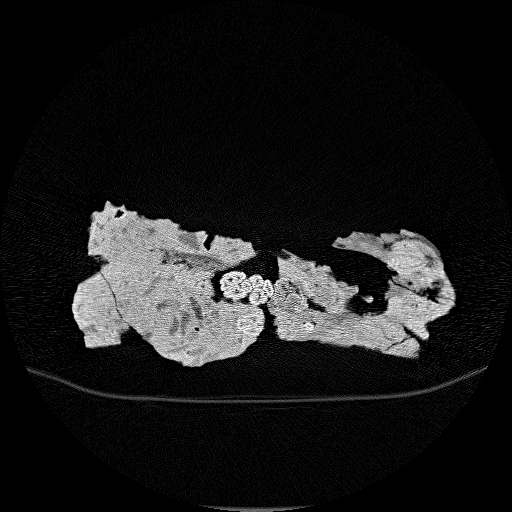

Supplement: S1 Dataset — (ZIP) [file pone.0139800.s001.zip › KA89/KA89A/KA890390.tif]

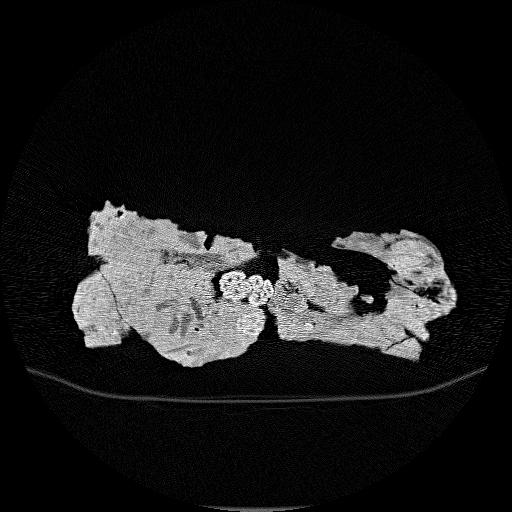

Supplement: S1 Dataset — (ZIP) [file pone.0139800.s001.zip › KA89/KA89A/KA890391.tif]

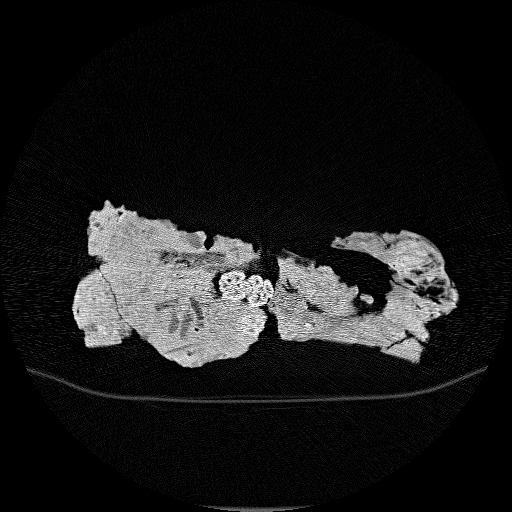

Supplement: S1 Dataset — (ZIP) [file pone.0139800.s001.zip › KA89/KA89A/KA890392.tif]

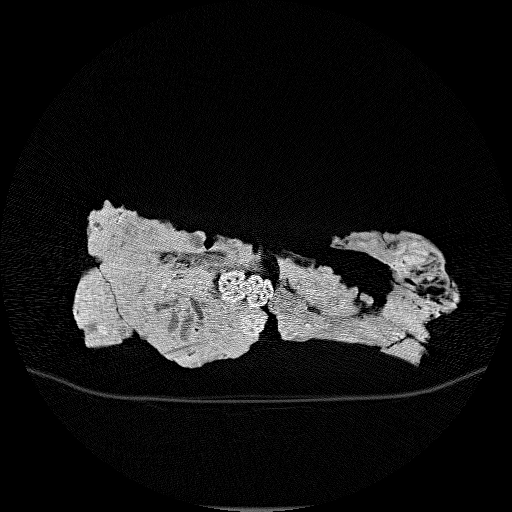

Supplement: S1 Dataset — (ZIP) [file pone.0139800.s001.zip › KA89/KA89A/KA890393.tif]

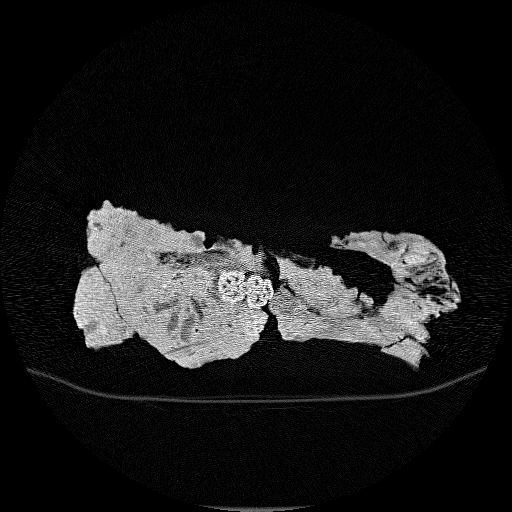

Supplement: S1 Dataset — (ZIP) [file pone.0139800.s001.zip › KA89/KA89A/KA890394.tif]

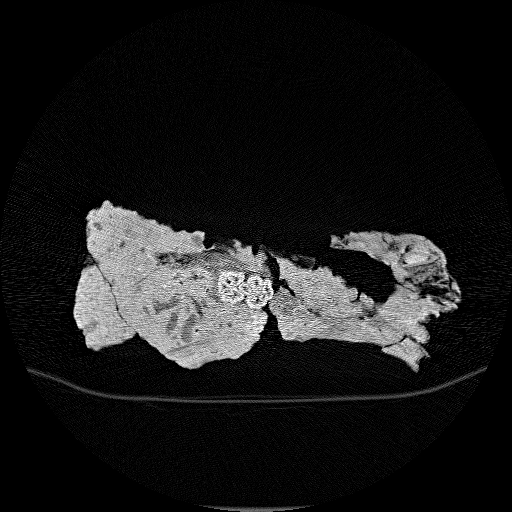

Supplement: S1 Dataset — (ZIP) [file pone.0139800.s001.zip › KA89/KA89A/KA890395.tif]

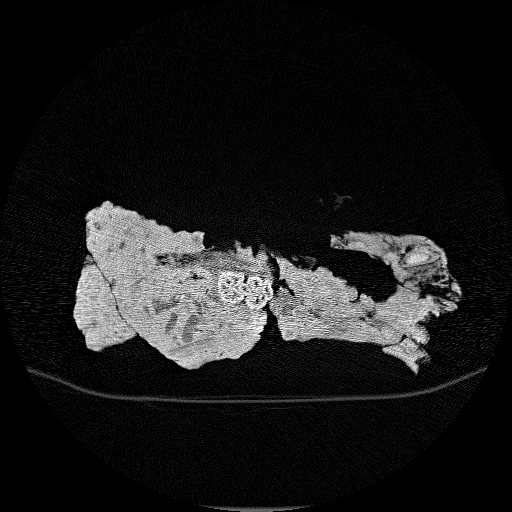

Supplement: S1 Dataset — (ZIP) [file pone.0139800.s001.zip › KA89/KA89A/KA890396.tif]

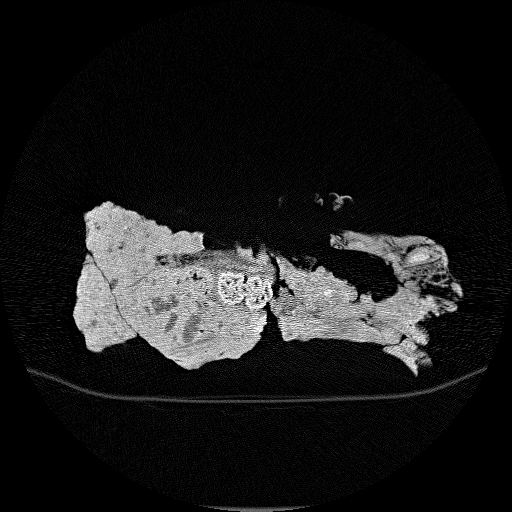

Supplement: S1 Dataset — (ZIP) [file pone.0139800.s001.zip › KA89/KA89A/KA890397.tif]

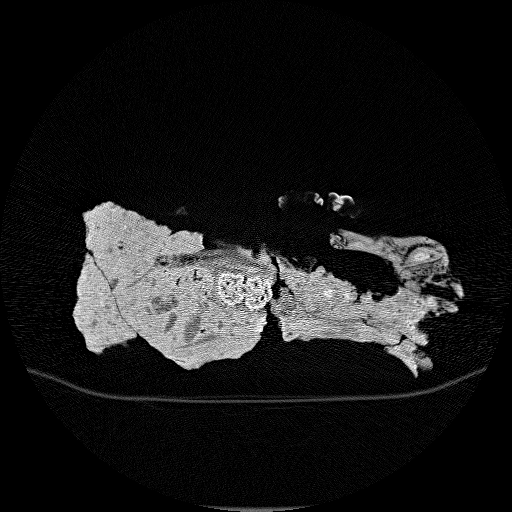

Supplement: S1 Dataset — (ZIP) [file pone.0139800.s001.zip › KA89/KA89A/KA890398.tif]

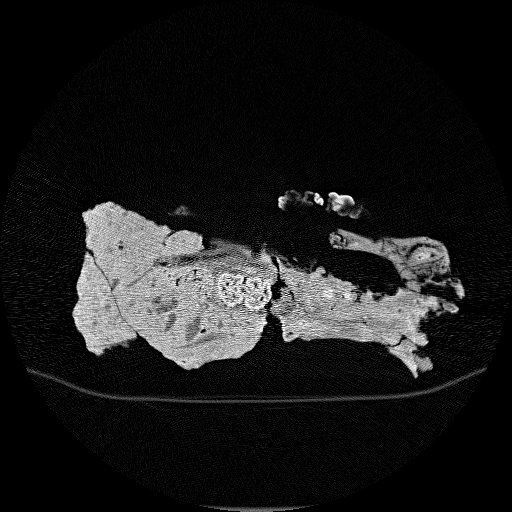

Supplement: S1 Dataset — (ZIP) [file pone.0139800.s001.zip › KA89/KA89A/KA890399.tif]

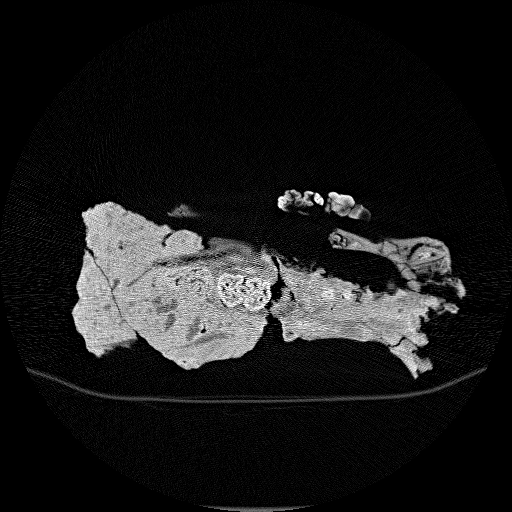

Supplement: S1 Dataset — (ZIP) [file pone.0139800.s001.zip › KA89/KA89A/KA890400.tif]

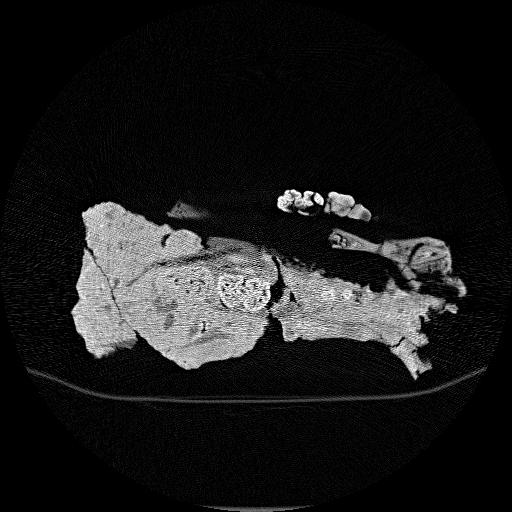

Supplement: S1 Dataset — (ZIP) [file pone.0139800.s001.zip › KA89/KA89A/KA890401.tif]

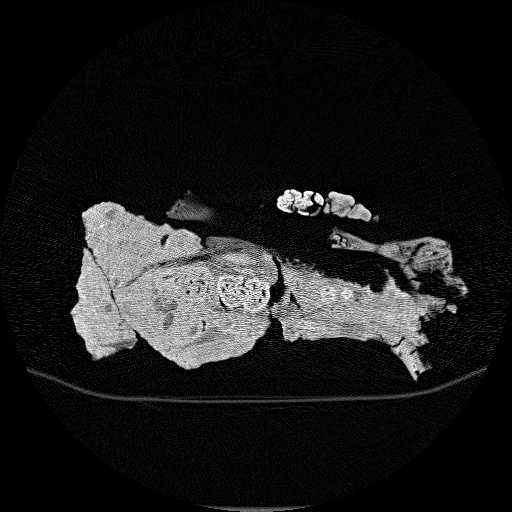

Supplement: S1 Dataset — (ZIP) [file pone.0139800.s001.zip › KA89/KA89A/KA890402.tif]

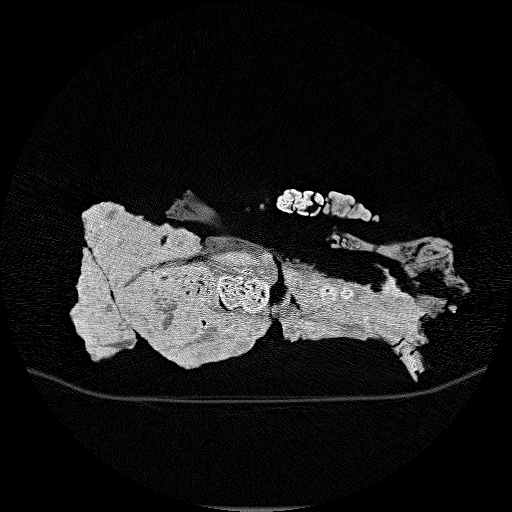

Supplement: S1 Dataset — (ZIP) [file pone.0139800.s001.zip › KA89/KA89A/KA890403.tif]

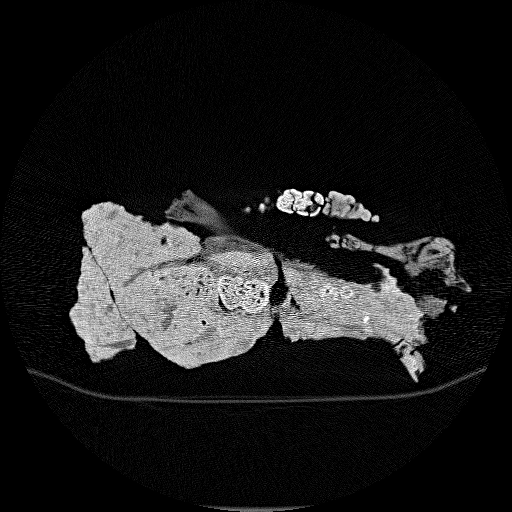

Supplement: S1 Dataset — (ZIP) [file pone.0139800.s001.zip › KA89/KA89A/KA890404.tif]

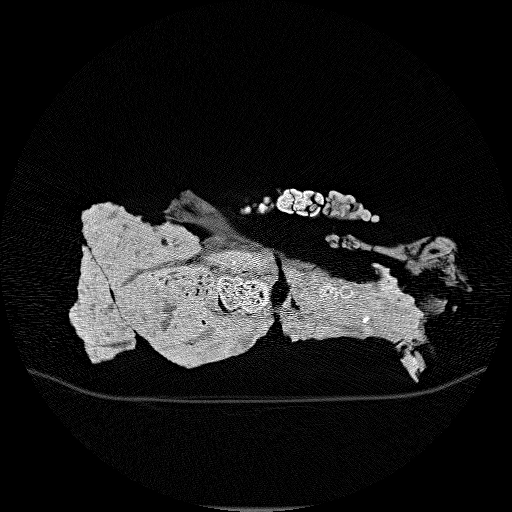

Supplement: S1 Dataset — (ZIP) [file pone.0139800.s001.zip › KA89/KA89A/KA890405.tif]

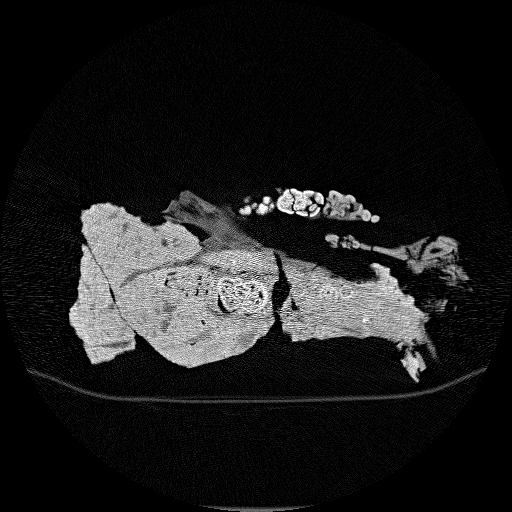

Supplement: S1 Dataset — (ZIP) [file pone.0139800.s001.zip › KA89/KA89A/KA890406.tif]

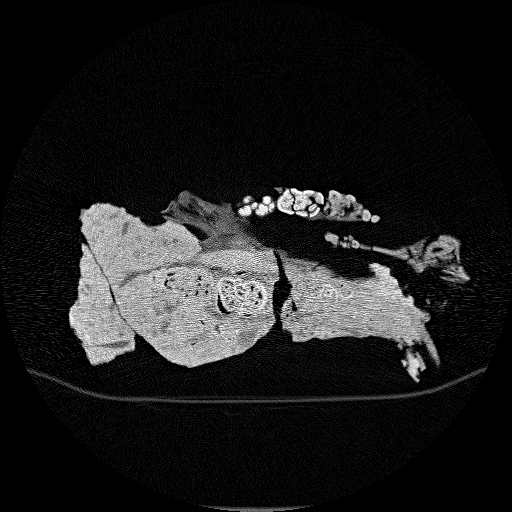

Supplement: S1 Dataset — (ZIP) [file pone.0139800.s001.zip › KA89/KA89A/KA890407.tif]

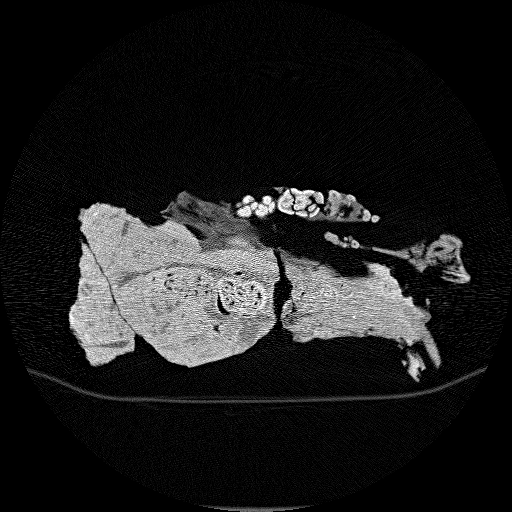

Supplement: S1 Dataset — (ZIP) [file pone.0139800.s001.zip › KA89/KA89A/KA890408.tif]

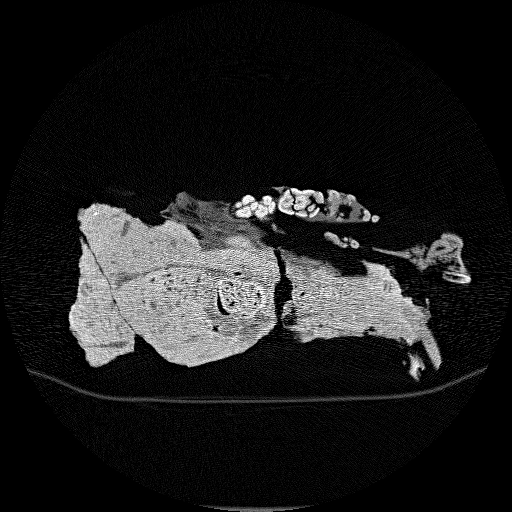

Supplement: S1 Dataset — (ZIP) [file pone.0139800.s001.zip › KA89/KA89A/KA890409.tif]

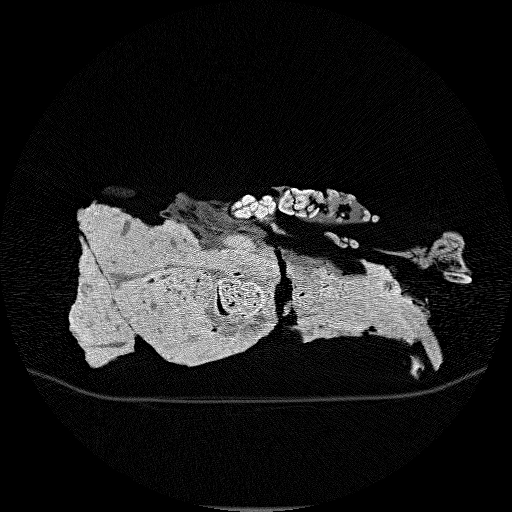

Supplement: S1 Dataset — (ZIP) [file pone.0139800.s001.zip › KA89/KA89A/KA890410.tif]

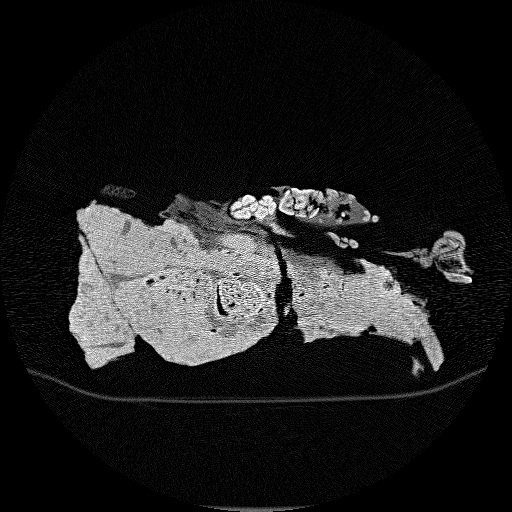

Supplement: S1 Dataset — (ZIP) [file pone.0139800.s001.zip › KA89/KA89A/KA890411.tif]

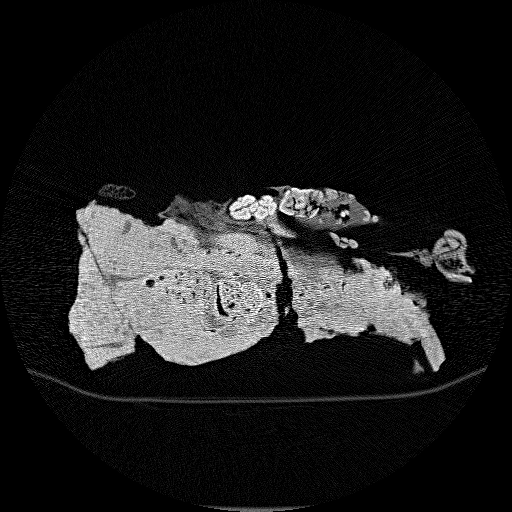

Supplement: S1 Dataset — (ZIP) [file pone.0139800.s001.zip › KA89/KA89A/KA890412.tif]

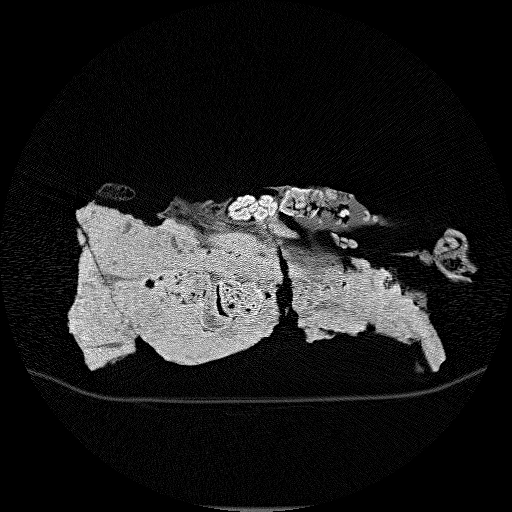

Supplement: S1 Dataset — (ZIP) [file pone.0139800.s001.zip › KA89/KA89A/KA890413.tif]

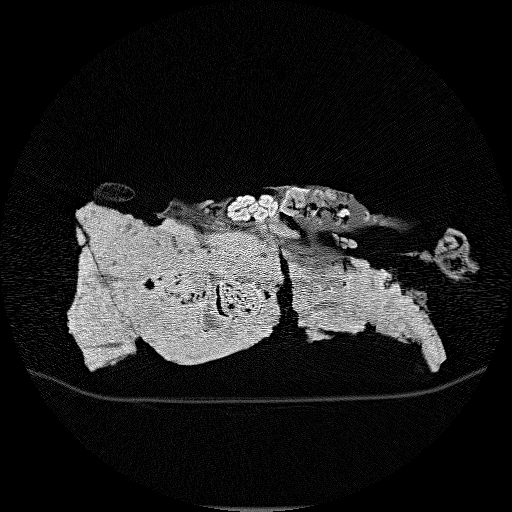

Supplement: S1 Dataset — (ZIP) [file pone.0139800.s001.zip › KA89/KA89A/KA890414.tif]

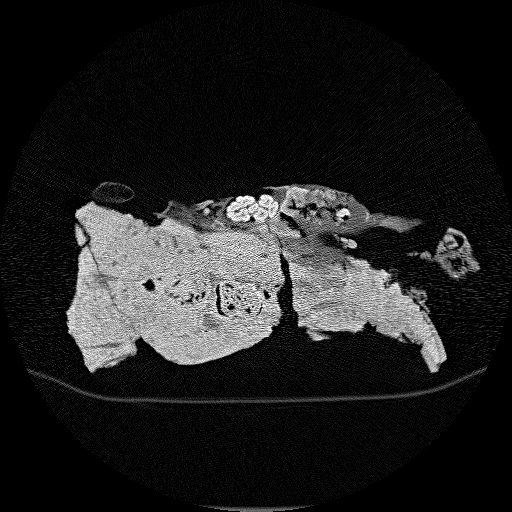

Supplement: S1 Dataset — (ZIP) [file pone.0139800.s001.zip › KA89/KA89A/KA890415.tif]

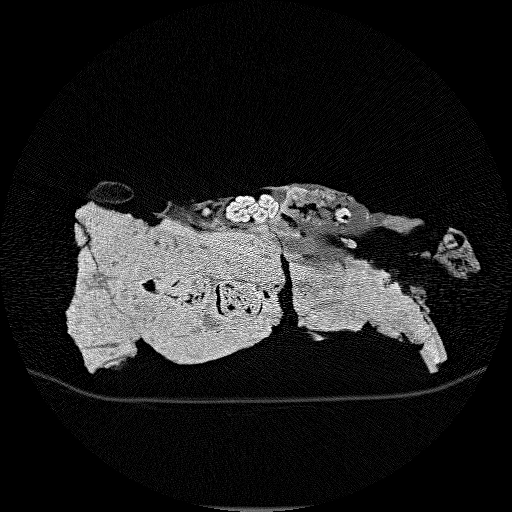

Supplement: S1 Dataset — (ZIP) [file pone.0139800.s001.zip › KA89/KA89A/KA890416.tif]

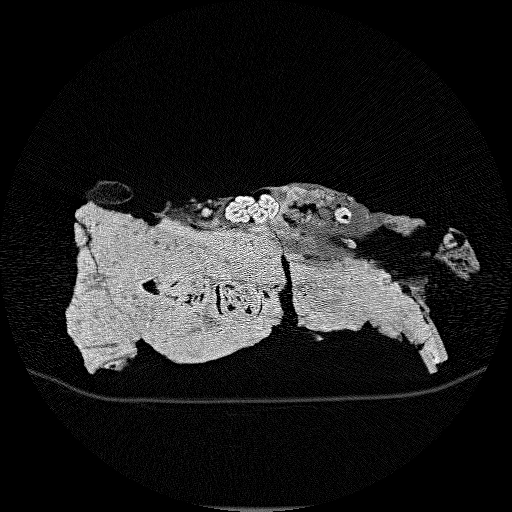

Supplement: S1 Dataset — (ZIP) [file pone.0139800.s001.zip › KA89/KA89A/KA890417.tif]

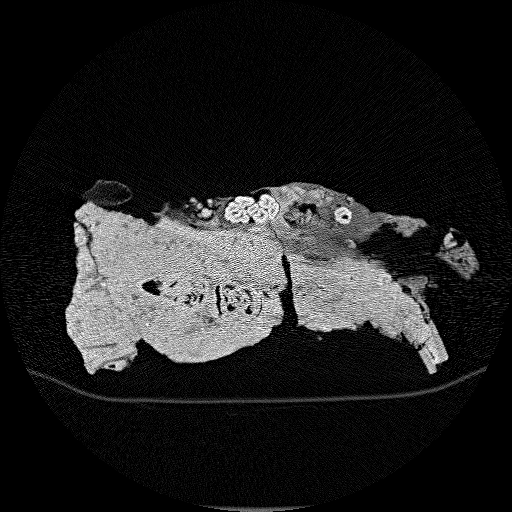

Supplement: S1 Dataset — (ZIP) [file pone.0139800.s001.zip › KA89/KA89A/KA890418.tif]

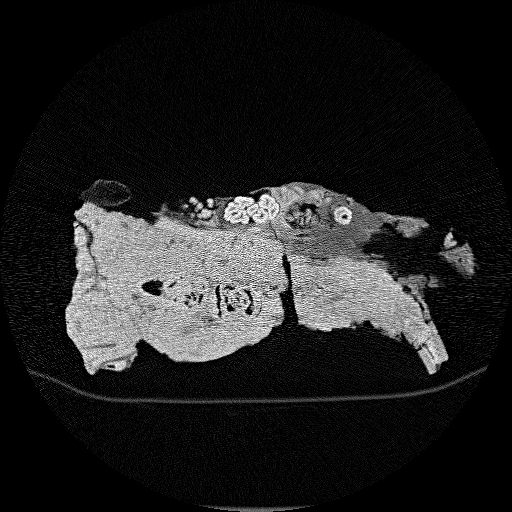

Supplement: S1 Dataset — (ZIP) [file pone.0139800.s001.zip › KA89/KA89A/KA890419.tif]

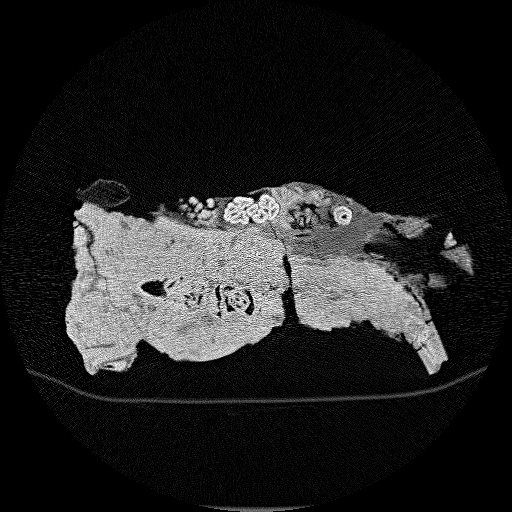

Supplement: S1 Dataset — (ZIP) [file pone.0139800.s001.zip › KA89/KA89A/KA890420.tif]

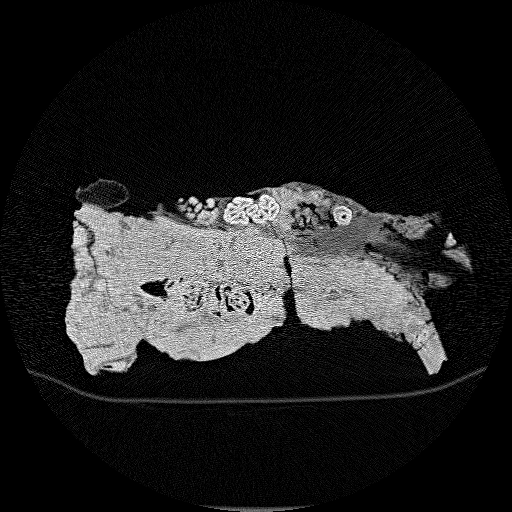

Supplement: S1 Dataset — (ZIP) [file pone.0139800.s001.zip › KA89/KA89A/KA890421.tif]

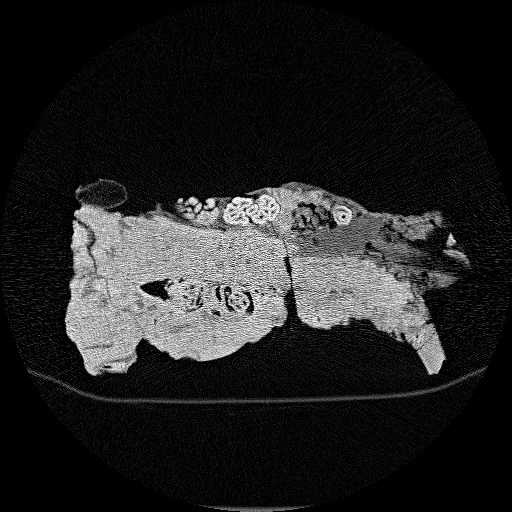

Supplement: S1 Dataset — (ZIP) [file pone.0139800.s001.zip › KA89/KA89A/KA890422.tif]

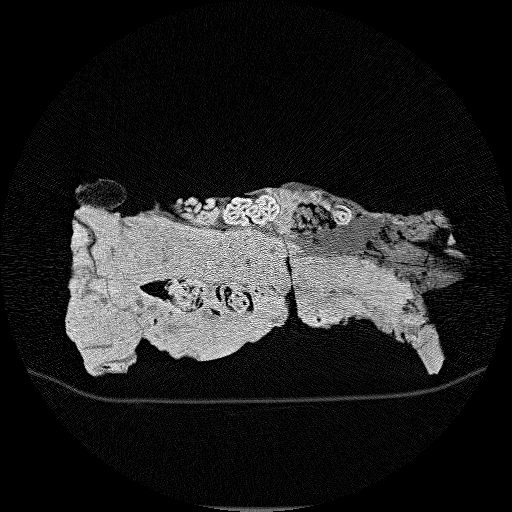

Supplement: S1 Dataset — (ZIP) [file pone.0139800.s001.zip › KA89/KA89A/KA890423.tif]

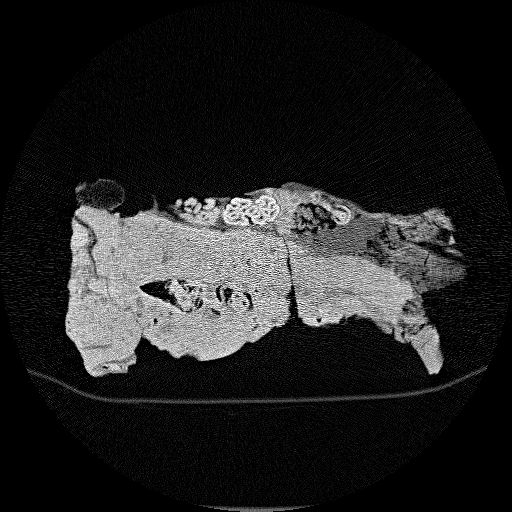

Supplement: S1 Dataset — (ZIP) [file pone.0139800.s001.zip › KA89/KA89A/KA890424.tif]

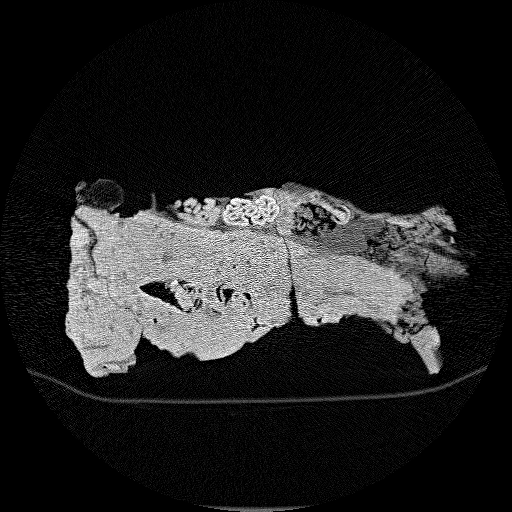

Supplement: S1 Dataset — (ZIP) [file pone.0139800.s001.zip › KA89/KA89A/KA890425.tif]

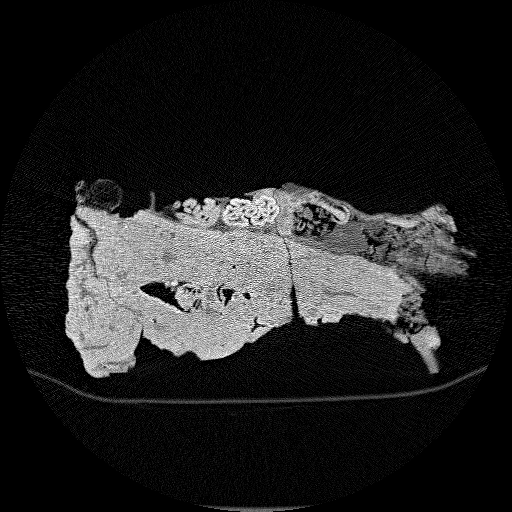

Supplement: S1 Dataset — (ZIP) [file pone.0139800.s001.zip › KA89/KA89A/KA890426.tif]

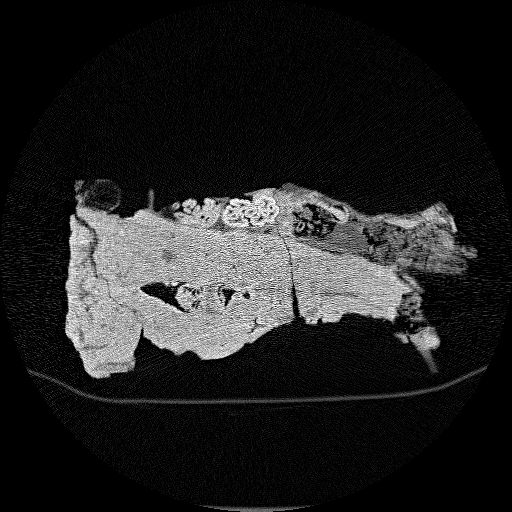

Supplement: S1 Dataset — (ZIP) [file pone.0139800.s001.zip › KA89/KA89A/KA890427.tif]

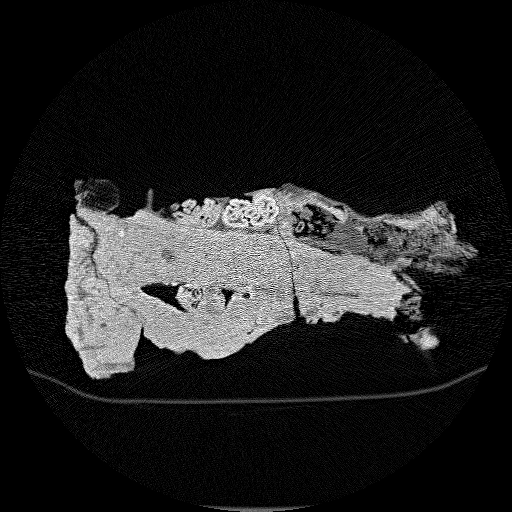

Supplement: S1 Dataset — (ZIP) [file pone.0139800.s001.zip › KA89/KA89A/KA890428.tif]

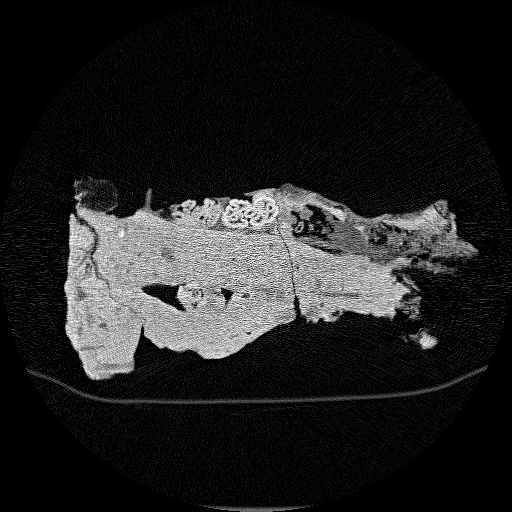

Supplement: S1 Dataset — (ZIP) [file pone.0139800.s001.zip › KA89/KA89A/KA890429.tif]

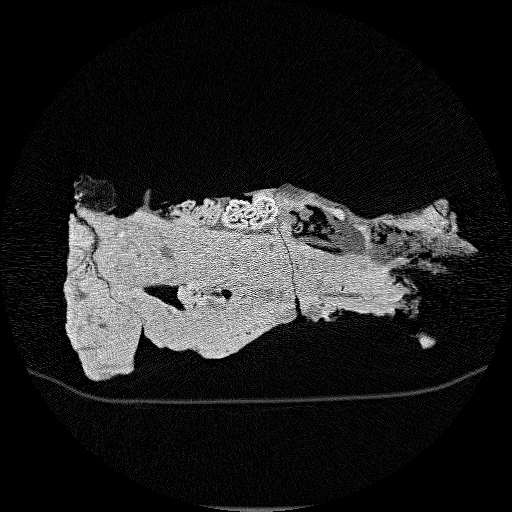

Supplement: S1 Dataset — (ZIP) [file pone.0139800.s001.zip › KA89/KA89A/KA890430.tif]

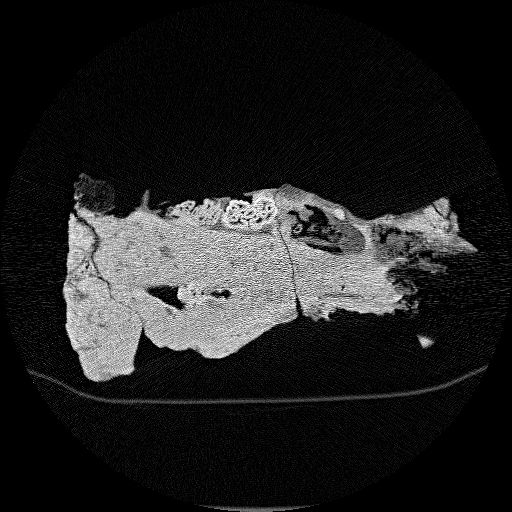

Supplement: S1 Dataset — (ZIP) [file pone.0139800.s001.zip › KA89/KA89A/KA890431.tif]

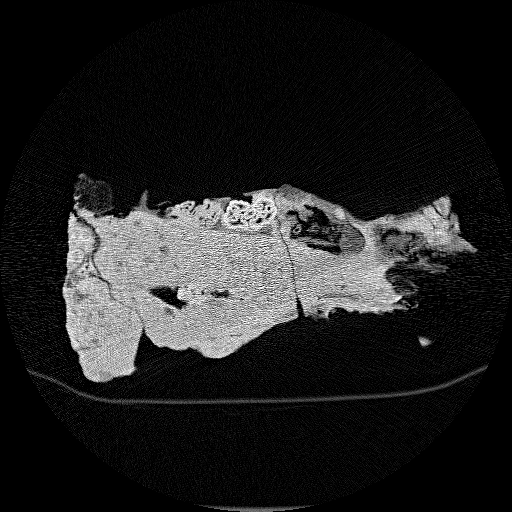

Supplement: S1 Dataset — (ZIP) [file pone.0139800.s001.zip › KA89/KA89A/KA890432.tif]

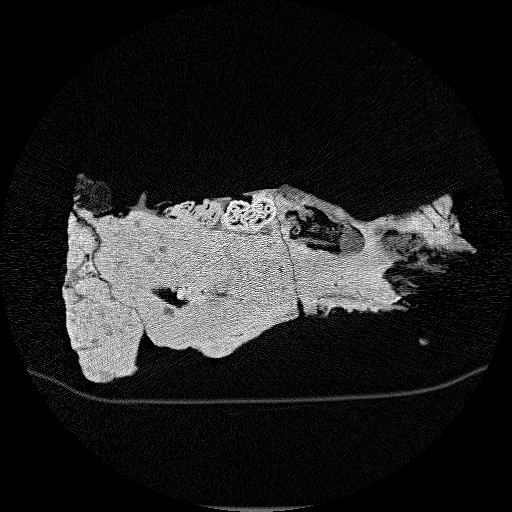

Supplement: S1 Dataset — (ZIP) [file pone.0139800.s001.zip › KA89/KA89A/KA890433.tif]

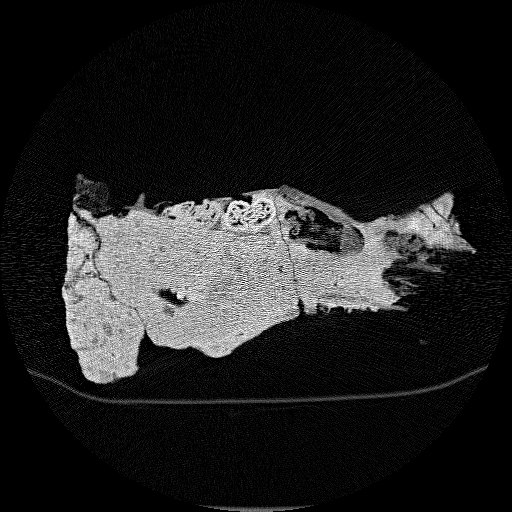

Supplement: S1 Dataset — (ZIP) [file pone.0139800.s001.zip › KA89/KA89A/KA890434.tif]

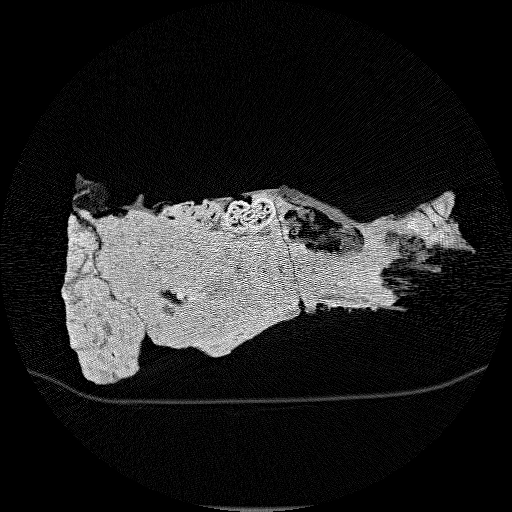

Supplement: S1 Dataset — (ZIP) [file pone.0139800.s001.zip › KA89/KA89A/KA890435.tif]

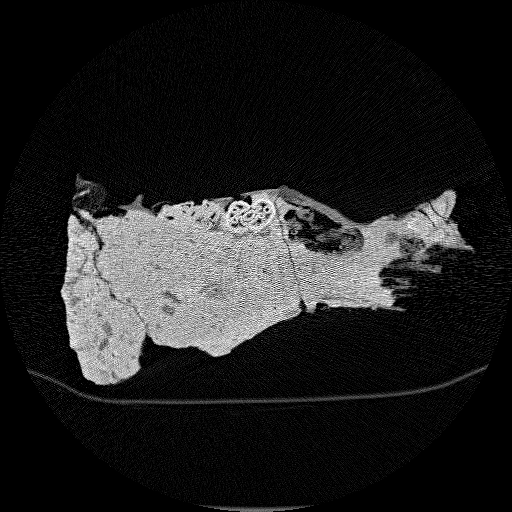

Supplement: S1 Dataset — (ZIP) [file pone.0139800.s001.zip › KA89/KA89A/KA890436.tif]

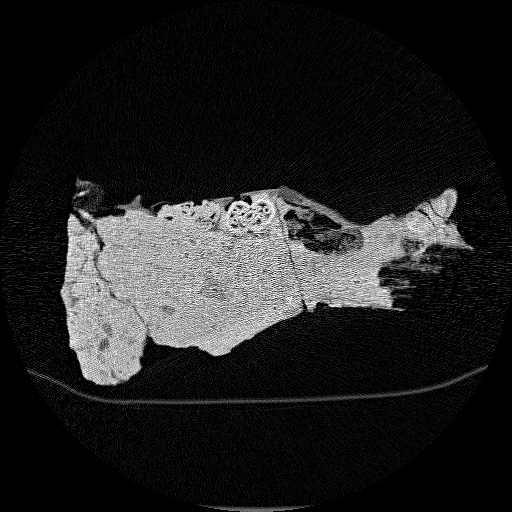

Supplement: S1 Dataset — (ZIP) [file pone.0139800.s001.zip › KA89/KA89A/KA890437.tif]

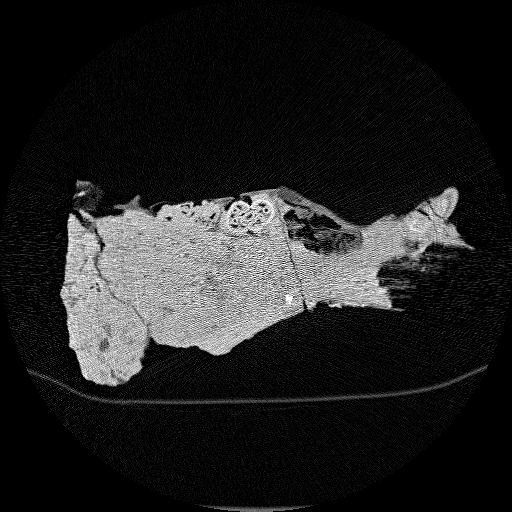

Supplement: S1 Dataset — (ZIP) [file pone.0139800.s001.zip › KA89/KA89A/KA890438.tif]

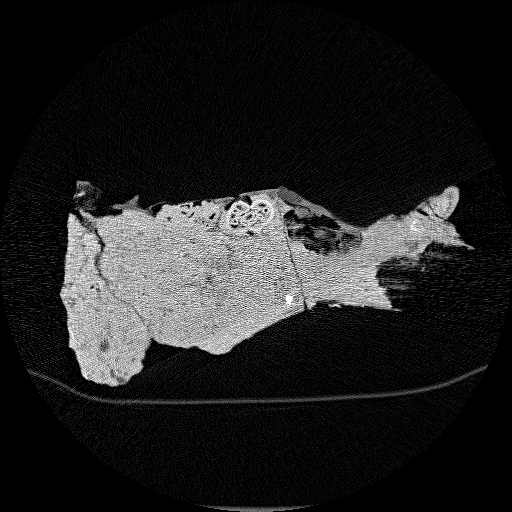

Supplement: S1 Dataset — (ZIP) [file pone.0139800.s001.zip › KA89/KA89A/KA890439.tif]

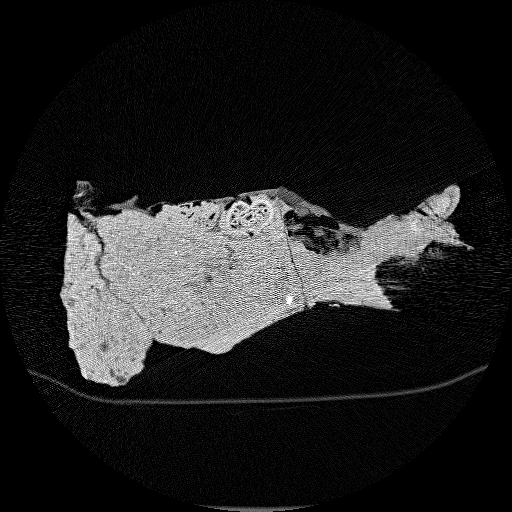

Supplement: S1 Dataset — (ZIP) [file pone.0139800.s001.zip › KA89/KA89A/KA890440.tif]

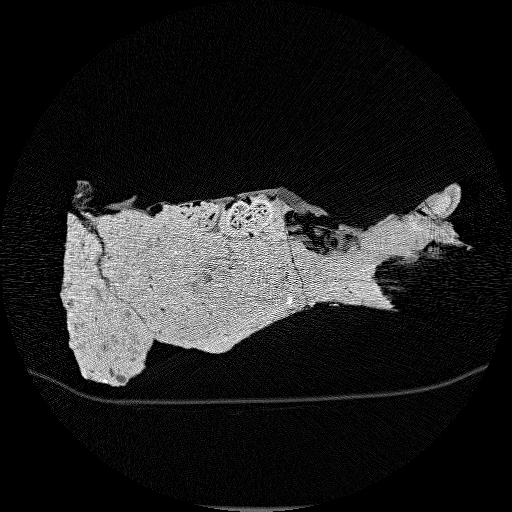

Supplement: S1 Dataset — (ZIP) [file pone.0139800.s001.zip › KA89/KA89A/KA890441.tif]

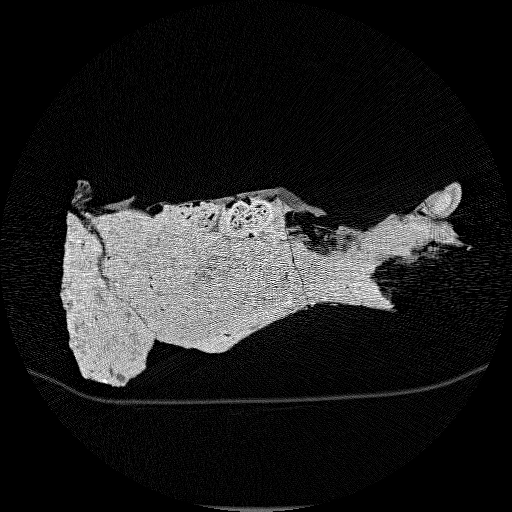

Supplement: S1 Dataset — (ZIP) [file pone.0139800.s001.zip › KA89/KA89A/KA890442.tif]

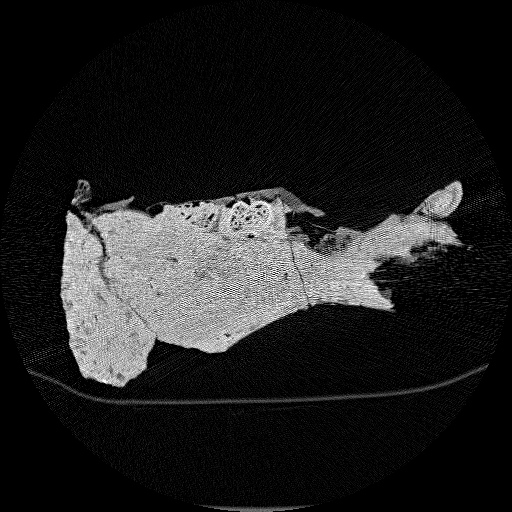

Supplement: S1 Dataset — (ZIP) [file pone.0139800.s001.zip › KA89/KA89A/KA890443.tif]

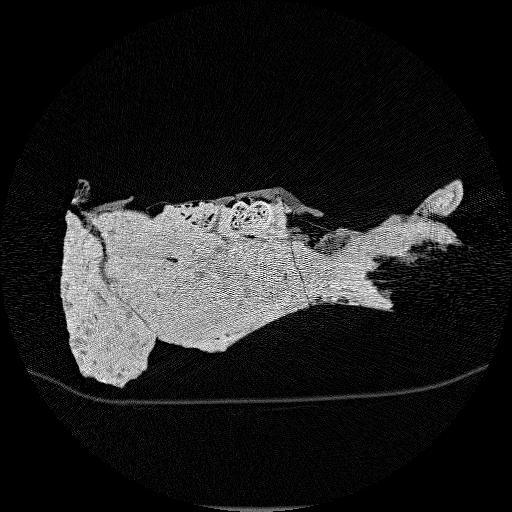

Supplement: S1 Dataset — (ZIP) [file pone.0139800.s001.zip › KA89/KA89A/KA890444.tif]

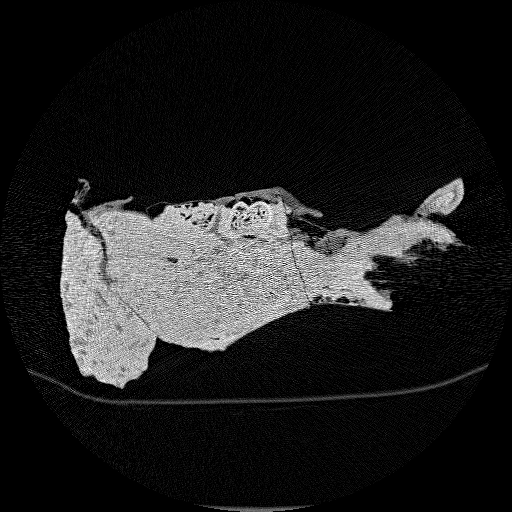

Supplement: S1 Dataset — (ZIP) [file pone.0139800.s001.zip › KA89/KA89A/KA890445.tif]

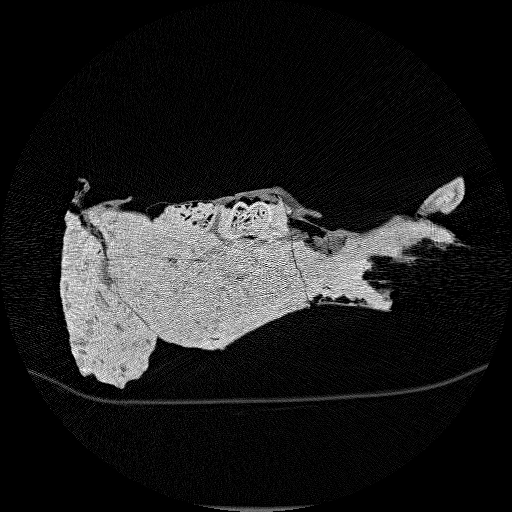

Supplement: S1 Dataset — (ZIP) [file pone.0139800.s001.zip › KA89/KA89A/KA890446.tif]

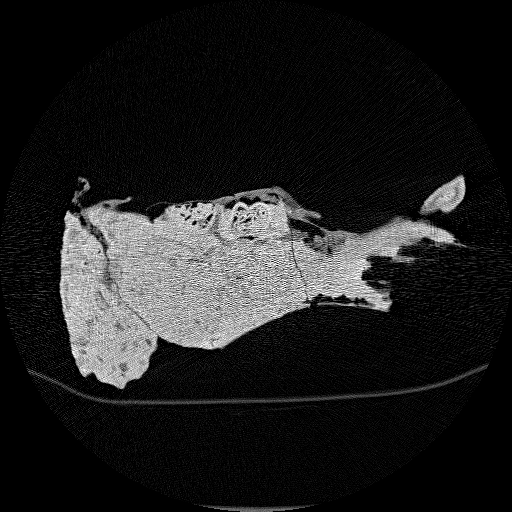

Supplement: S1 Dataset — (ZIP) [file pone.0139800.s001.zip › KA89/KA89A/KA890447.tif]

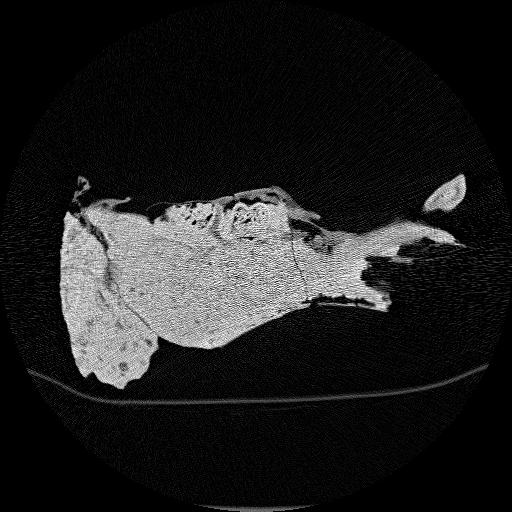

Supplement: S1 Dataset — (ZIP) [file pone.0139800.s001.zip › KA89/KA89A/KA890448.tif]

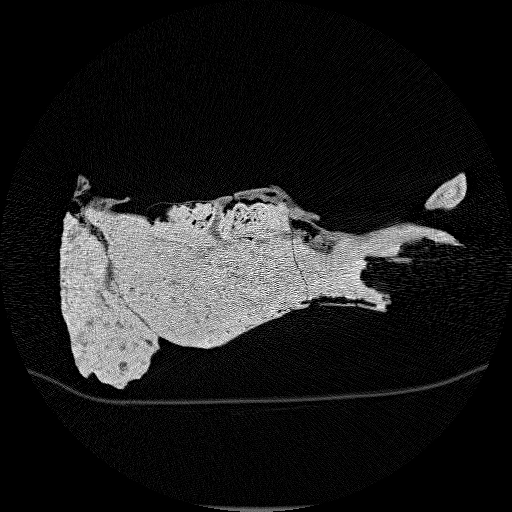

Supplement: S1 Dataset — (ZIP) [file pone.0139800.s001.zip › KA89/KA89A/KA890449.tif]

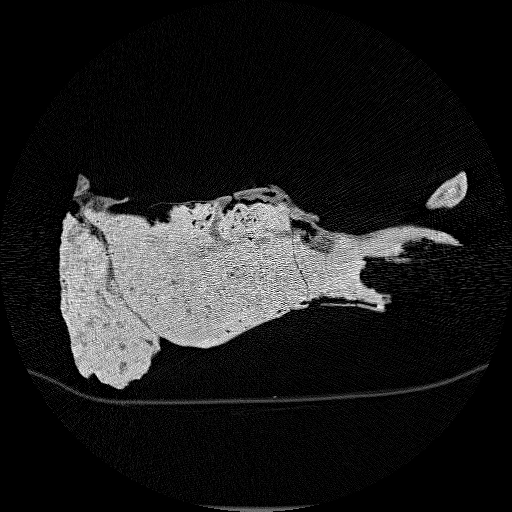

Supplement: S1 Dataset — (ZIP) [file pone.0139800.s001.zip › KA89/KA89A/KA890450.tif]

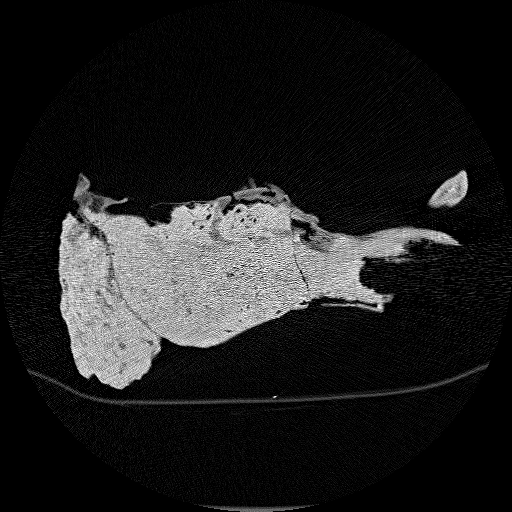

Supplement: S1 Dataset — (ZIP) [file pone.0139800.s001.zip › KA89/KA89A/KA890451.tif]

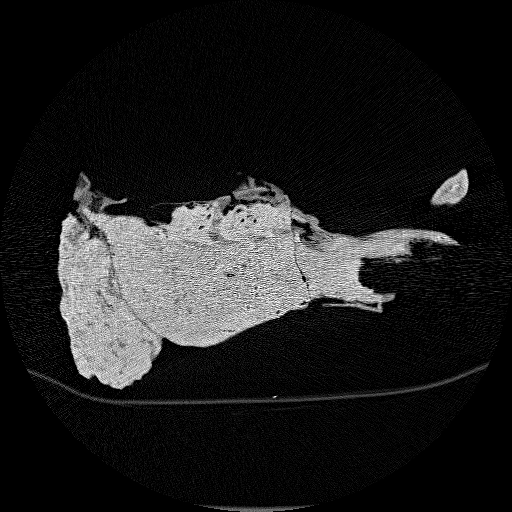

Supplement: S1 Dataset — (ZIP) [file pone.0139800.s001.zip › KA89/KA89A/KA890452.tif]

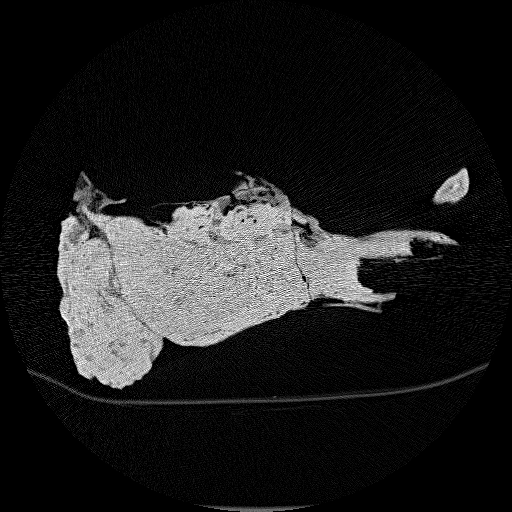

Supplement: S1 Dataset — (ZIP) [file pone.0139800.s001.zip › KA89/KA89A/KA890453.tif]

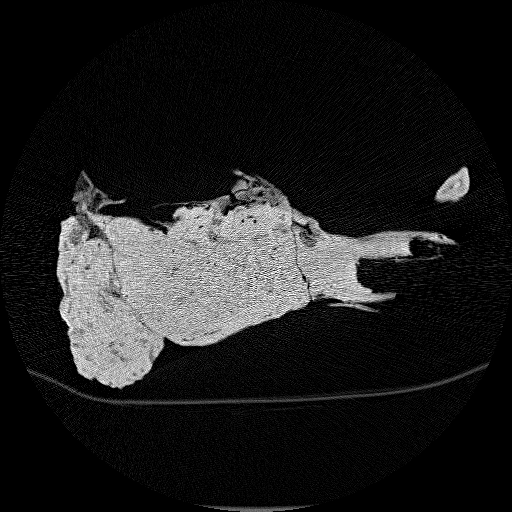

Supplement: S1 Dataset — (ZIP) [file pone.0139800.s001.zip › KA89/KA89A/KA890454.tif]

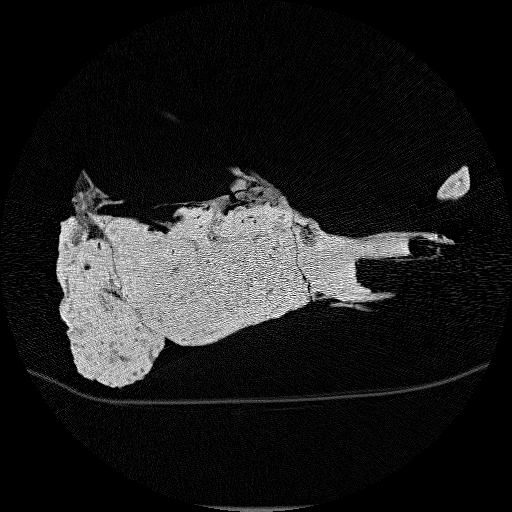

Supplement: S1 Dataset — (ZIP) [file pone.0139800.s001.zip › KA89/KA89A/KA890455.tif]

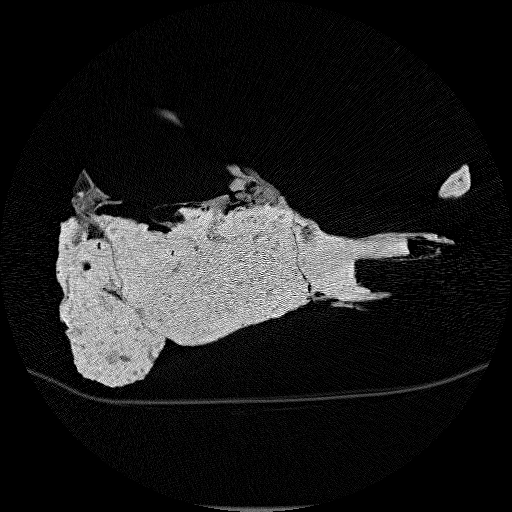

Supplement: S1 Dataset — (ZIP) [file pone.0139800.s001.zip › KA89/KA89A/KA890456.tif]

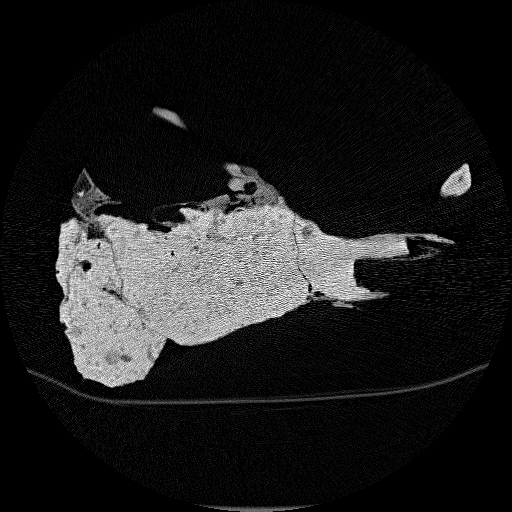

Supplement: S1 Dataset — (ZIP) [file pone.0139800.s001.zip › KA89/KA89A/KA890457.tif]

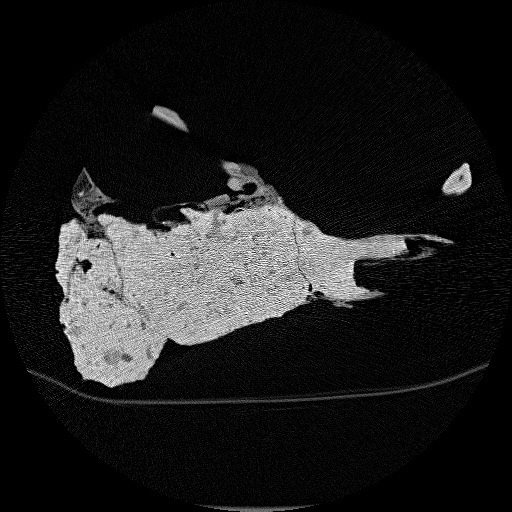

Supplement: S1 Dataset — (ZIP) [file pone.0139800.s001.zip › KA89/KA89A/KA890458.tif]

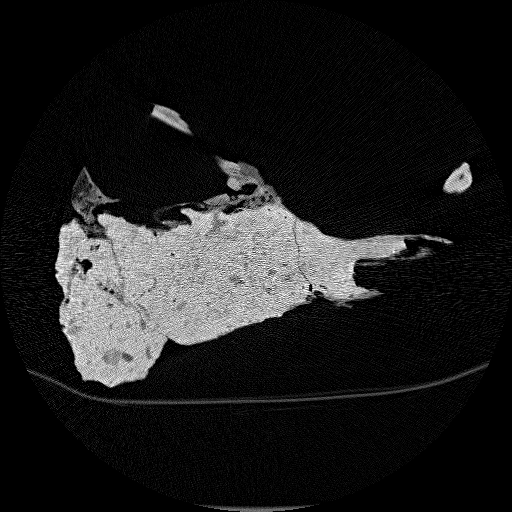

Supplement: S1 Dataset — (ZIP) [file pone.0139800.s001.zip › KA89/KA89A/KA890459.tif]

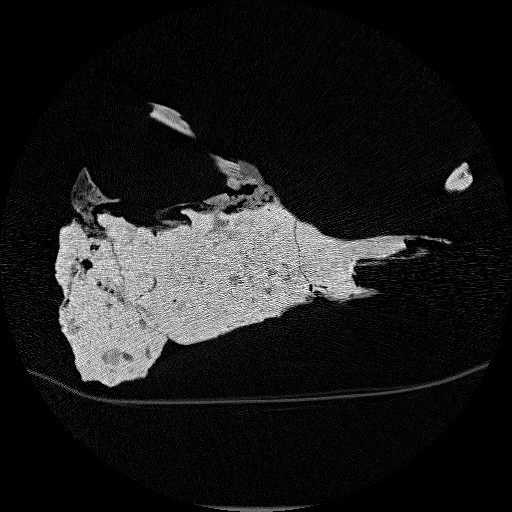

Supplement: S1 Dataset — (ZIP) [file pone.0139800.s001.zip › KA89/KA89A/KA890460.tif]

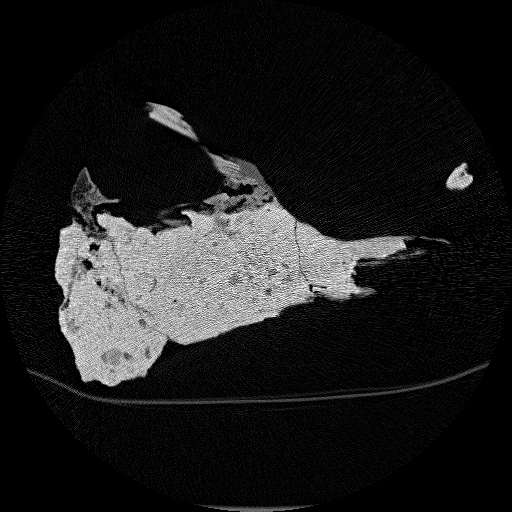

Supplement: S1 Dataset — (ZIP) [file pone.0139800.s001.zip › KA89/KA89A/KA890461.tif]

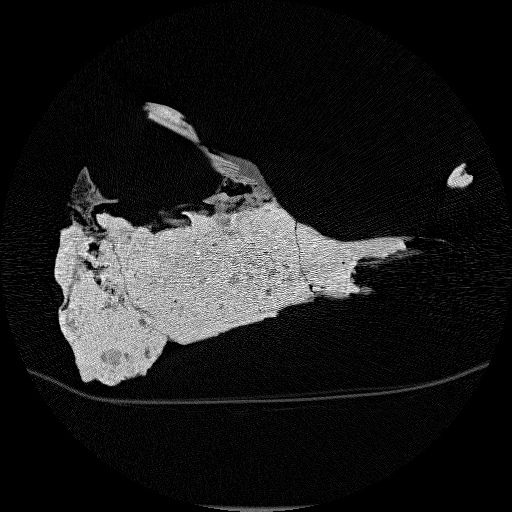

Supplement: S1 Dataset — (ZIP) [file pone.0139800.s001.zip › KA89/KA89A/KA890462.tif]

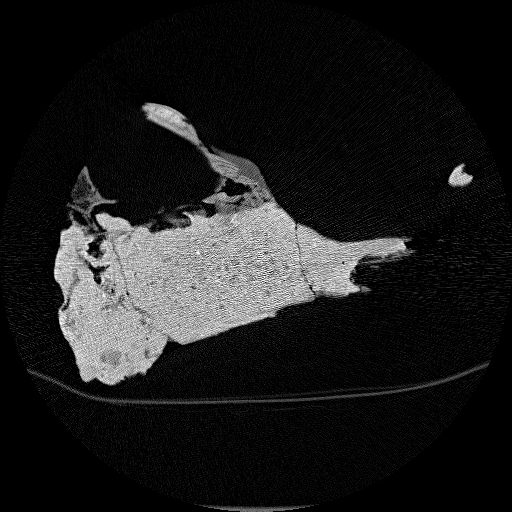

Supplement: S1 Dataset — (ZIP) [file pone.0139800.s001.zip › KA89/KA89A/KA890463.tif]

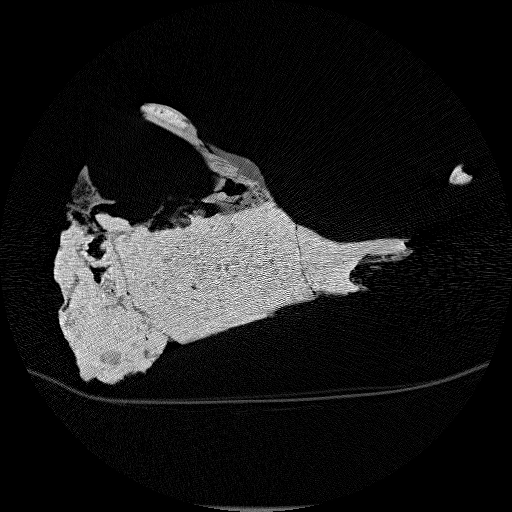

Supplement: S1 Dataset — (ZIP) [file pone.0139800.s001.zip › KA89/KA89A/KA890464.tif]

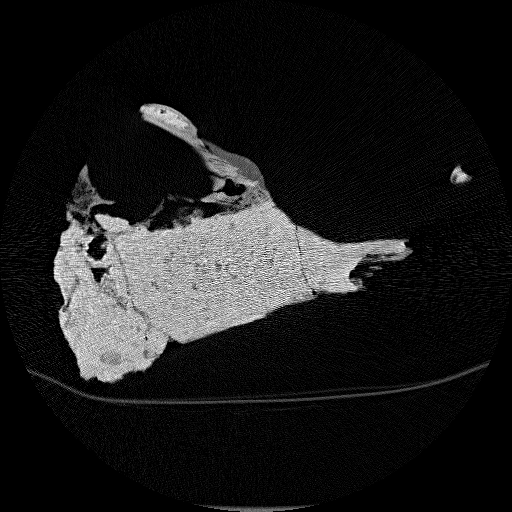

Supplement: S1 Dataset — (ZIP) [file pone.0139800.s001.zip › KA89/KA89A/KA890465.tif]

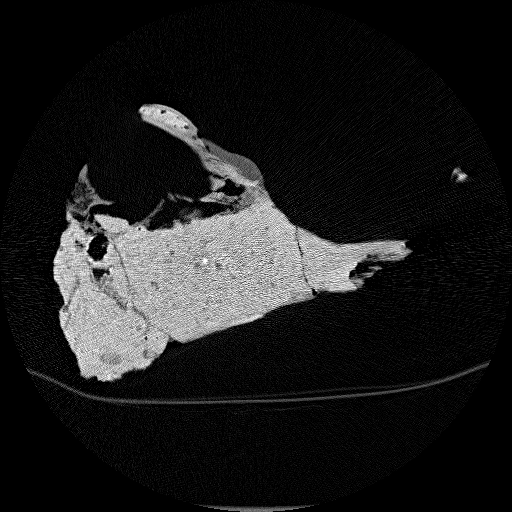

Supplement: S1 Dataset — (ZIP) [file pone.0139800.s001.zip › KA89/KA89A/KA890466.tif]

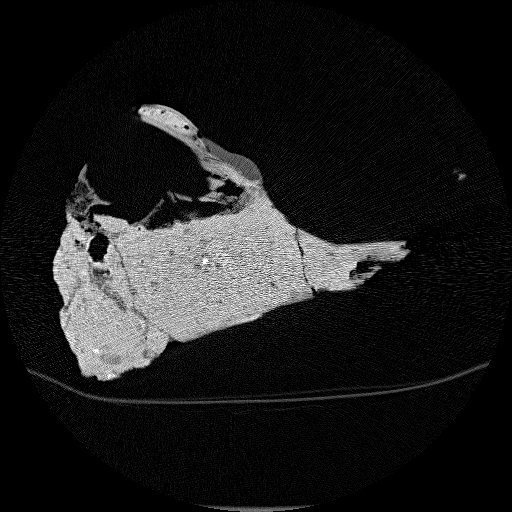

Supplement: S1 Dataset — (ZIP) [file pone.0139800.s001.zip › KA89/KA89A/KA890467.tif]

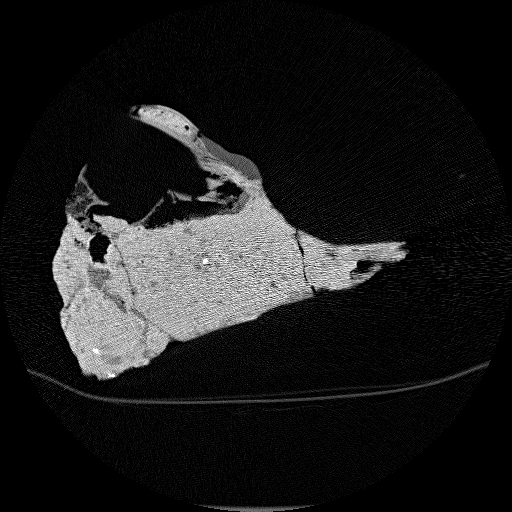

Supplement: S1 Dataset — (ZIP) [file pone.0139800.s001.zip › KA89/KA89A/KA890468.tif]

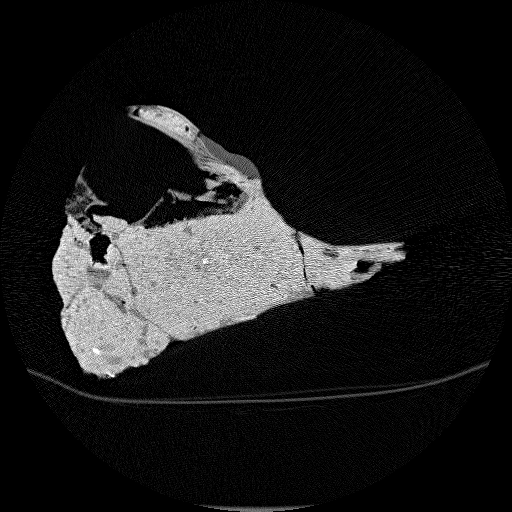

Supplement: S1 Dataset — (ZIP) [file pone.0139800.s001.zip › KA89/KA89A/KA890469.tif]

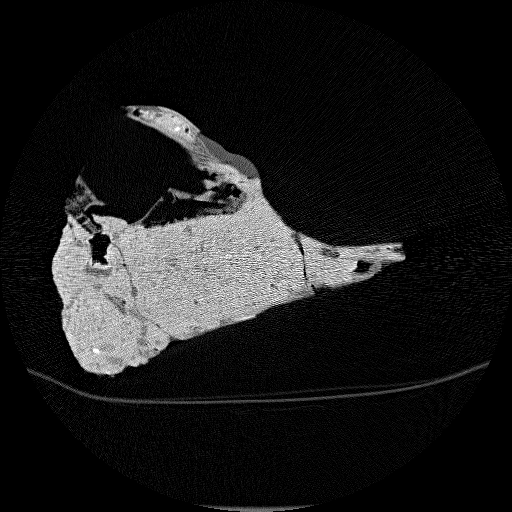

Supplement: S1 Dataset — (ZIP) [file pone.0139800.s001.zip › KA89/KA89A/KA890470.tif]

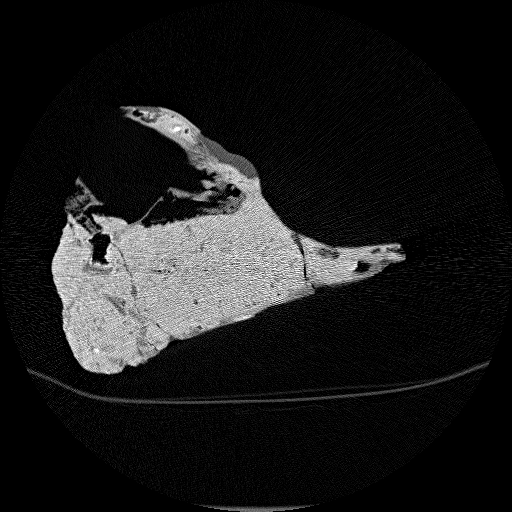

Supplement: S1 Dataset — (ZIP) [file pone.0139800.s001.zip › KA89/KA89A/KA890471.tif]

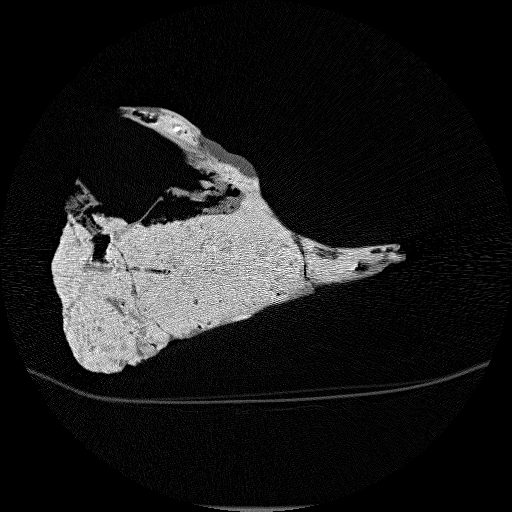

Supplement: S1 Dataset — (ZIP) [file pone.0139800.s001.zip › KA89/KA89A/KA890472.tif]

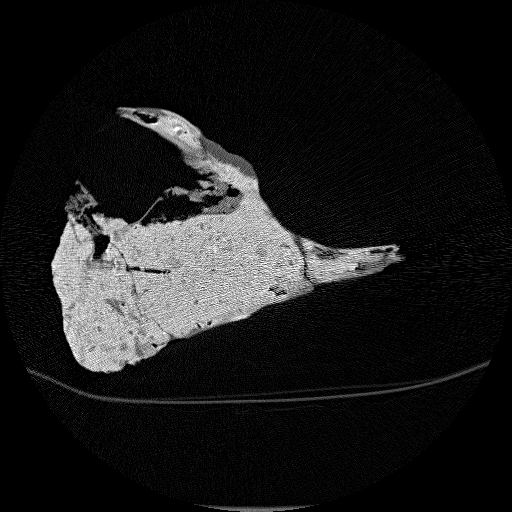

Supplement: S1 Dataset — (ZIP) [file pone.0139800.s001.zip › KA89/KA89A/KA890473.tif]

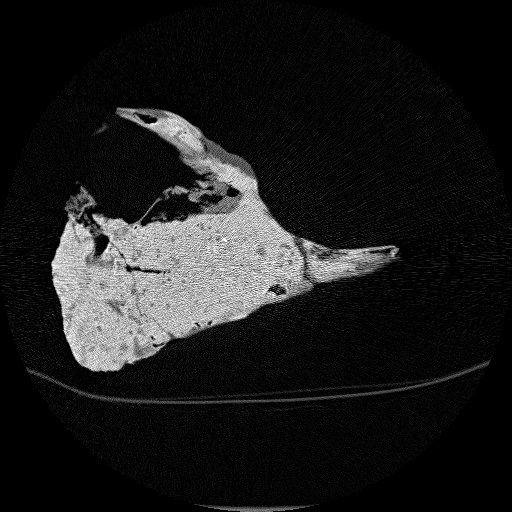

Supplement: S1 Dataset — (ZIP) [file pone.0139800.s001.zip › KA89/KA89A/KA890474.tif]

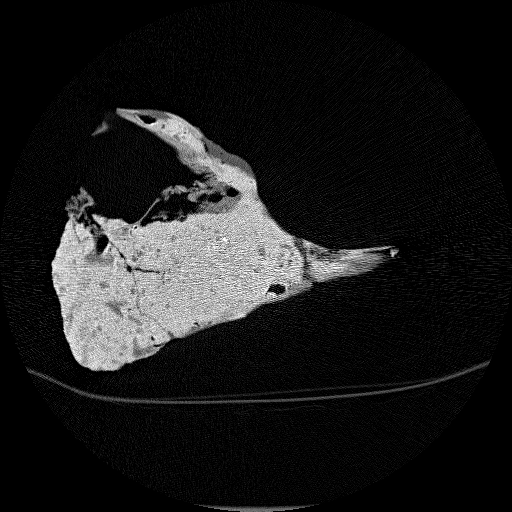

Supplement: S1 Dataset — (ZIP) [file pone.0139800.s001.zip › KA89/KA89A/KA890475.tif]

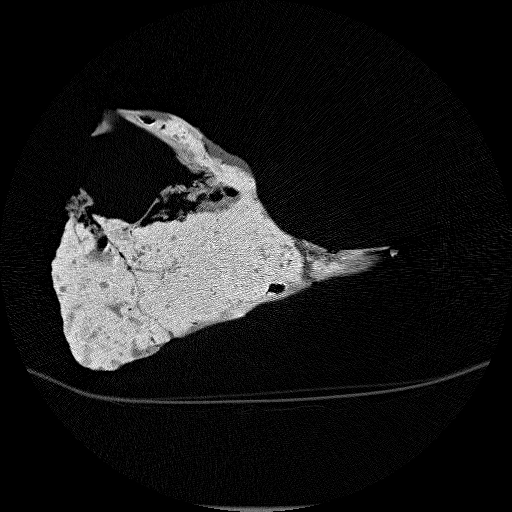

Supplement: S1 Dataset — (ZIP) [file pone.0139800.s001.zip › KA89/KA89A/KA890476.tif]

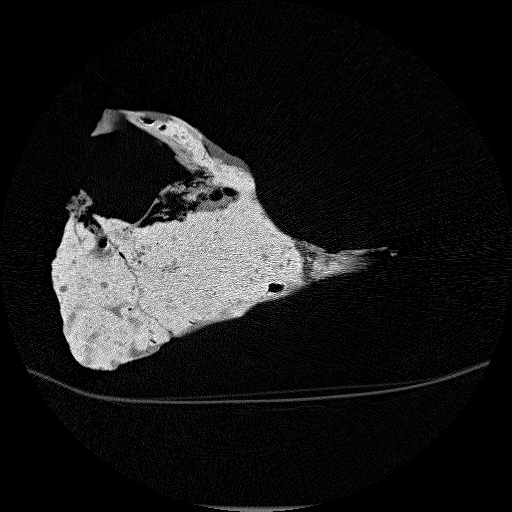

Supplement: S1 Dataset — (ZIP) [file pone.0139800.s001.zip › KA89/KA89A/KA890477.tif]

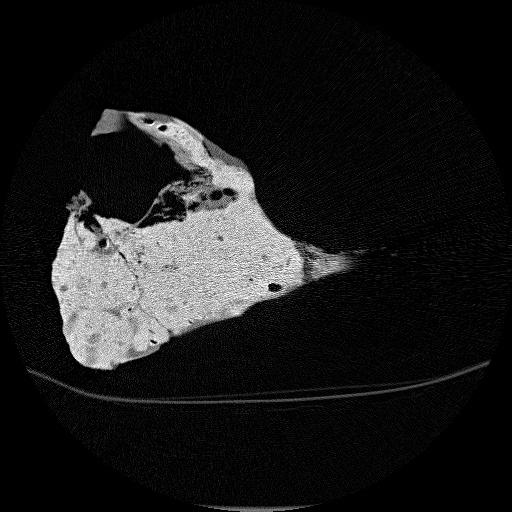

Supplement: S1 Dataset — (ZIP) [file pone.0139800.s001.zip › KA89/KA89A/KA890478.tif]

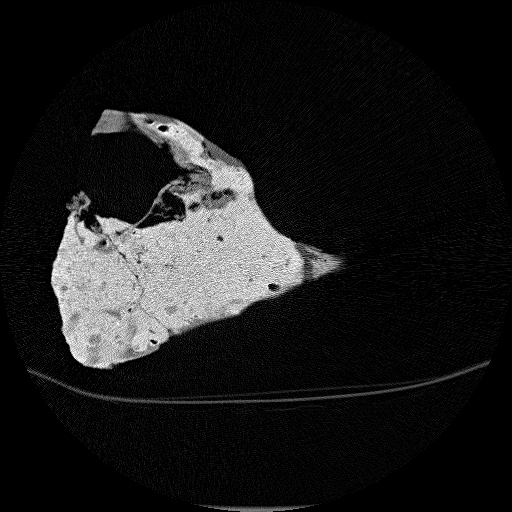

Supplement: S1 Dataset — (ZIP) [file pone.0139800.s001.zip › KA89/KA89A/KA890479.tif]

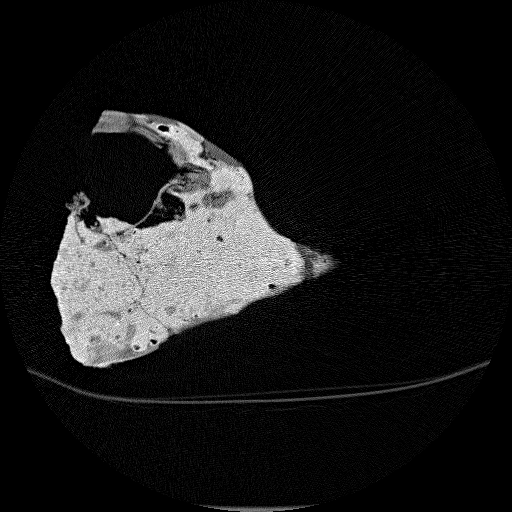

Supplement: S1 Dataset — (ZIP) [file pone.0139800.s001.zip › KA89/KA89A/KA890480.tif]

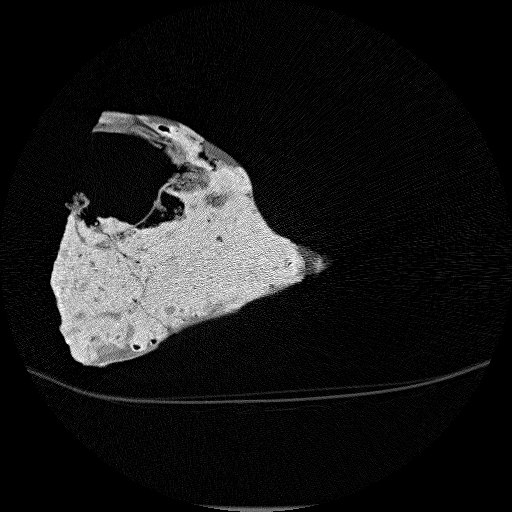

Supplement: S1 Dataset — (ZIP) [file pone.0139800.s001.zip › KA89/KA89A/KA890481.tif]

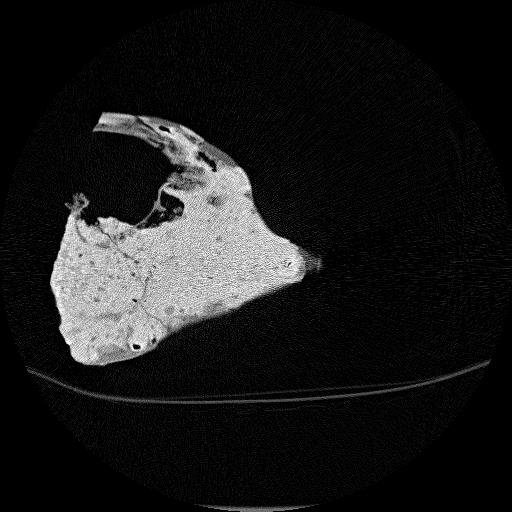

Supplement: S1 Dataset — (ZIP) [file pone.0139800.s001.zip › KA89/KA89A/KA890482.tif]

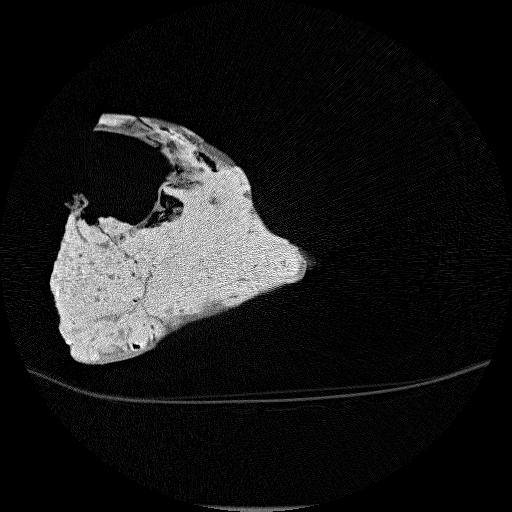

Supplement: S1 Dataset — (ZIP) [file pone.0139800.s001.zip › KA89/KA89A/KA890483.tif]

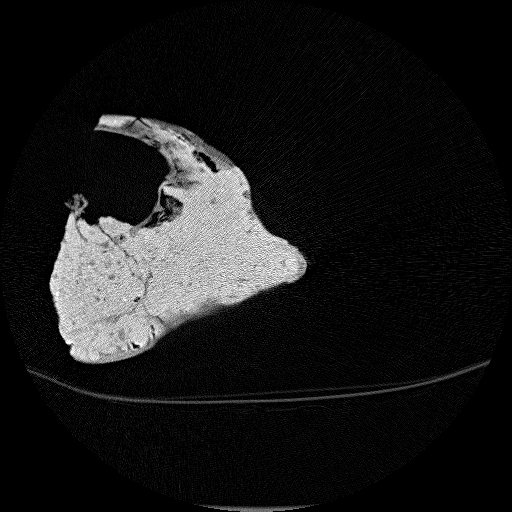

Supplement: S1 Dataset — (ZIP) [file pone.0139800.s001.zip › KA89/KA89A/KA890484.tif]
