# Supplementary figures and images for: Surface Model and Tomographic Archive of Fossil Primate and Other Mammal Holotype and Paratype Specimens of the Ditsong National Museum of Natural History, Pretoria, South Africa (part 1 of 2)
Source: PLoS One. 2015 Oct 6;10(10):e0139800. doi: 10.1371/journal.pone.0139800 (PMC4595468; doi:10.1371/journal.pone.0139800)

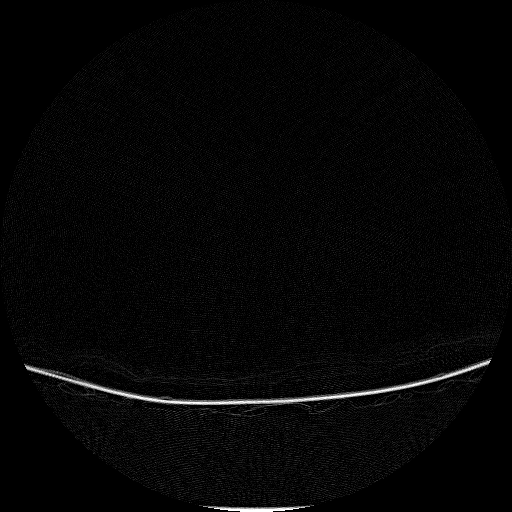

Supplement: S1 Dataset — (ZIP) [file pone.0139800.s001.zip › KA89/KA89A/KA890285.tif]

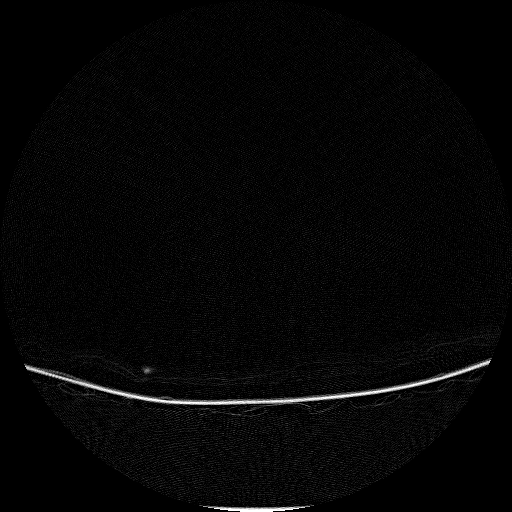

Supplement: S1 Dataset — (ZIP) [file pone.0139800.s001.zip › KA89/KA89A/KA890286.tif]

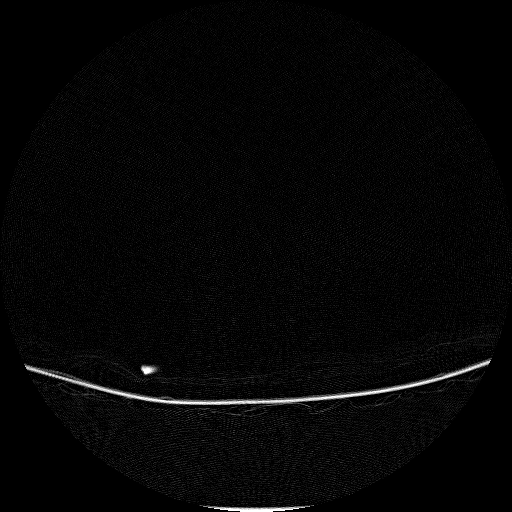

Supplement: S1 Dataset — (ZIP) [file pone.0139800.s001.zip › KA89/KA89A/KA890287.tif]

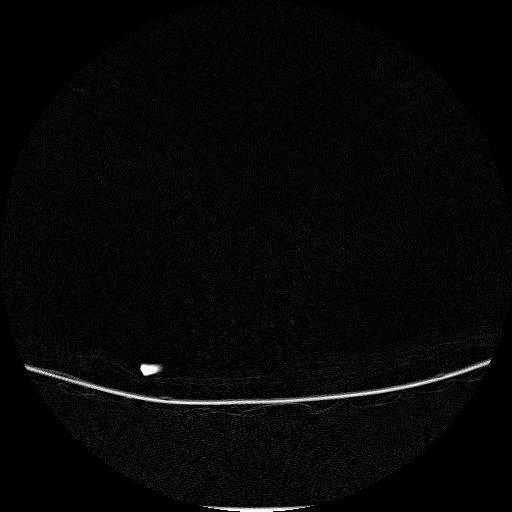

Supplement: S1 Dataset — (ZIP) [file pone.0139800.s001.zip › KA89/KA89A/KA890288.tif]

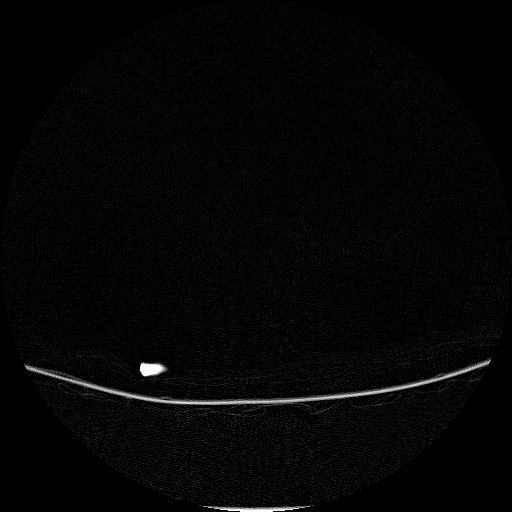

Supplement: S1 Dataset — (ZIP) [file pone.0139800.s001.zip › KA89/KA89A/KA890289.tif]

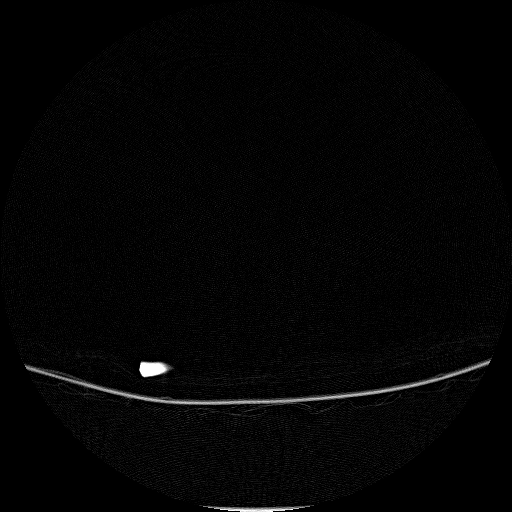

Supplement: S1 Dataset — (ZIP) [file pone.0139800.s001.zip › KA89/KA89A/KA890290.tif]

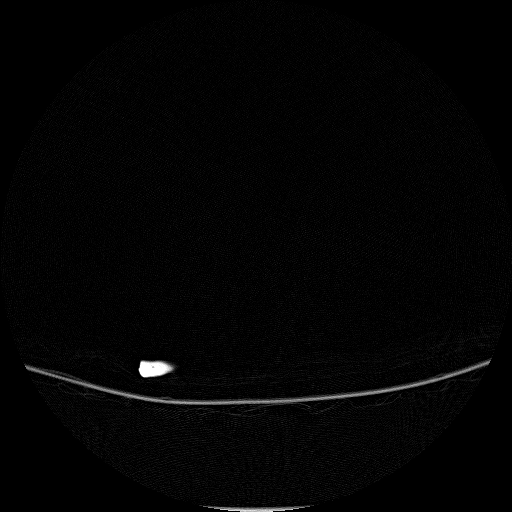

Supplement: S1 Dataset — (ZIP) [file pone.0139800.s001.zip › KA89/KA89A/KA890291.tif]

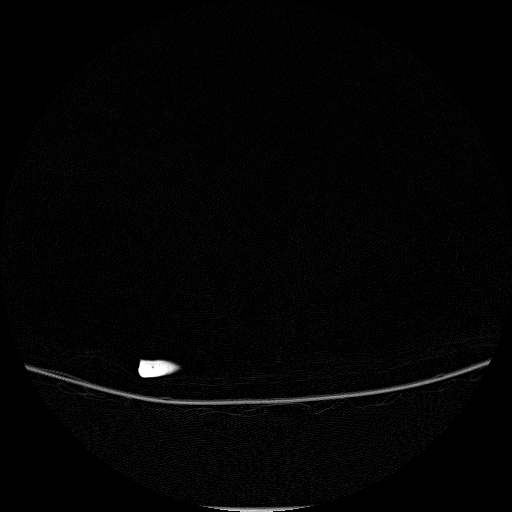

Supplement: S1 Dataset — (ZIP) [file pone.0139800.s001.zip › KA89/KA89A/KA890292.tif]

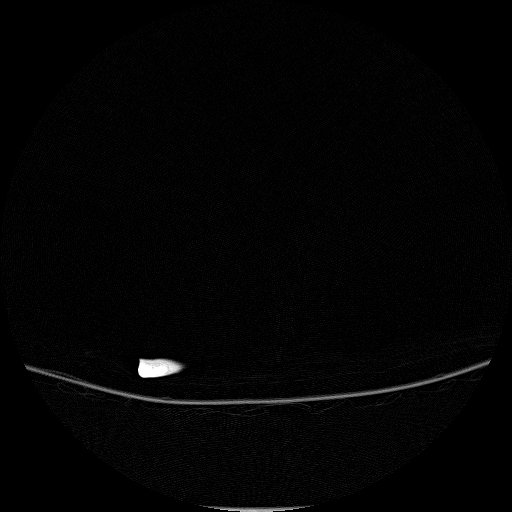

Supplement: S1 Dataset — (ZIP) [file pone.0139800.s001.zip › KA89/KA89A/KA890293.tif]

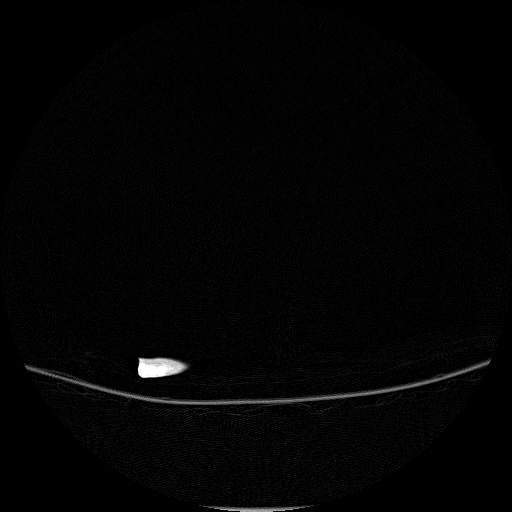

Supplement: S1 Dataset — (ZIP) [file pone.0139800.s001.zip › KA89/KA89A/KA890294.tif]

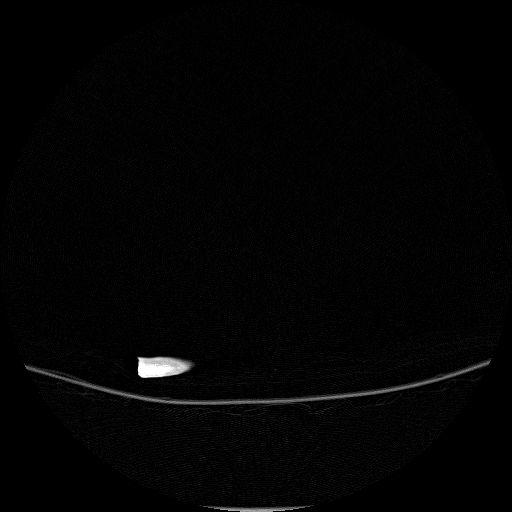

Supplement: S1 Dataset — (ZIP) [file pone.0139800.s001.zip › KA89/KA89A/KA890295.tif]

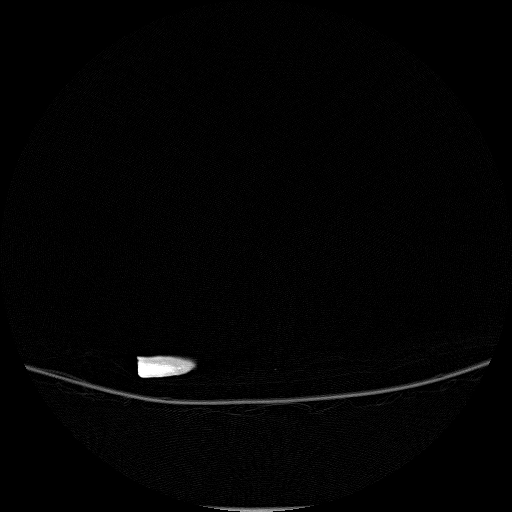

Supplement: S1 Dataset — (ZIP) [file pone.0139800.s001.zip › KA89/KA89A/KA890296.tif]

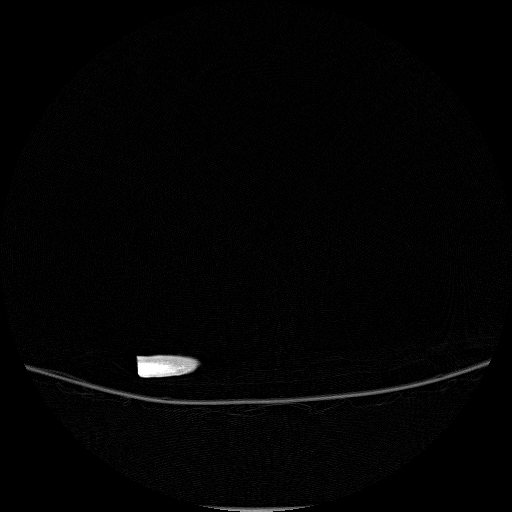

Supplement: S1 Dataset — (ZIP) [file pone.0139800.s001.zip › KA89/KA89A/KA890297.tif]

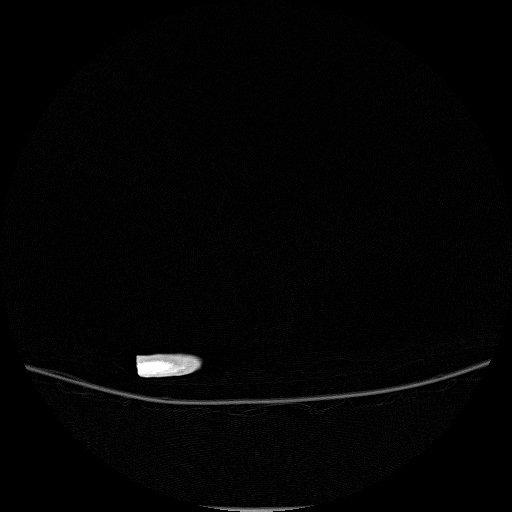

Supplement: S1 Dataset — (ZIP) [file pone.0139800.s001.zip › KA89/KA89A/KA890298.tif]

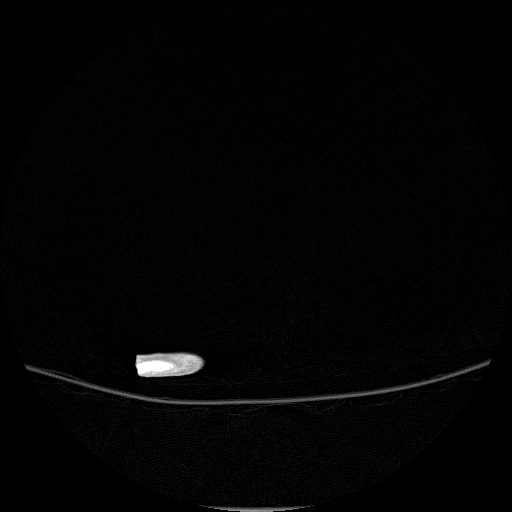

Supplement: S1 Dataset — (ZIP) [file pone.0139800.s001.zip › KA89/KA89A/KA890299.tif]

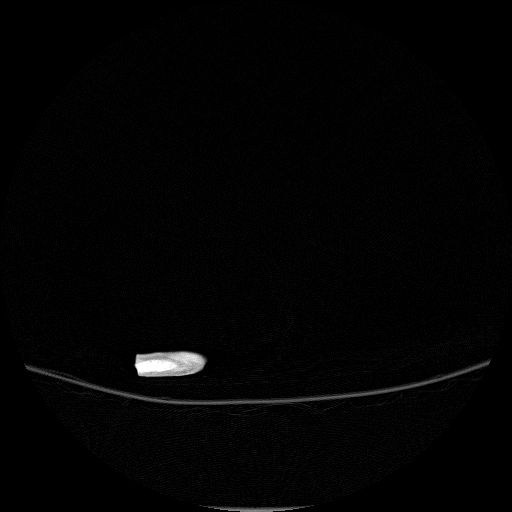

Supplement: S1 Dataset — (ZIP) [file pone.0139800.s001.zip › KA89/KA89A/KA890300.tif]

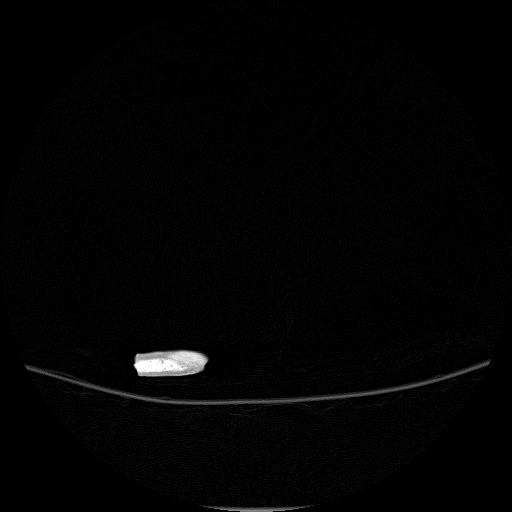

Supplement: S1 Dataset — (ZIP) [file pone.0139800.s001.zip › KA89/KA89A/KA890301.tif]

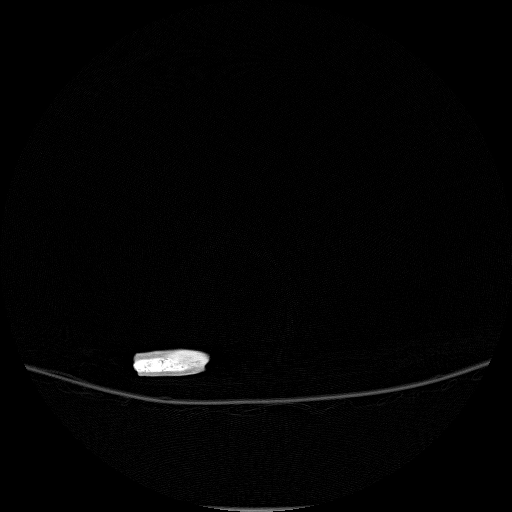

Supplement: S1 Dataset — (ZIP) [file pone.0139800.s001.zip › KA89/KA89A/KA890302.tif]

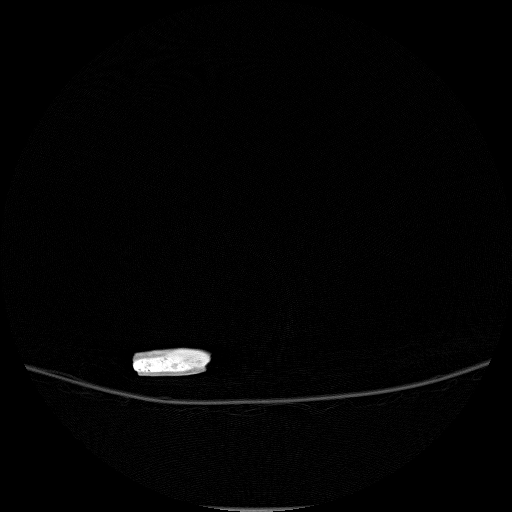

Supplement: S1 Dataset — (ZIP) [file pone.0139800.s001.zip › KA89/KA89A/KA890303.tif]

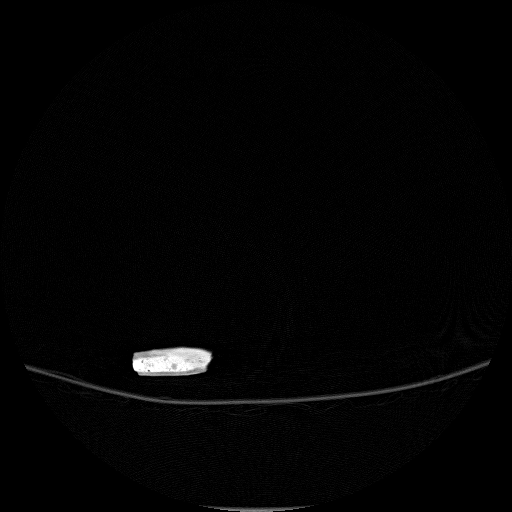

Supplement: S1 Dataset — (ZIP) [file pone.0139800.s001.zip › KA89/KA89A/KA890304.tif]

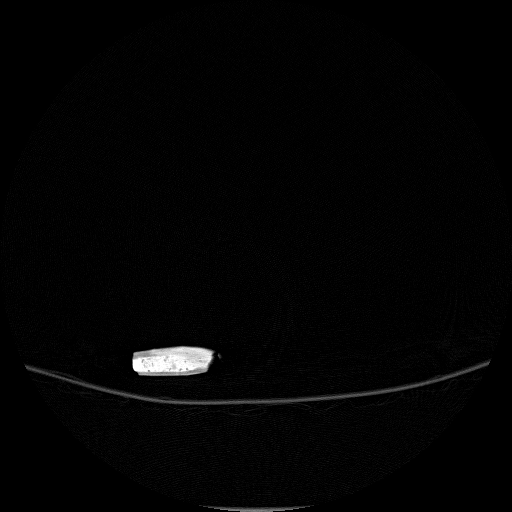

Supplement: S1 Dataset — (ZIP) [file pone.0139800.s001.zip › KA89/KA89A/KA890305.tif]

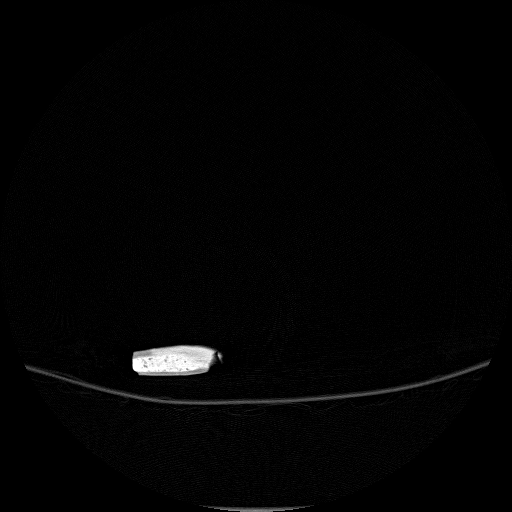

Supplement: S1 Dataset — (ZIP) [file pone.0139800.s001.zip › KA89/KA89A/KA890306.tif]

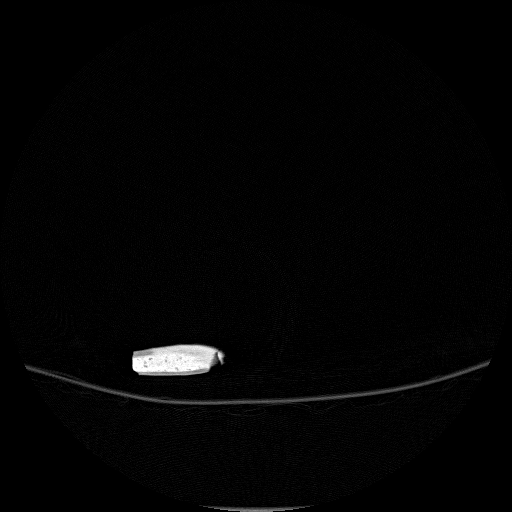

Supplement: S1 Dataset — (ZIP) [file pone.0139800.s001.zip › KA89/KA89A/KA890307.tif]

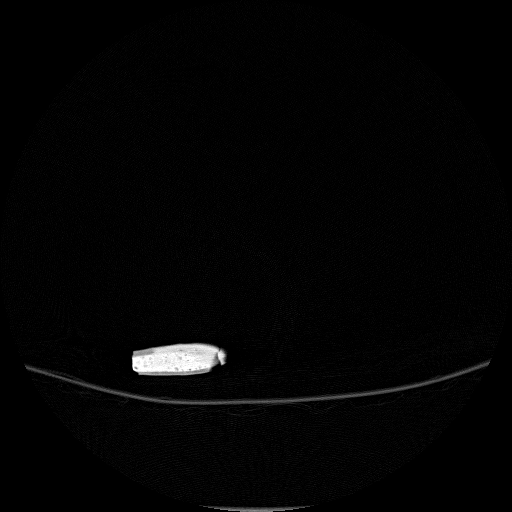

Supplement: S1 Dataset — (ZIP) [file pone.0139800.s001.zip › KA89/KA89A/KA890308.tif]

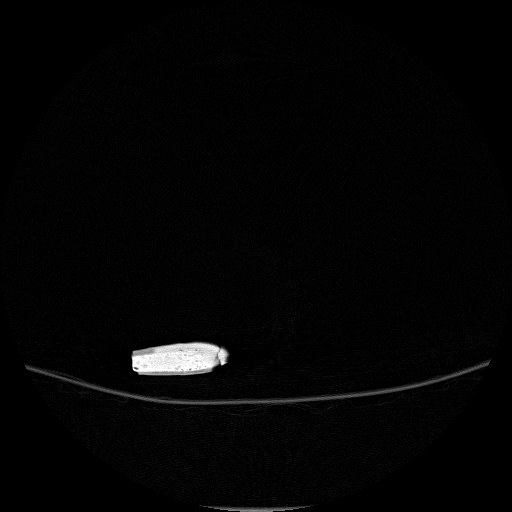

Supplement: S1 Dataset — (ZIP) [file pone.0139800.s001.zip › KA89/KA89A/KA890309.tif]

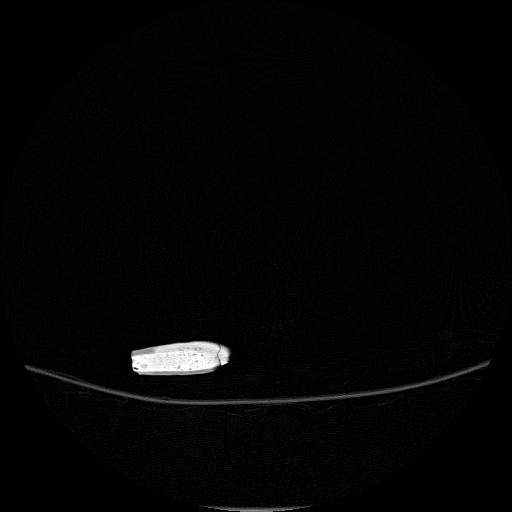

Supplement: S1 Dataset — (ZIP) [file pone.0139800.s001.zip › KA89/KA89A/KA890310.tif]

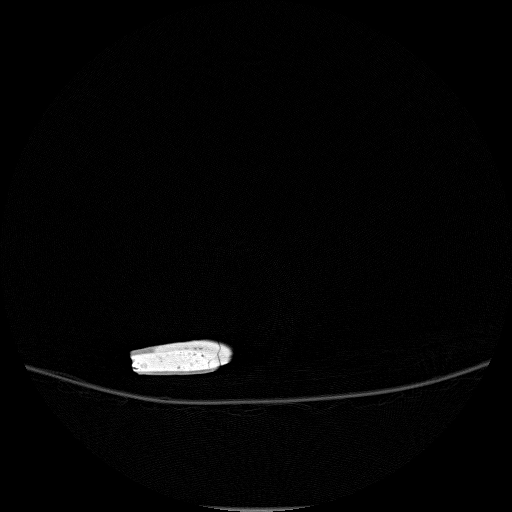

Supplement: S1 Dataset — (ZIP) [file pone.0139800.s001.zip › KA89/KA89A/KA890311.tif]

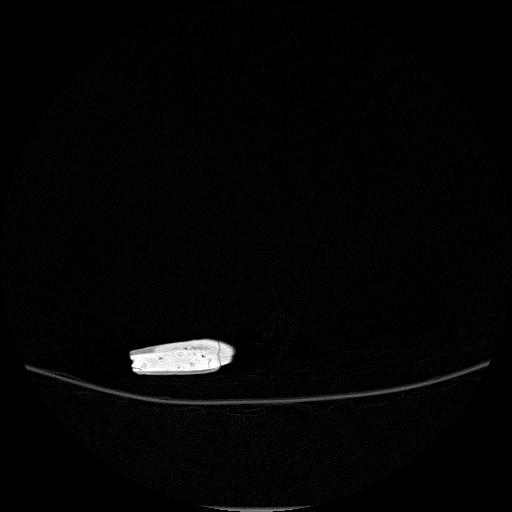

Supplement: S1 Dataset — (ZIP) [file pone.0139800.s001.zip › KA89/KA89A/KA890312.tif]

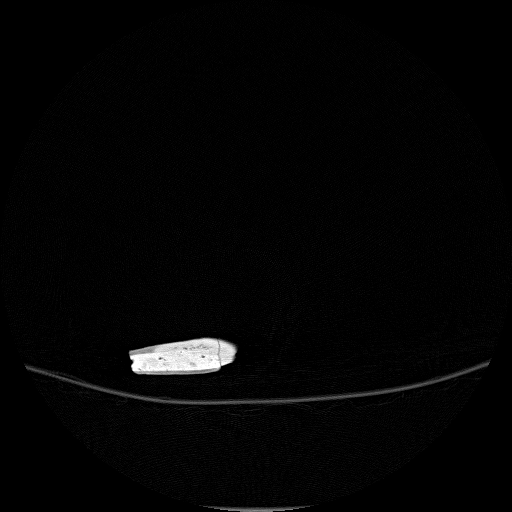

Supplement: S1 Dataset — (ZIP) [file pone.0139800.s001.zip › KA89/KA89A/KA890313.tif]

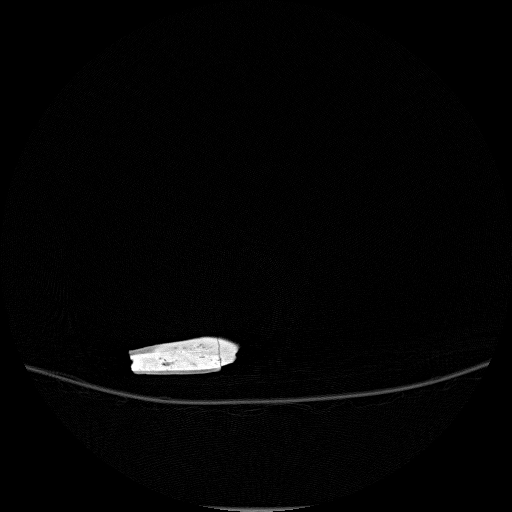

Supplement: S1 Dataset — (ZIP) [file pone.0139800.s001.zip › KA89/KA89A/KA890314.tif]

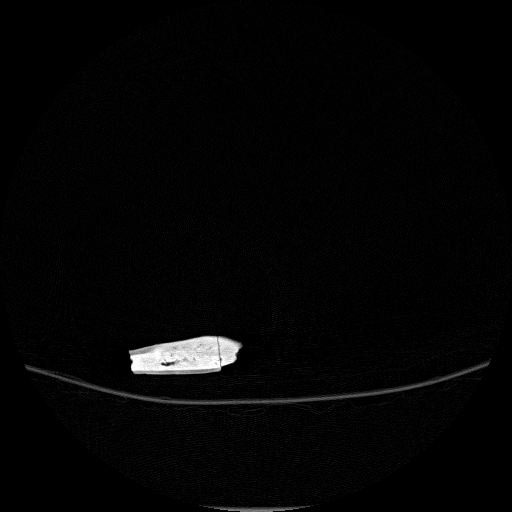

Supplement: S1 Dataset — (ZIP) [file pone.0139800.s001.zip › KA89/KA89A/KA890315.tif]

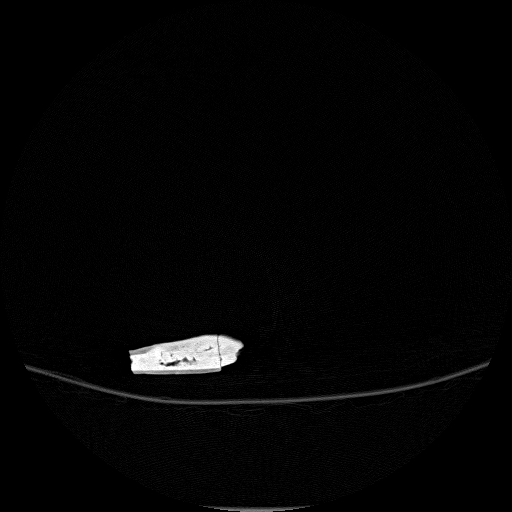

Supplement: S1 Dataset — (ZIP) [file pone.0139800.s001.zip › KA89/KA89A/KA890316.tif]

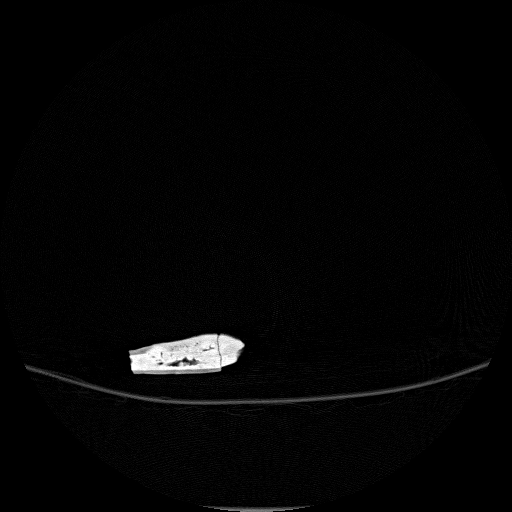

Supplement: S1 Dataset — (ZIP) [file pone.0139800.s001.zip › KA89/KA89A/KA890317.tif]

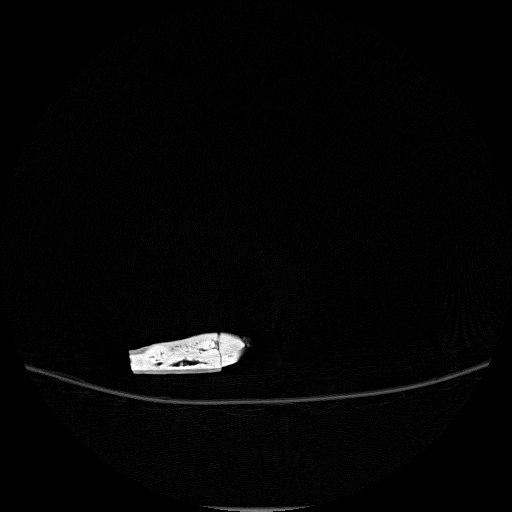

Supplement: S1 Dataset — (ZIP) [file pone.0139800.s001.zip › KA89/KA89A/KA890318.tif]

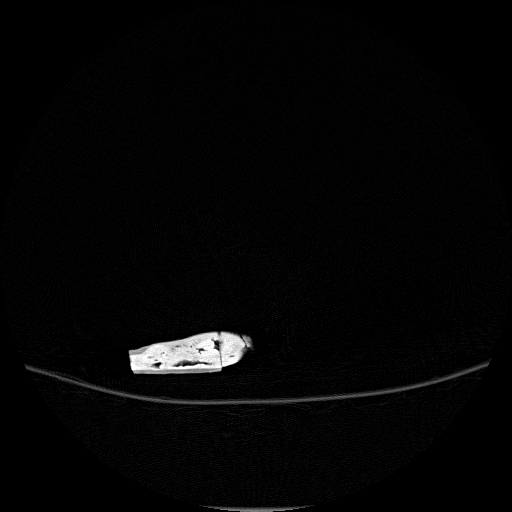

Supplement: S1 Dataset — (ZIP) [file pone.0139800.s001.zip › KA89/KA89A/KA890319.tif]

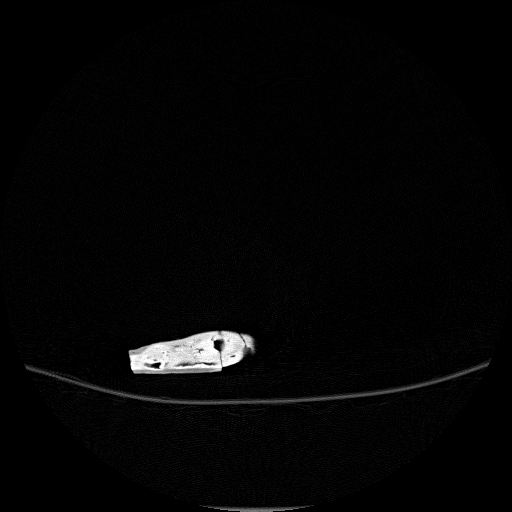

Supplement: S1 Dataset — (ZIP) [file pone.0139800.s001.zip › KA89/KA89A/KA890320.tif]

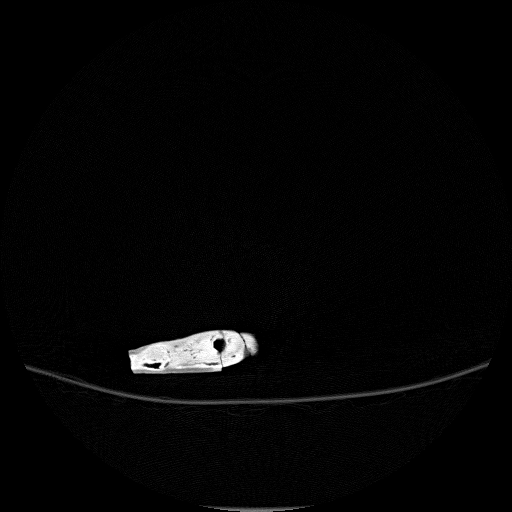

Supplement: S1 Dataset — (ZIP) [file pone.0139800.s001.zip › KA89/KA89A/KA890321.tif]

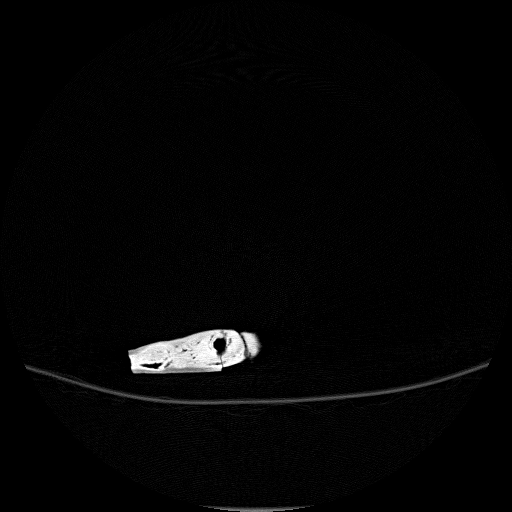

Supplement: S1 Dataset — (ZIP) [file pone.0139800.s001.zip › KA89/KA89A/KA890322.tif]

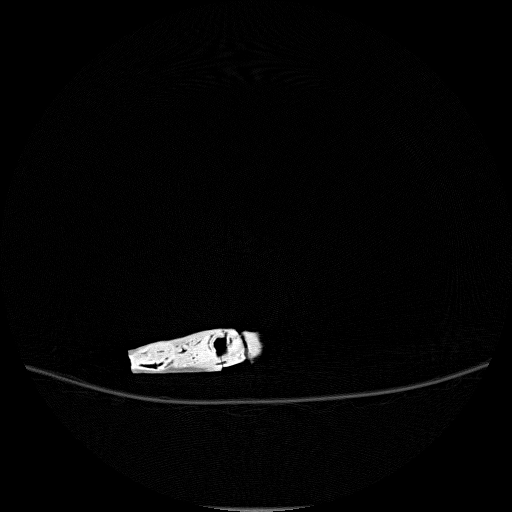

Supplement: S1 Dataset — (ZIP) [file pone.0139800.s001.zip › KA89/KA89A/KA890323.tif]

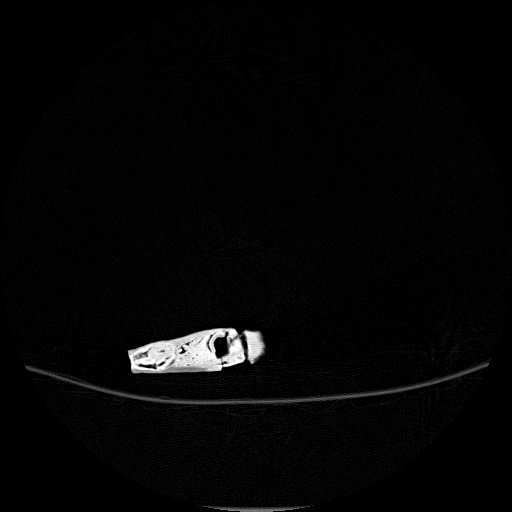

Supplement: S1 Dataset — (ZIP) [file pone.0139800.s001.zip › KA89/KA89A/KA890324.tif]

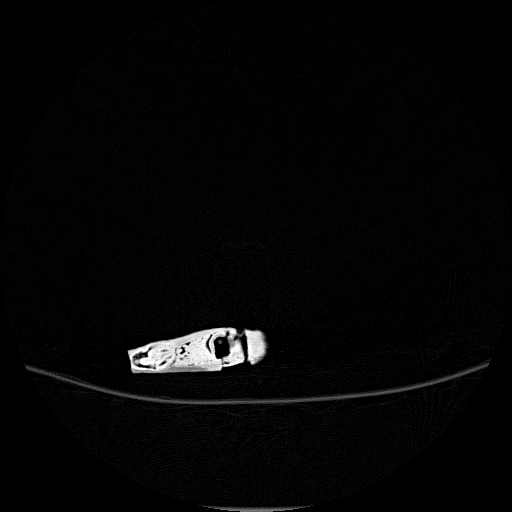

Supplement: S1 Dataset — (ZIP) [file pone.0139800.s001.zip › KA89/KA89A/KA890325.tif]

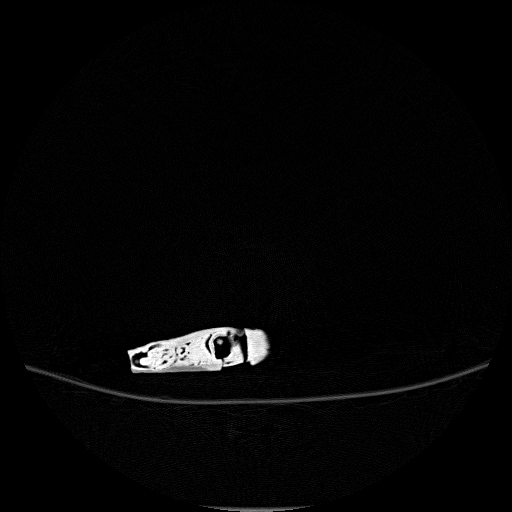

Supplement: S1 Dataset — (ZIP) [file pone.0139800.s001.zip › KA89/KA89A/KA890326.tif]

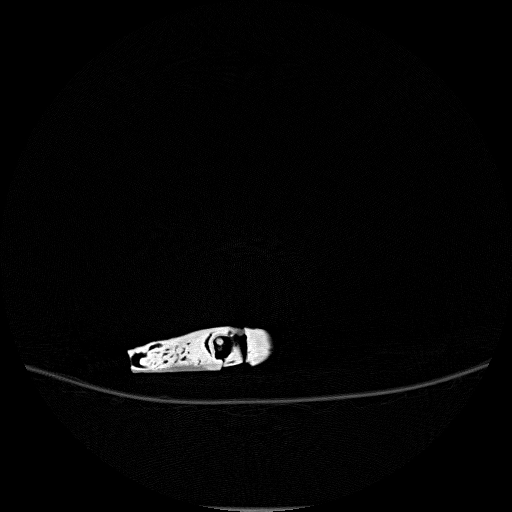

Supplement: S1 Dataset — (ZIP) [file pone.0139800.s001.zip › KA89/KA89A/KA890327.tif]

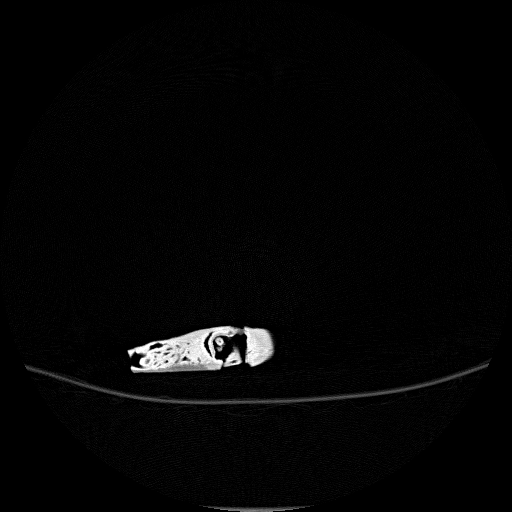

Supplement: S1 Dataset — (ZIP) [file pone.0139800.s001.zip › KA89/KA89A/KA890328.tif]

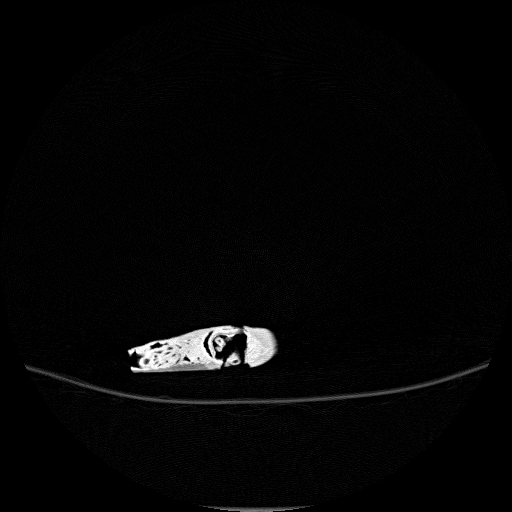

Supplement: S1 Dataset — (ZIP) [file pone.0139800.s001.zip › KA89/KA89A/KA890329.tif]

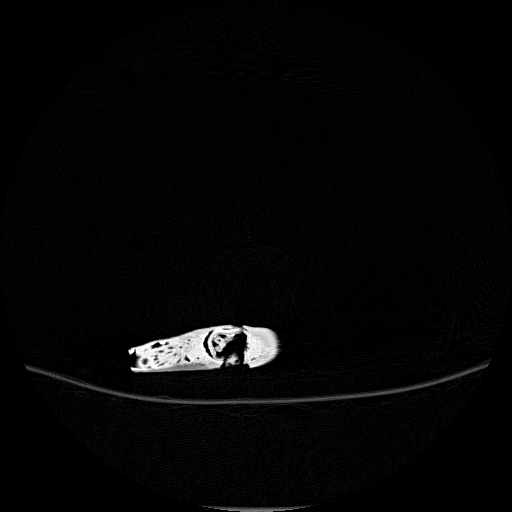

Supplement: S1 Dataset — (ZIP) [file pone.0139800.s001.zip › KA89/KA89A/KA890330.tif]

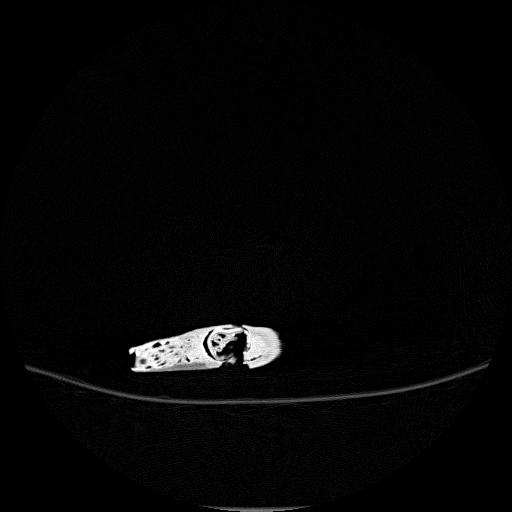

Supplement: S1 Dataset — (ZIP) [file pone.0139800.s001.zip › KA89/KA89A/KA890331.tif]

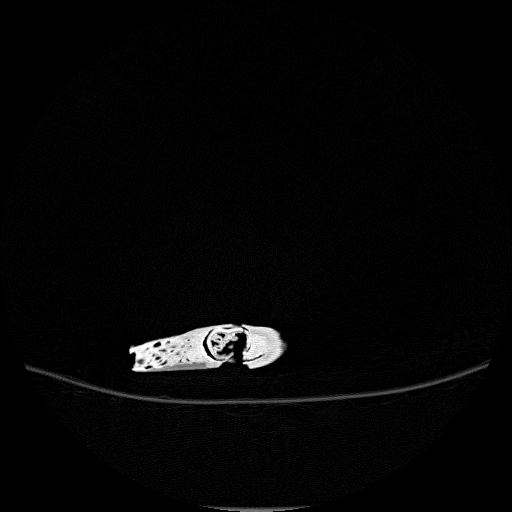

Supplement: S1 Dataset — (ZIP) [file pone.0139800.s001.zip › KA89/KA89A/KA890332.tif]

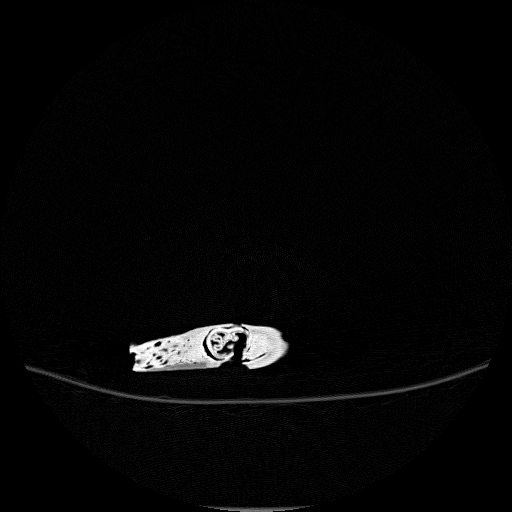

Supplement: S1 Dataset — (ZIP) [file pone.0139800.s001.zip › KA89/KA89A/KA890333.tif]

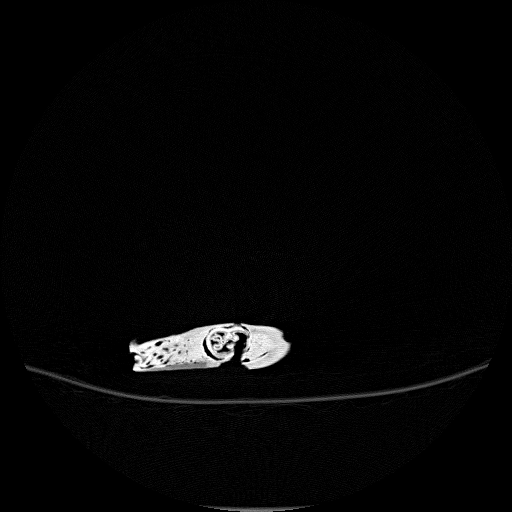

Supplement: S1 Dataset — (ZIP) [file pone.0139800.s001.zip › KA89/KA89A/KA890334.tif]

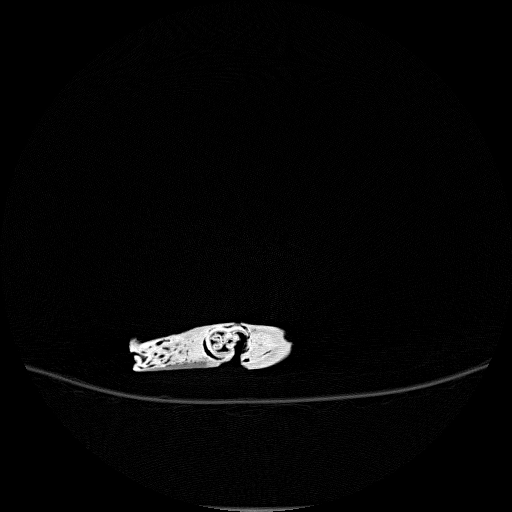

Supplement: S1 Dataset — (ZIP) [file pone.0139800.s001.zip › KA89/KA89A/KA890335.tif]

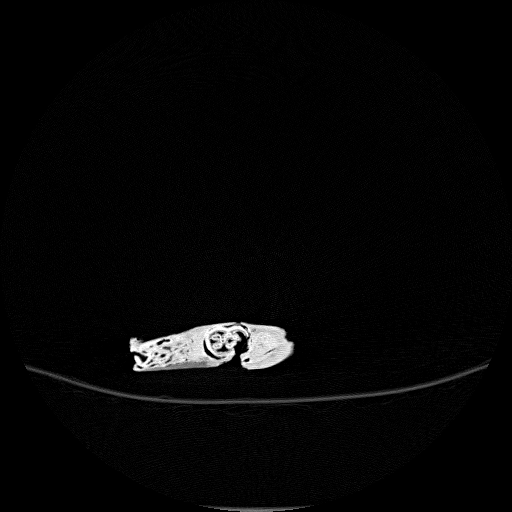

Supplement: S1 Dataset — (ZIP) [file pone.0139800.s001.zip › KA89/KA89A/KA890336.tif]

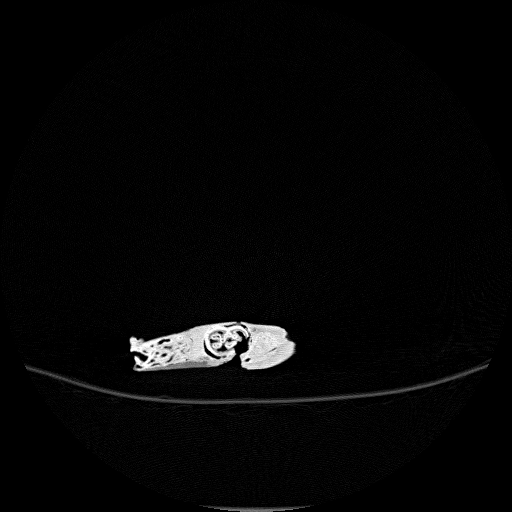

Supplement: S1 Dataset — (ZIP) [file pone.0139800.s001.zip › KA89/KA89A/KA890337.tif]

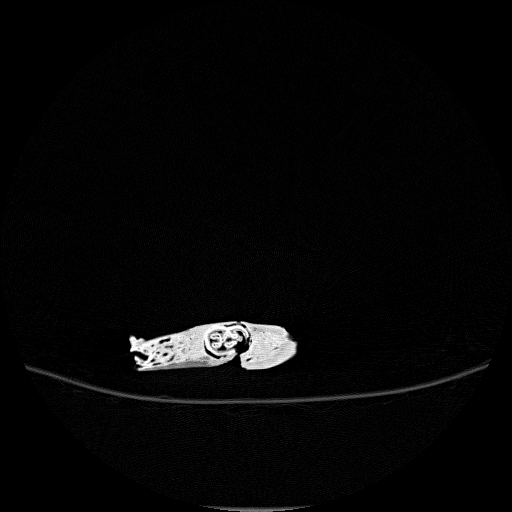

Supplement: S1 Dataset — (ZIP) [file pone.0139800.s001.zip › KA89/KA89A/KA890338.tif]

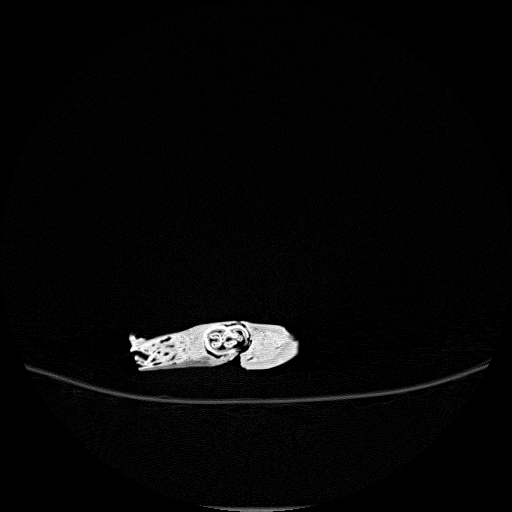

Supplement: S1 Dataset — (ZIP) [file pone.0139800.s001.zip › KA89/KA89A/KA890339.tif]

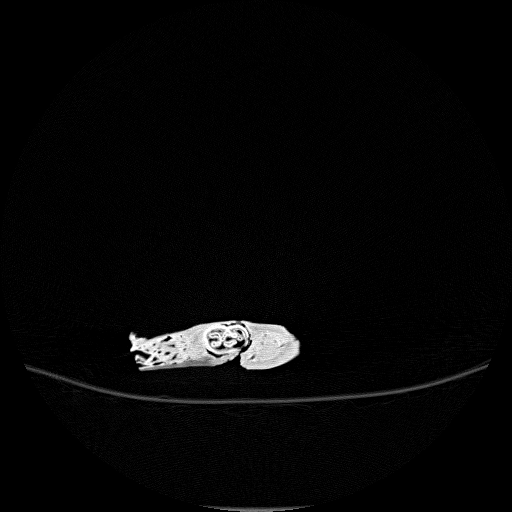

Supplement: S1 Dataset — (ZIP) [file pone.0139800.s001.zip › KA89/KA89A/KA890340.tif]

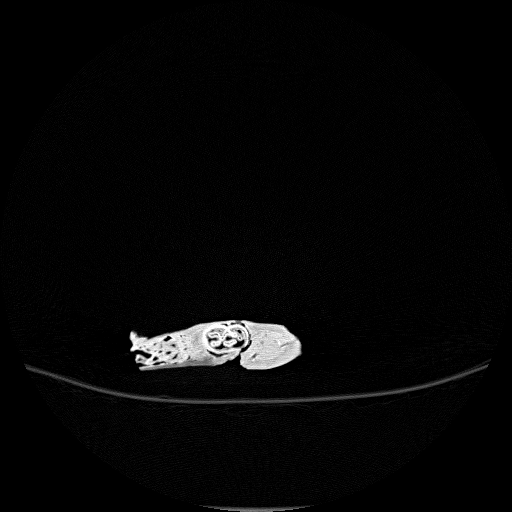

Supplement: S1 Dataset — (ZIP) [file pone.0139800.s001.zip › KA89/KA89A/KA890341.tif]

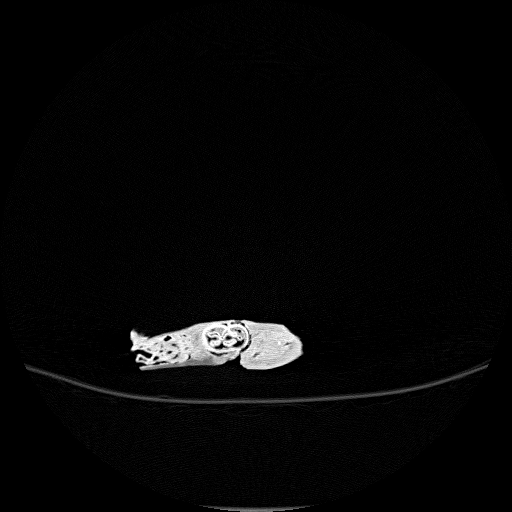

Supplement: S1 Dataset — (ZIP) [file pone.0139800.s001.zip › KA89/KA89A/KA890342.tif]

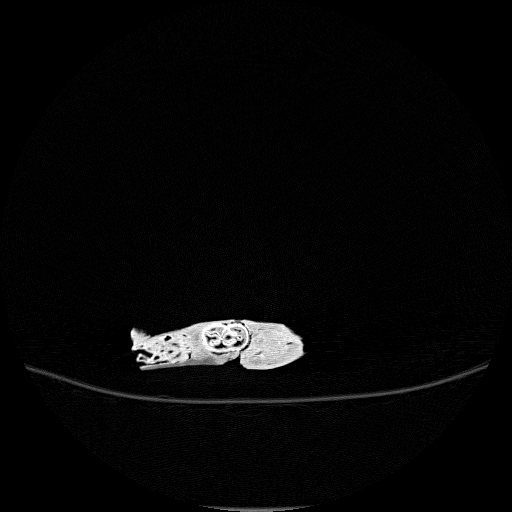

Supplement: S1 Dataset — (ZIP) [file pone.0139800.s001.zip › KA89/KA89A/KA890343.tif]

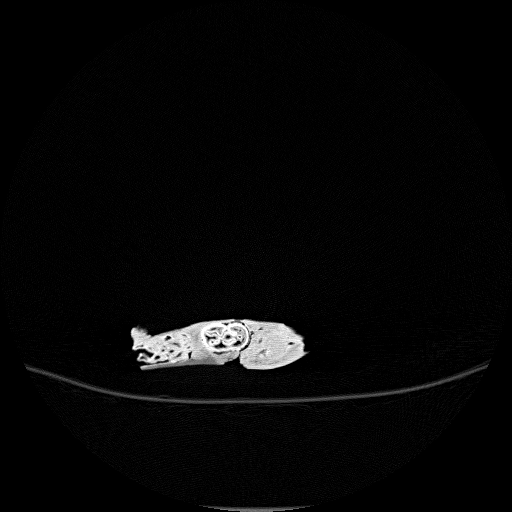

Supplement: S1 Dataset — (ZIP) [file pone.0139800.s001.zip › KA89/KA89A/KA890344.tif]

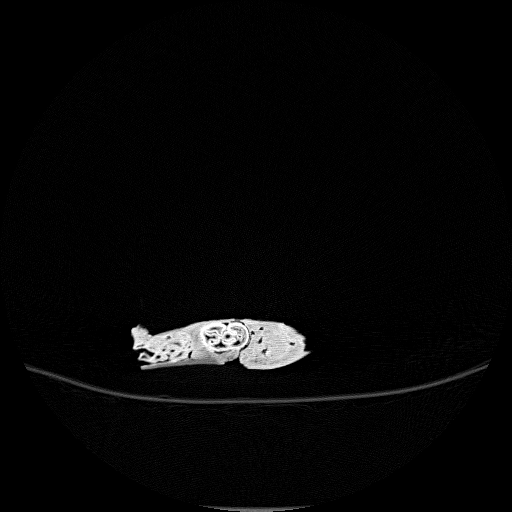

Supplement: S1 Dataset — (ZIP) [file pone.0139800.s001.zip › KA89/KA89A/KA890345.tif]

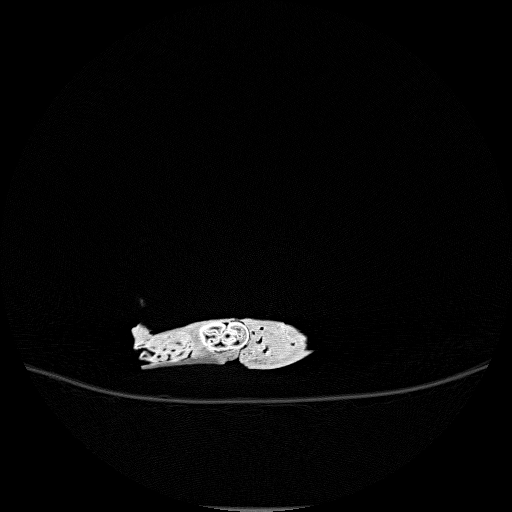

Supplement: S1 Dataset — (ZIP) [file pone.0139800.s001.zip › KA89/KA89A/KA890346.tif]

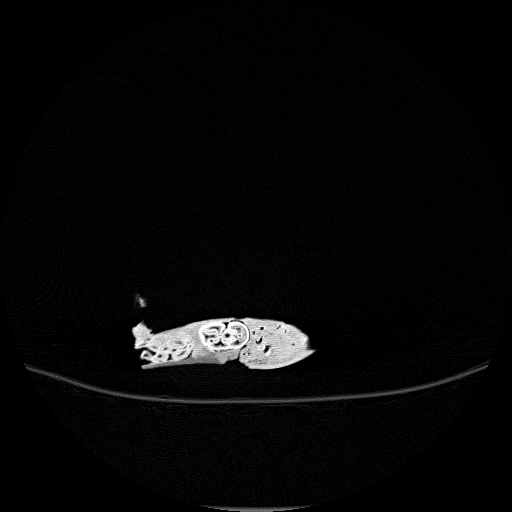

Supplement: S1 Dataset — (ZIP) [file pone.0139800.s001.zip › KA89/KA89A/KA890347.tif]

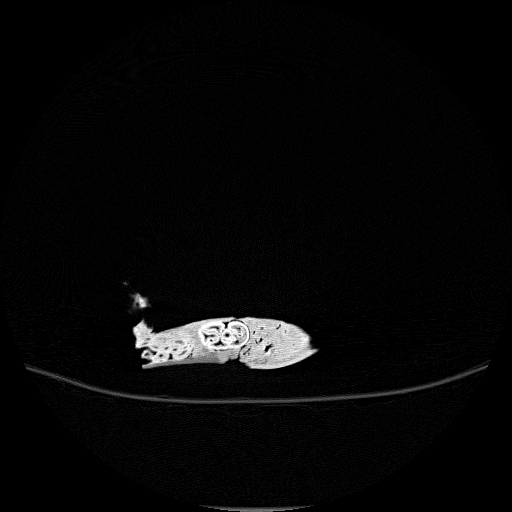

Supplement: S1 Dataset — (ZIP) [file pone.0139800.s001.zip › KA89/KA89A/KA890348.tif]

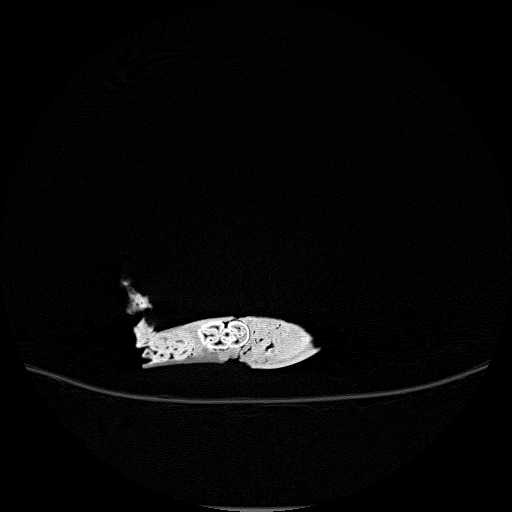

Supplement: S1 Dataset — (ZIP) [file pone.0139800.s001.zip › KA89/KA89A/KA890349.tif]

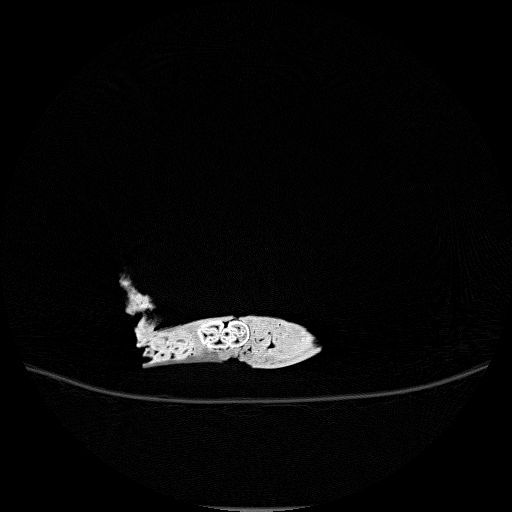

Supplement: S1 Dataset — (ZIP) [file pone.0139800.s001.zip › KA89/KA89A/KA890350.tif]

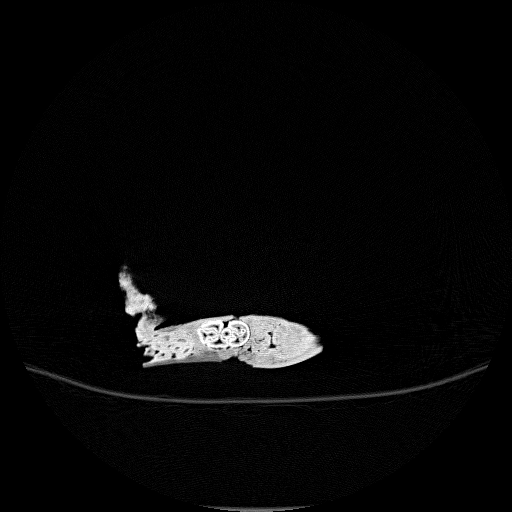

Supplement: S1 Dataset — (ZIP) [file pone.0139800.s001.zip › KA89/KA89A/KA890351.tif]

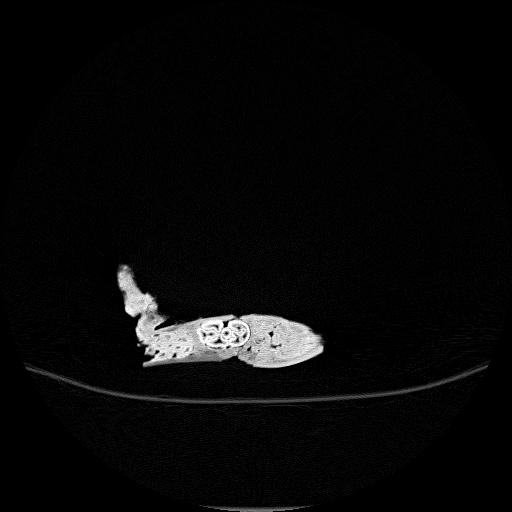

Supplement: S1 Dataset — (ZIP) [file pone.0139800.s001.zip › KA89/KA89A/KA890352.tif]

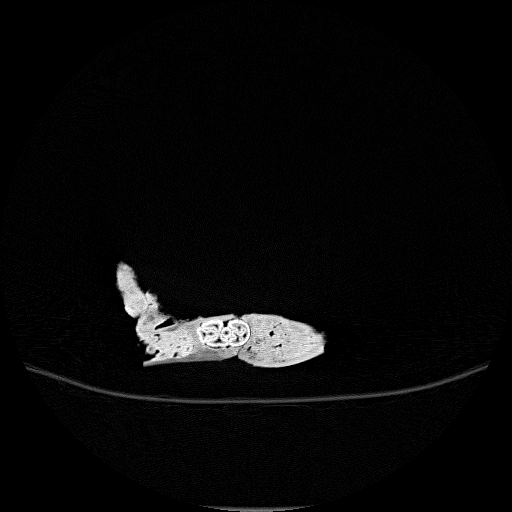

Supplement: S1 Dataset — (ZIP) [file pone.0139800.s001.zip › KA89/KA89A/KA890353.tif]

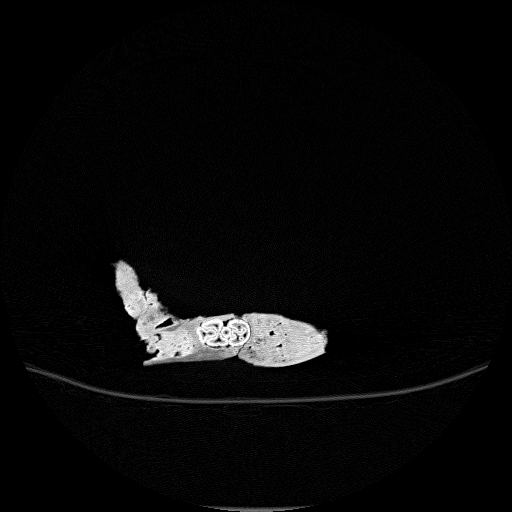

Supplement: S1 Dataset — (ZIP) [file pone.0139800.s001.zip › KA89/KA89A/KA890354.tif]

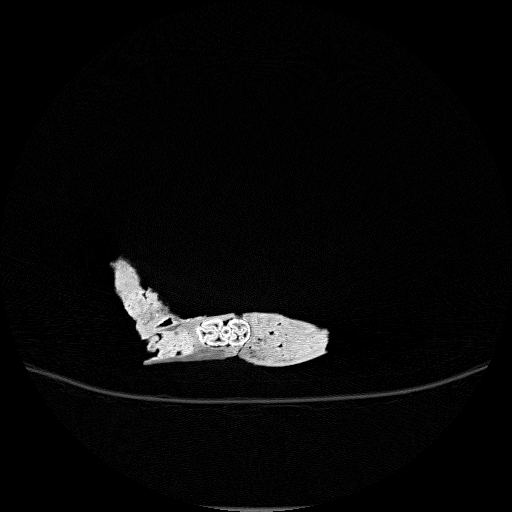

Supplement: S1 Dataset — (ZIP) [file pone.0139800.s001.zip › KA89/KA89A/KA890355.tif]

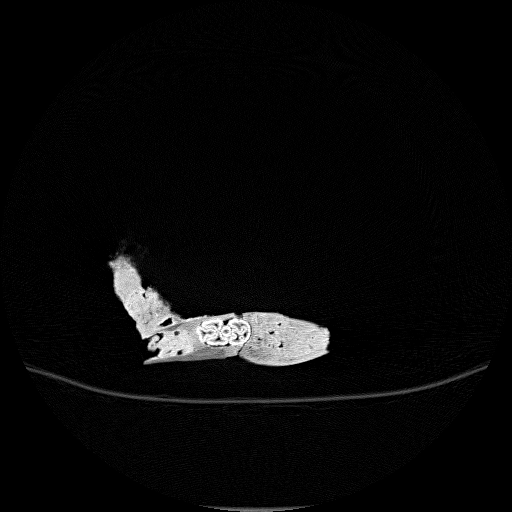

Supplement: S1 Dataset — (ZIP) [file pone.0139800.s001.zip › KA89/KA89A/KA890356.tif]

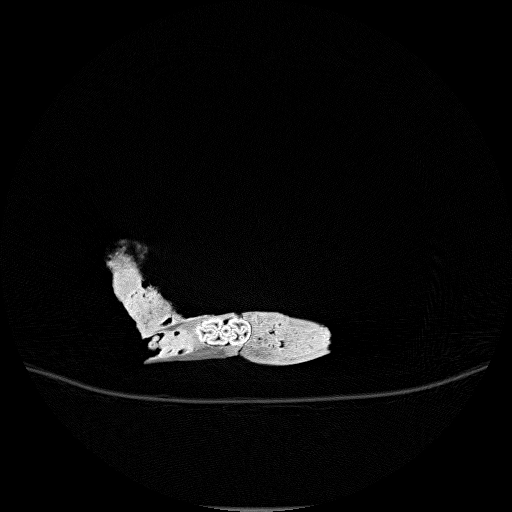

Supplement: S1 Dataset — (ZIP) [file pone.0139800.s001.zip › KA89/KA89A/KA890357.tif]

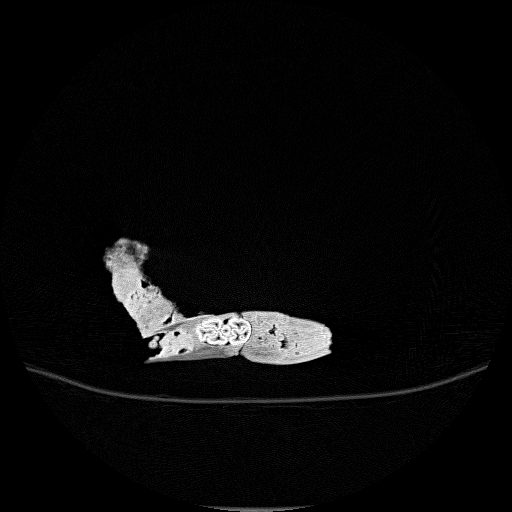

Supplement: S1 Dataset — (ZIP) [file pone.0139800.s001.zip › KA89/KA89A/KA890358.tif]

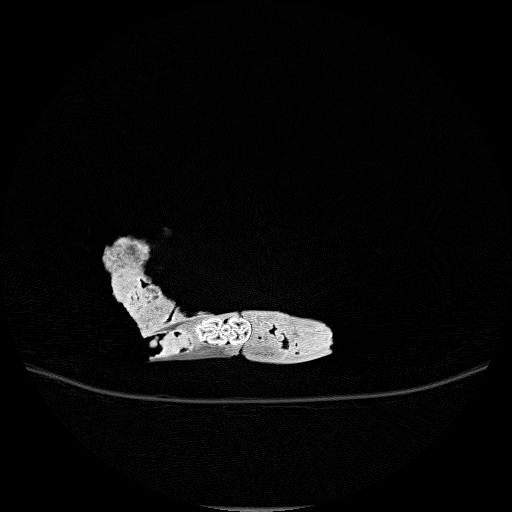

Supplement: S1 Dataset — (ZIP) [file pone.0139800.s001.zip › KA89/KA89A/KA890359.tif]

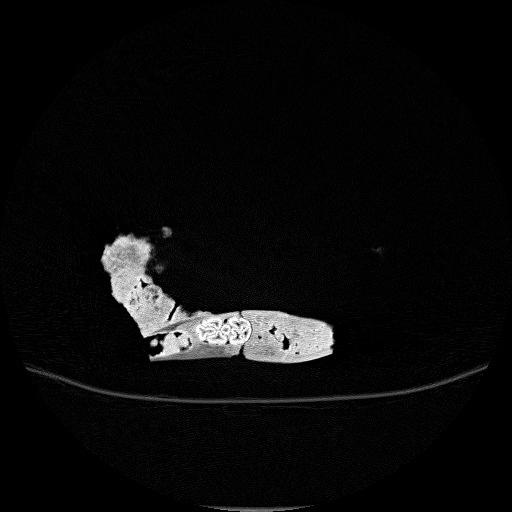

Supplement: S1 Dataset — (ZIP) [file pone.0139800.s001.zip › KA89/KA89A/KA890360.tif]

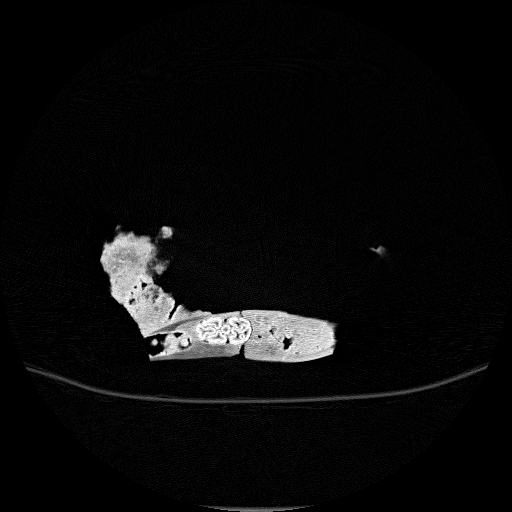

Supplement: S1 Dataset — (ZIP) [file pone.0139800.s001.zip › KA89/KA89A/KA890361.tif]

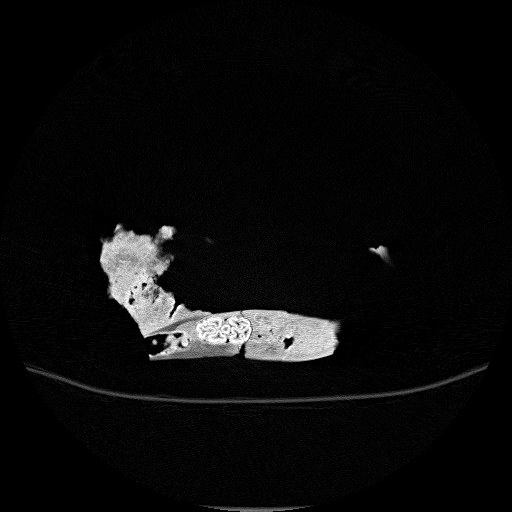

Supplement: S1 Dataset — (ZIP) [file pone.0139800.s001.zip › KA89/KA89A/KA890362.tif]

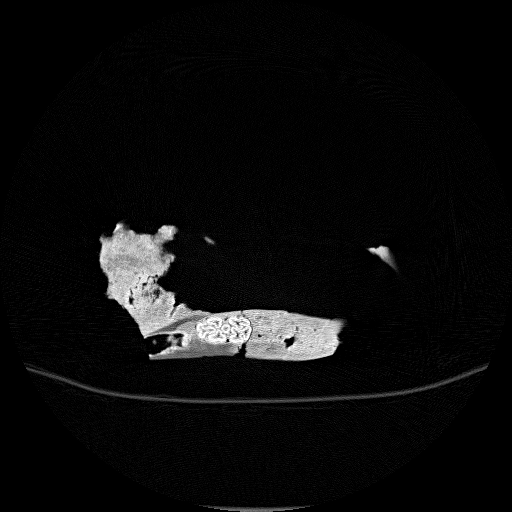

Supplement: S1 Dataset — (ZIP) [file pone.0139800.s001.zip › KA89/KA89A/KA890363.tif]

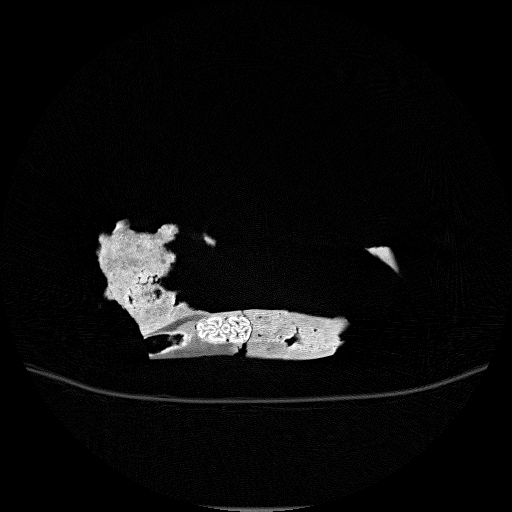

Supplement: S1 Dataset — (ZIP) [file pone.0139800.s001.zip › KA89/KA89A/KA890364.tif]

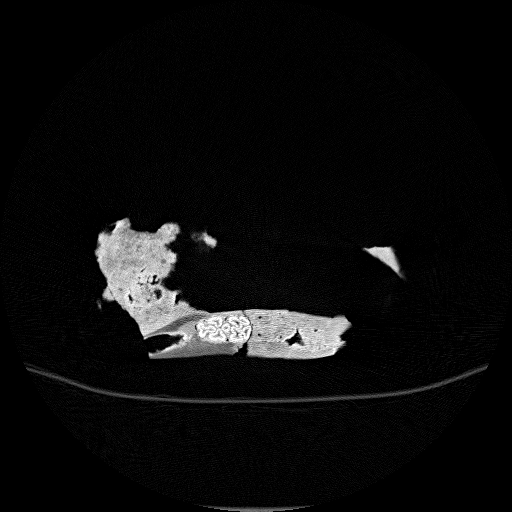

Supplement: S1 Dataset — (ZIP) [file pone.0139800.s001.zip › KA89/KA89A/KA890365.tif]

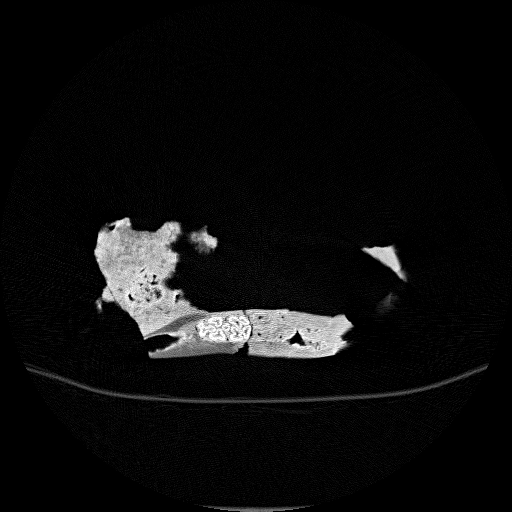

Supplement: S1 Dataset — (ZIP) [file pone.0139800.s001.zip › KA89/KA89A/KA890366.tif]

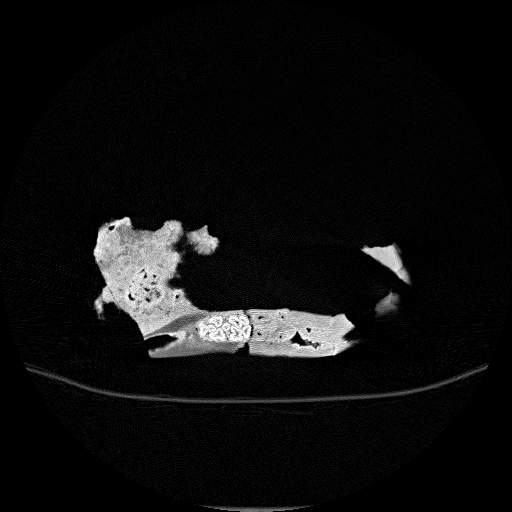

Supplement: S1 Dataset — (ZIP) [file pone.0139800.s001.zip › KA89/KA89A/KA890367.tif]

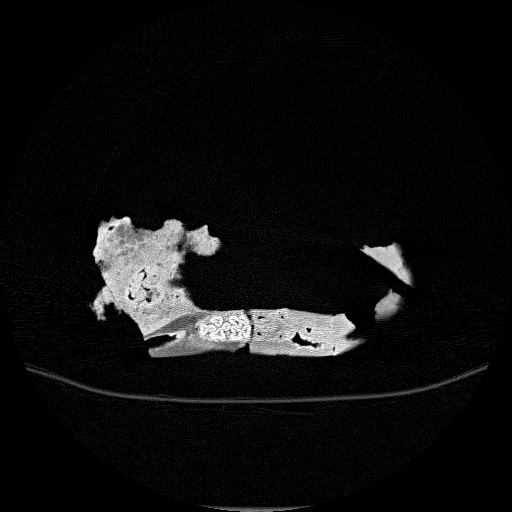

Supplement: S1 Dataset — (ZIP) [file pone.0139800.s001.zip › KA89/KA89A/KA890368.tif]

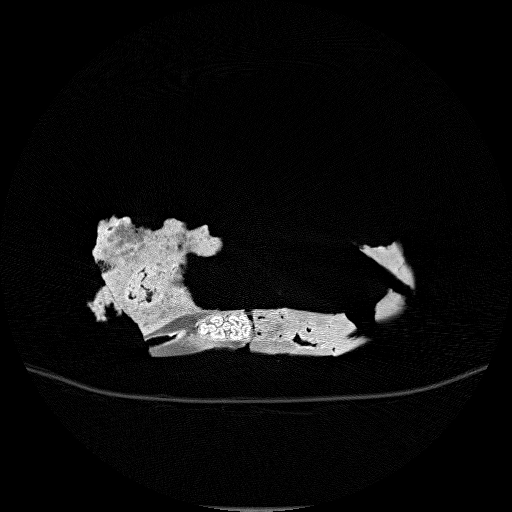

Supplement: S1 Dataset — (ZIP) [file pone.0139800.s001.zip › KA89/KA89A/KA890369.tif]

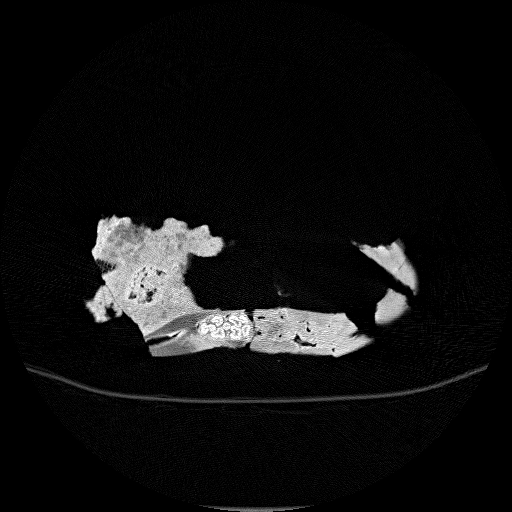

Supplement: S1 Dataset — (ZIP) [file pone.0139800.s001.zip › KA89/KA89A/KA890370.tif]

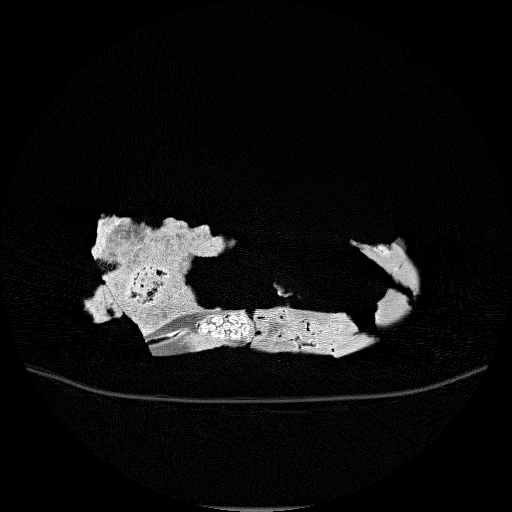

Supplement: S1 Dataset — (ZIP) [file pone.0139800.s001.zip › KA89/KA89A/KA890371.tif]

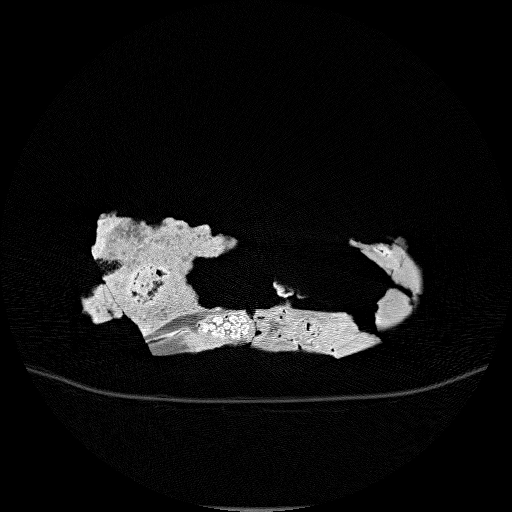

Supplement: S1 Dataset — (ZIP) [file pone.0139800.s001.zip › KA89/KA89A/KA890372.tif]

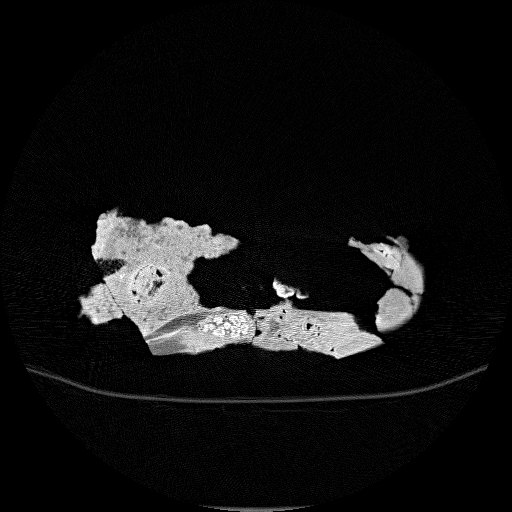

Supplement: S1 Dataset — (ZIP) [file pone.0139800.s001.zip › KA89/KA89A/KA890373.tif]

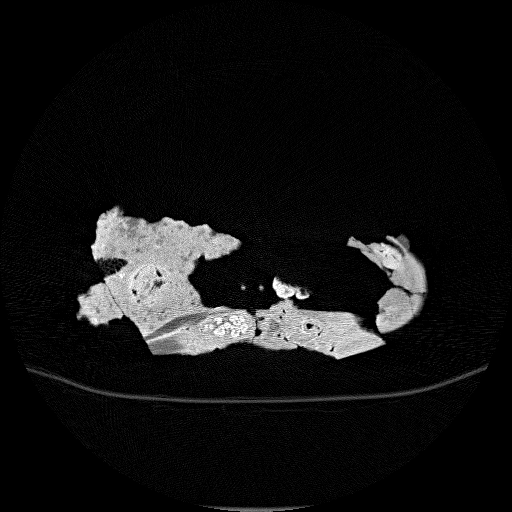

Supplement: S1 Dataset — (ZIP) [file pone.0139800.s001.zip › KA89/KA89A/KA890374.tif]

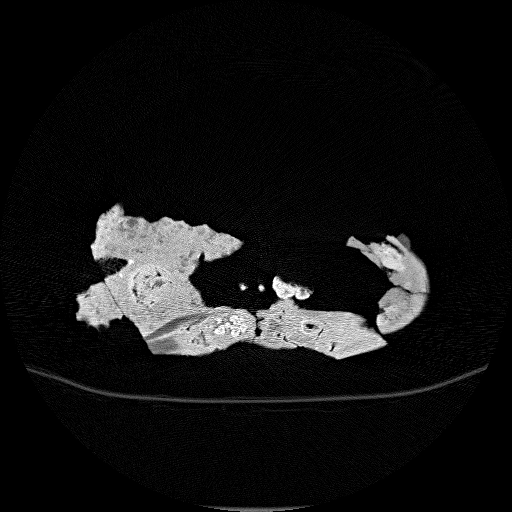

Supplement: S1 Dataset — (ZIP) [file pone.0139800.s001.zip › KA89/KA89A/KA890375.tif]

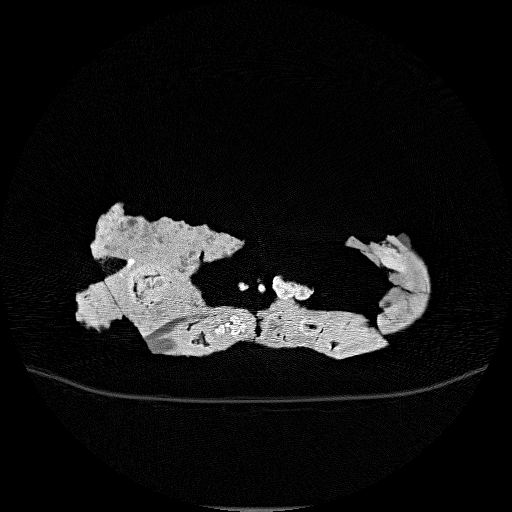

Supplement: S1 Dataset — (ZIP) [file pone.0139800.s001.zip › KA89/KA89A/KA890376.tif]

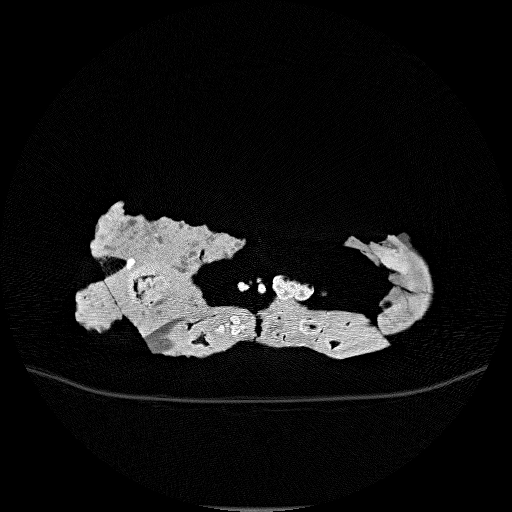

Supplement: S1 Dataset — (ZIP) [file pone.0139800.s001.zip › KA89/KA89A/KA890377.tif]

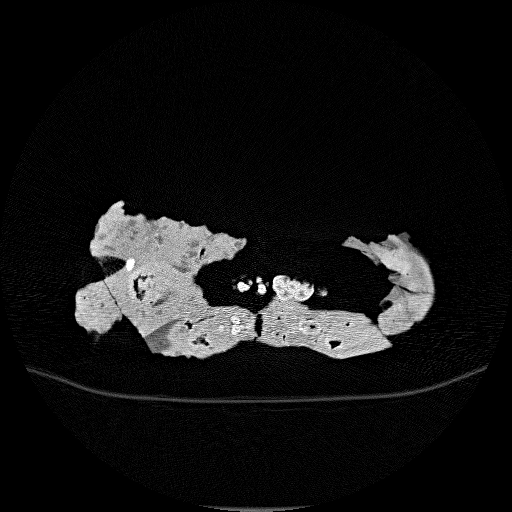

Supplement: S1 Dataset — (ZIP) [file pone.0139800.s001.zip › KA89/KA89A/KA890378.tif]

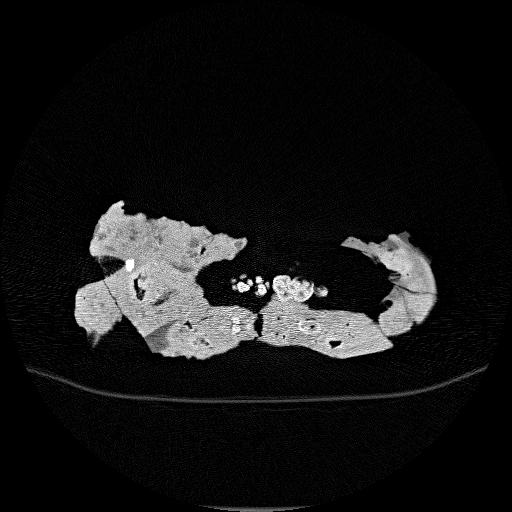

Supplement: S1 Dataset — (ZIP) [file pone.0139800.s001.zip › KA89/KA89A/KA890379.tif]

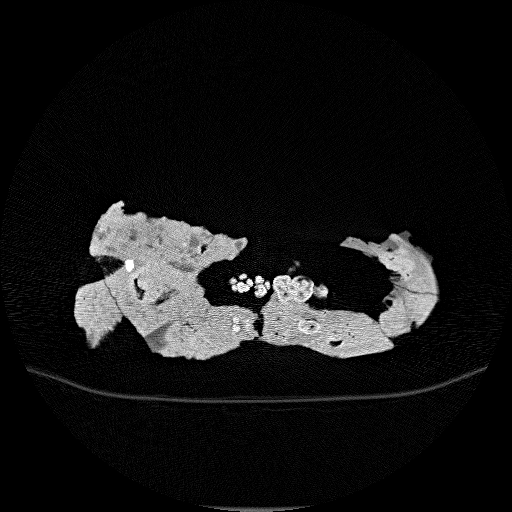

Supplement: S1 Dataset — (ZIP) [file pone.0139800.s001.zip › KA89/KA89A/KA890380.tif]

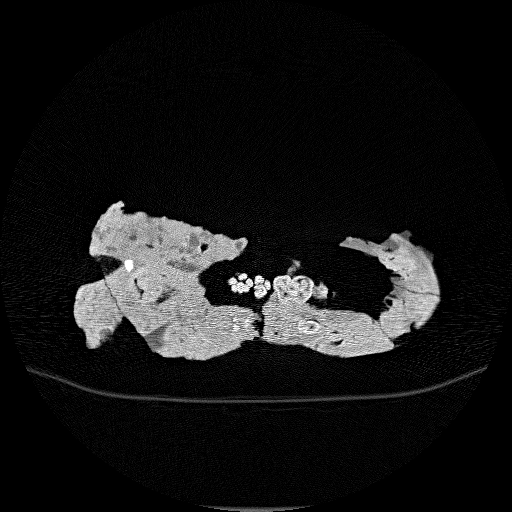

Supplement: S1 Dataset — (ZIP) [file pone.0139800.s001.zip › KA89/KA89A/KA890381.tif]

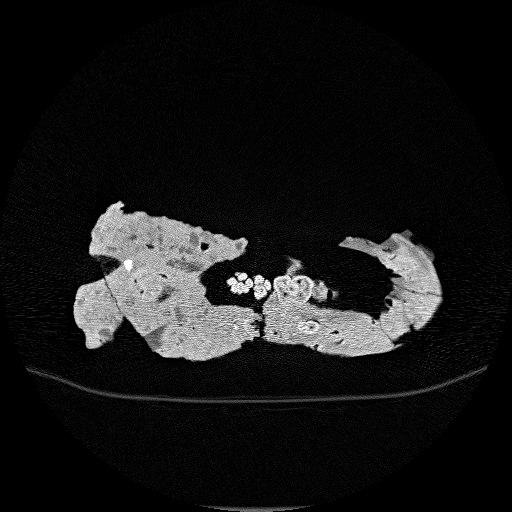

Supplement: S1 Dataset — (ZIP) [file pone.0139800.s001.zip › KA89/KA89A/KA890382.tif]

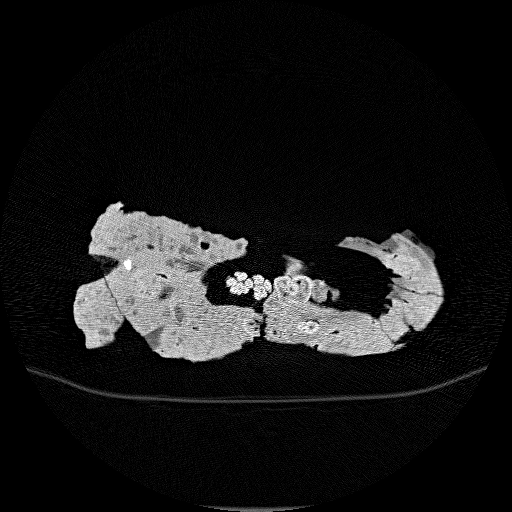

Supplement: S1 Dataset — (ZIP) [file pone.0139800.s001.zip › KA89/KA89A/KA890383.tif]

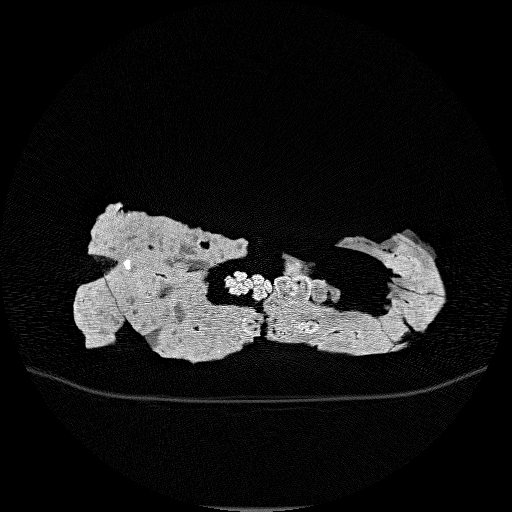

Supplement: S1 Dataset — (ZIP) [file pone.0139800.s001.zip › KA89/KA89A/KA890384.tif]
