# Supplementary material for: Surface Model and Tomographic Archive of Fossil Primate and Other Mammal Holotype and Paratype Specimens of the Ditsong National Museum of Natural History, Pretoria, South Africa
Source: PLoS One. 2015 Oct 6;10(10):e0139800. doi: 10.1371/journal.pone.0139800 (PMC4595468; doi:10.1371/journal.pone.0139800)
Supplement: S1 Table — Holotype specimens are in bold. Those specimens with additional available tomographic data are underlined. (DOCX) [file pone.0139800.s005.docx]

| **Original Taxonomic Attribution** | **Specimen Number** | **Deposit** | **Original/Primary Description(s)** | **Current Taxonomic Allocation** | **Additional Description(s)** |
| --- | --- | --- | --- | --- | --- |
| Order Primates |  |  |  |  |  |
| Family Cercopithecidae |  |  |  |  |  |
| Subfamily Cercopithecinae |  |  |  |  |  |
| *Dinopithecus ingens* | SB 2 | Skurweberg^a^ | 8-9 | *Papio hamadryas robinsoni* | 9 |
| *Dinopithecus ingens* | SB 3 | Skurweberg^a^ | 10 | *Dinopithecus ingens* | 9 |
| ***Dinopithecus ingens*** | SB 7 | Skurweberg^a^ | 10 | ***Dinopithecus ingens*** | 9 |
| *Dinopithecus ingens* | SK 554 | Swartkrans Member 1 | 9 | *Dinopithecus ingens* |  |
| *Dinopithecus ingens* | SK 599^b^ | Swartkrans Member 1 | 1 | *Dinopithecus ingens* |  |
| *Dinopithecus ingens* | SK 604a | Swartkrans Member 1 | 1 | *Dinopithecus ingens* |  |
| ***Parapapio angusticeps*** | KA 194^c^ | Kromdraai A | 8 | ***Papio angusticeps*** | 9 |
| *Papio angusticeps* | CO 100^b^ | Cooper’s A | 9 | *Papio angusticeps* |  |
| ***Papio robinsoni*** | SK 555 | Swartkrans Member 1 | 9 | *Papio hamadryas robinsoni* |  |
| ***Papio spelaeus*** | TM 211 | Unknown (Holocene?) | 11 | *Papio hamadryas ursinus* | 9 |
| ***Parapapio broomi*** | STS 564 (1238)^d^ | Sterkfontein Member 4 | 12 | ***Parapapio broomi*** | 9 |
| *Parapapio broomi* | STS 262 | Sterkfontein Member 4 | 9 | *Papio izodi* | 13-14 |
| *Parapapio broomi* | STS 562 (501)^d^ | Sterkfontein Member 4 | 8 | *Parapapio broomi* | 9 |
| ***Parapapio coronatus*** | KA 195 (KB 122) | Kromdraai B | 15 | *Cercopithecoides williamsi*^e^ | 16 |
| ***Parapapio jonesi*** | STS 565 | Sterkfontein Member 4 | 8 | ***Parapapio jonesi*** | 9 |
| ***Parapapio major*** | KA 193 | Kromdraai A | 8 | ***Gorgopithecus major*** | 9 |
| ***Parapapio whitei*** | STS 563 (1460)^d^ | Sterkfontein Member 4 | 8 | *Parapapio broomi* | 14 |
| ***Simopithecus danieli*** | SK 563 | Swartkrans Member 1 | 9 | *Theropithecus oswaldi oswaldi* | 17-18 |
| ***Simopithecus danieli*** | SK 402 | Swartkrans Member 1 | 9 | *Theropithecus oswaldi oswaldi* | 17-18 |
| ***Simopithecus danieli*** | SK 405 | Swartkrans Member 1 | 9 | *Theropithecus oswaldi oswaldi* | 17-18 |
|  |  |  |  |  |  |
| Subfamily Colobinae^f^ |  |  |  |  |  |
| ***Cercopithecoides molletti*** | SK 551^g^ | Swartkrans Member 1 | 9 | *Cercopithecoides williamsi* |  |
| *Cercopithecoides williamsi* | STS 394a & b^b^ | Sterkfontein Member 4 | 9 | *Cercopithecoides williamsi* |  |
|  |  |  |  |  |  |
| Order Carnivora |  |  |  |  |  |
| Family Canidae |  |  |  |  |  |
| Tribe Canini |  |  |  |  |  |
| ***Canis antiquus*** | STS 1582 | Minaar’s or  Sterkfontein | 19 | *Canis mesomelas* | 20-21 |
| *Canis antiquus* | TM 1583 | Minaar’s | 20 | *Canis mesomelas* | 21 |
| ***Canis atrox*** | KA 1288 | Kromdraai A | 20 | *Canis* sp. | 22 |
| *Canis atrox* | KA 1556 | Kromdraai A | 20 | *Canis* sp. | 22 |
| ***Canis brevirostris*** | STS 137 | Sterkfontein Member 4 | 21 | ***Canis brevirostris*** |  |
| ***Canis mesomelas pappos*** | KA 73 | Kromdraai A | 21 | *Canis mesomelas* |  |
| *Canis mesomelas pappos* | KA 71 | Kromdraai A | 21 | *Canis mesomelas* |  |
| ***Thos terblanchei*** | KA 1290 | Kromdraai A | 20 | ***Nyctereutes terblanchei*** | 23-24 |
| Tribe Vulpini |  |  |  |  |  |
| ***Vulpes pattisoni*** | TM 1553 | Taung | 20 | ***Vulpes pattisoni*** |  |
| ***Vulpes pulcher*** | KA 1289 | Kromdraai A | 19 | ***Vulpes pulcher*** | 21 |
|  |  |  |  |  |  |
| Family Felidae |  |  |  |  |  |
| Subfamily Felinae |  |  |  |  |  |
| ***Megantereon barlowi*** | TM 1541 | Sterkfontein Member 4 | 10 | ***Dinofelis barlowi*** | 25-26 |
| ***Megantereon barlowi*** | TM 1542 | Sterkfontein Member 4 | 10 | ***Dinofelis barlowi*** | 25-26 |
| *Machairodus transvaalensis* | TM 1579 | Sterkfontein^h^ | 10 | *Dinofelis barlowi* | 25-26 |
| *Dinofelis barlowi* | BF 55-22 | Bolt’s Farm  Pit 23 | 26 | *Dinofelis barlowi* |  |
| *Dinofelis barlowi* | BF 55-23 | Bolt’s Farm  Pit 23 | 26 | *Dinofelis barlowi* |  |
| ***Therailurus piveteaui*** | KA 61 | Kromdraai A | 25 | ***Dinofelis piveteaui*** |  |
| *Therailurus piveteaui* | KA 62 | Kromdraai A | 25 | *Dinofelis piveteaui* |  |
| *Therailurus piveteaui* | KA 63 | Kromdraai A | 25 | *Dinofelis piveteaui* |  |
|  |  |  |  |  |  |
| Subfamily Machairodontinae |  |  |  |  |  |
| ***Machairodus transvaalensis*** | STS 130-299 | Sterkfontein^i^ | 27 | ***Machairodus transvaalensis*** | 28-29 |
| *Machairodus transvaalensis* | TM 1577 | Sterkfontein^i^ | 28 | *Machairodus transvaalensis* | 29 |
| ***‘Felis’ whitei*** | TM 856 | Skurweberg^a^ | 10 | ***Megantereon whitei*** | 30 |
| ***Megantereon euryndon*** | KA 64 | Kromdraai A | 28 | *Megantereon whitei* | 30 |
| ***Megantereon gracile*** | STS 1558 | Sterkfontein Member 4^j^ | 20 | *Megantereon whitei* | 30 |
|  |  |  |  |  |  |
| Subfamily Pantherinae |  |  |  |  |  |
| ***Felis crassidens*** | KA 87 | Kromdraai A | 20 | *Panthera pardus* | 31 |
| ***Felis shawi*** | BF 1555 | Bolt’s Farm^k^ | 20 | *Panthera leo* | 29, 32 |
| ***Panthera pardus incurva*** | SK 349 | Swartkrans Member 1 | 33 | *Panthera pardus* |  |
|  |  |  |  |  |  |
| Family Herpestidae |  |  |  |  |  |
| ***Herpestes mesotes*** | KA 86 | Kromdraai A | 34 | ***Atilax mesotes*** | 35 |
|  |  |  |  |  |  |
| Family Hyaenidae |  |  |  |  |  |
| ***Crocuta spelaea capensis*** | KA 56 | Kromdraai A | 27 | *Crocuta crocuta* | 36 |
| *Crocuta spelaea capensis* | KA 57a | Kromdraai A | 37 | *Crocuta crocuta* | 36 |
| ***Crocuta ultra ultra*** | KA 58 | Kromdraai A | 37 | *Crocuta crocuta*^l^ | 36 |
| ***Crocuta ultra latidens*** | CT 1 | Clyde Trading Company | 37 | *Crocuta crocuta* | 36 |
| ***Crocuta ultra latidens*** | CT 2 | Clyde Trading Company | 37 | *Crocuta crocuta* | 36 |
| ***Crocuta ultra latidens*** | CT 3 | Clyde Trading Company | 37 | *Crocuta crocuta* | 36 |
| ***Crocuta venustula*** | SK 317 | Swartkrans Member 1 | 38 | *Crocuta crocuta* | 36 |
| ***Hyaena bellax*** | KA 55 | Kromdraai A | 37 | ***Pachycrocuta brevirostris*** | 22, 35 |
| ***Hyaena brunnea dispar*** | SK 326 | Swartkrans Member 1 | 38 | *Parahyaena brunnea* | 22 |
| ***Hyaena striata***^m^ | KA 211Y | Kromdraai A | 27 | *Hyaena* cf. *H. hyaena* |  |
| ***Leecyaena forfex*** | SK 314 | Swartkrans Member 1 | 28 | *Hyaena hyaena* | 39 |
| *Leecyaena forfex* | SK 315 | Swartkrans Member 1 | 28 | *Parahyaena brunnea* | 39 |
| *Leecyaena forfex* | SK 316 | Swartkrans Member 1 | 28 | *Parahyaena brunnea* | 39 |
| ***Lycyaena nitidula*** | SK 301 | Swartkrans Member 1 | 28 | ***Chasmaporthetes nitidula*** | 35, 39 |
| ***Lycyaena silberbergi*** | STS 130 | Sterkfontein Member 2 | 20 | ***Lycyaenops silberbergi*** | 25, 35 |
| *Lycyaena silberbergi* | STS 126 | Sterkfontein Member 4 | 20 | *Lycyaenops silberbergi* | 25, 35 |
| *Lycyaena silberbergi* | SK 300 | Swartkrans Member 1 | 28 | *Lycyaenops silberbergi* | 25, 35 |
|  |  |  |  |  |  |
| Order Artiodactyla |  |  |  |  |  |
| Family Bovidae |  |  |  |  |  |
| Subfamily Caprinae |  |  |  |  |  |
| ***Bos makapaani*** | TM 315 | Buffalo Cave | 19 | ***‘Bos’ makapaani*** | 40 |
|  |  |  |  |  |  |
| Family Suidae |  |  |  |  |  |
| *Metridiochoerus andrewsi* | STS 3074a & b | Sterkfontein Member 4 | 41 | *Metridiochoerus* sp. |  |
| *Notochoerus meadowsi* | BF 1 | Bolt’s Farm | 20 | *Metridiochoerus andrewsi* | 41-43 |
| ***Phacochoerus antiquus*** | KA 89a & b | Kromdraai A | 20 | ***Phacochoerus antiquus***^n^ | 41-43 |
| *Phacochoerus antiquus* | SK 382 | Swartkrans Member 2 | 42 | *Phacochoerus antiquus*^n^ | 41, 44 |
| *Phacochoerus modestus* | BF 3-355 | Bolts’ Farm Pit 3 | 41 | *Phacochoerus antiquus*^n^ |  |
| *Phacochoerus modestus* | SK 4005 | Swartkrans Member 2 | 1, 41 | *Phacochoerus antiquus*^n^ |  |
| Suidae indet. | SE 1069-1 | Sterkfontein Member 5 | 1 | Suidae indet. |  |
|  |  |  |  |  |  |
| Order Eulipotyphla |  |  |  |  |  |
| Family Erinaceidae |  |  |  |  |  |
| ***Atelerix major*** | TM 1544 | Bolt’s Farm^o^ | 19 | ***Erinaceus (Atelerix) broomi*** | 20, 44 |
|  |  |  |  |  |  |
| Order Hyracoidea |  |  |  |  |  |
| Family Procaviidae |  |  |  |  |  |
| ***Procavia obermyerae*** | ST 106  (TM 1462) | Gladysvale | 10 | *Procavia transvaalensis* | 45 |
| ***Procavia robertsi*** | ST 105 | Sterkfontein Member 4 | 20 | *Procavia antiqua* | 45 |
| *Procavia transvaalensis* | CO 1 | Cooper’s A | 45 | *Procavia transvaalensis* |  |
| *Procavia transvaalensis* | CO 10 | Cooper’s A | 45 | *Procavia transvaalensis* |  |
| *Procavia transvaalensis* | CO 11 | Cooper’s A | 45 | *Procavia transvaalensis* |  |
| *Procavia transvaalensis* | CO 12a | Cooper’s A | 45 | *Procavia transvaalensis* |  |
| *Procavia transvaalensis* | COB 102 | Cooper’s B | 45 | *Procavia transvaalensis* |  |
| *Procavia transvaalensis* | KA 1-1190 | Kromdraai A | 45 | *Procavia transvaalensis* |  |
| *Procavia transvaalensis* | KA 23 | Kromdraai A | 45 | *Procavia transvaalensis* |  |
| *Procavia transvaalensis* | KA 48 | Kromdraai A | 45 | *Procavia transvaalensis* |  |
|  |  |  |  |  |  |
| Order Lagomorpha^p^ |  |  |  |  |  |
| Family Leporidae |  |  |  |  |  |
| *Lepus bolti* | TM 1546 | - | - | *Lepus sp.* |  |
| *Pronolagus intermedius* | TM 1509 | - | - | *Pronolagus* sp. |  |
|  |  |  |  |  |  |
| Order Perissodactyla |  |  |  |  |  |
| Family Equidae |  |  |  |  |  |
| *Equus capensis* | SK 3983 | Swartkrans Member 1 | 46 | *Equus capensis* |  |
|  |  |  |  |  |  |
| Order Proboscidea |  |  |  |  |  |
| Family Elephantidae |  |  |  |  |  |
| *Elephas ekorensis* | STS 1863 | Sterkfontein Member 4 | 47 | *Elephas recki* | 48-49 |

^a^ Several alternative spellings of the ‘site’ Skurweberg have been used in the literature (e.g., Schurveberg 10; Skurveberg 1). Here we follow the spelling currently employed in official documents and maps distributed by South African government agencies.

^b^ These polygonal meshes were generated from microCT datasets and not from the Artec Spider surface scanner. MicoCT isometric voxel sizes (mm): SK 599: 0.0904; CO 100: 0.0879; STS 394a: 0.071; STS 394b: 0.038 (left hemimandible)/0.045 (right hemimandible).

^c^ MicroCT isometric voxel size: 0.0763mm.

^d^ These Sterkfontein primate specimens are physically marked with two numbers. The numbers with the STS prefix are those used within the literature. The other numbers (indicated here in parentheses) lack a prefix but may represent those assigned by Broom during their original cataloging and curation. We include both numbers here as they are both visible on the specimens (and full-color surface scans of these specimens).

^e^ This specimen may be ultimately reattributed to *Cercopithecoides coronatus* 50.

^f^ Additional *Cercopithecoides williamsi* specimens that have not been surface scanned but have available microCT datasets (isometric voxel size in mm) are: KB 680/686 (0.0409), 5241 (0.685), 5277 (0.0188) (Kromdraai B 16); STS 252 (0.0519), 300 (0.0362), 344 (0.0452), 559a (0.0531) (Sterkfontein Member 4 1, 14); SK 579 (0.0255)(Swartkrans Member 2 1).

^g^ MicroCT isometric voxel size: 0.0292mm.

^h^ 10 states the specimen is derived from the ‘Bolt’s Workings’ on Sterkfontein, but has been attributed to the Sterkfontein Type Site 25 and ‘Sterkfontein’ 26.

^i^ As with the TM 1579 specimen, these two *M. transvaalensis* specimens are described by 27, and subsequently by 28 as derived from ‘Bolt’s workings’ on Sterkfontein. A more precise provenience of these specimens (e.g. clearly to a Member of Sterkfontein or named deposit of the Bolt’s Farm complex) is impossible.

^j^ While 20 lists the origin for the specimen as ‘Sterkfontein’, 30 list the specimen as derived from Swartkrans; it is unclear if this latter attribution reflects a reinterpretation of the site origin or a typographic error.

^k^ 20 expresses uncertainty over the provenience of the specimen, potentially derived from Sterkfontein or ‘Bolt’s workings’ (favoring the latter).

^l^ Alternatively referable to *Crocuta ultra* per 35.

^m^ This specimen *may* represent the *H. striata* mandible described by 27 and 51, but missing in the collections during the work by 38. The m1 preserves a large metaconid, well-defined talonid with distinct hypoconid and entoconid and is consistent with the KB 295 *H. hyaena* specimen described by 22.

^n^ Alternatively attributable to *Phacochoerus modestus* per 41.

^o^ This specimen may be derived from Pit 10 (Grey Bird Pit; UCMP V-67263) of the Bolt’s Farm site complex.

^p^ No reference to either of these lagomorph specimens, or species, could be found in the literature. It is possible that the *Lepus bolti* cranium is the Bolt’s Farm *Lepus* cf. *capensis* referred to by 52. Recently, 53 have advocated that South African fossil *Lepus* and *Pronolagus* be retained at the genus level. Further investigation (both archival and morphological) may clarify the historical and taxonomic attribution of these two specimens.
