# Supplementary material for: Surface Model and Tomographic Archive of Fossil Primate and Other Mammal Holotype and Paratype Specimens of the Ditsong National Museum of Natural History, Pretoria, South Africa
Source: PLoS One. 2015 Oct 6;10(10):e0139800. doi: 10.1371/journal.pone.0139800 (PMC4595468; doi:10.1371/journal.pone.0139800)
Supplement: S2 Table — (DOCX) [file pone.0139800.s006.docx]

| **Current Taxonomic Allocation** | **Specimen Number** | **Surface Mesh (PLY) DOI** | **Surface Mesh (STL) DOI** | **Tomography (CT/microCT) DOI** |
| --- | --- | --- | --- | --- |
| Order Primates |  |  |  |  |
| Family Cercopithecidae |  |  |  |  |
| Subfamily Cercopithecinae |  |  |  |  |
| *Papio hamadryas robinsoni* | SB 2 | doi:10.17602/M2/M5463 | doi:10.17602/M2/M5581 |  |
| *Dinopithecus ingens* | SB 3 | doi:10.17602/M2/M5456 | doi:10.17602/M2/M5575 |  |
| ***Dinopithecus ingens*** | SB 7 | doi:10.17602/M2/M5415 | doi:10.17602/M2/M5539 |  |
| *Dinopithecus ingens* | SK 554 | doi:10.17602/M2/M5599 | doi:10.17602/M2/M5576 |  |
| *Dinopithecus ingens* | SK 599 | doi:10.17602/M2/M5577 | doi:10.17602/M2/M5578 | doi:10.17602/M2/M5918 |
| *Dinopithecus ingens* | SK 604a | doi:10.17602/M2/M5462 | doi:10.17602/M2/M5580 |  |
| ***Papio angusticeps*** | KA 194 | doi:10.17602/M2/M5454 | doi:10.17602/M2/M5574 | doi:10.17602/M2/M5920 |
| *Papio angusticeps* | CO 100 | doi:10.17602/M2/M5453 | doi:10.17602/M2/M5573 | doi:10.17602/M2/M5938 |
| *Papio hamadryas robinsoni* | SK 555 | doi:10.17602/M2/M5465 | doi:10.17602/M2/M5583 |  |
| *Papio hamadryas ursinus* | TM 211 | doi:10.17602/M2/M5469 | doi:10.17602/M2/M5585 |  |
| ***Parapapio broomi*** | STS 564 (1238) | doi:10.17602/M2/M5507 | doi:10.17602/M2/M5593 |  |
| *Papio izodi* | STS 262 | doi:10.17602/M2/M5471 | doi:10.17602/M2/M5587 |  |
| *Parapapio broomi* | STS 562 (501) | doi:10.17602/M2/M5511 | doi:10.17602/M2/M5592 |  |
| ***Parapapio jonesi*** | STS 565 | doi:10.17602/M2/M5503 | doi:10.17602/M2/M5596 |  |
| ***Gorgopithecus major*** | KA 193 | doi:10.17602/M2/M5418 | doi:10.17602/M2/M5542 |  |
| *Parapapio broomi* | STS 563 (1460) | doi:10.17602/M2/M5605 | doi:10.17602/M2/M5594 |  |
| *Theropithecus oswaldi oswaldi* | SK 563 | doi:10.17602/M2/M5448 | doi:10.17602/M2/M5591 |  |
| *Theropithecus oswaldi oswaldi* | SK 402 | doi:10.17602/M2/M5452 | doi:10.17602/M2/M5624 |  |
| *Theropithecus oswaldi oswaldi* | SK 405 | doi:10.17602/M2/M5451 | doi:10.17602/M2/M5625 |  |
|  |  |  |  |  |
| Subfamily Colobinae^e^ |  |  |  |  |
| *Cercopithecoides williamsi* | KA 195 (KB 122) | doi:10.17602/M2/M5595 | doi:10.17602/M2/M5505 |  |
| *Cercopithecoides williamsi* | SK 551 | doi:10.17602/M2/M5297 | doi:10.17602/M2/M5506 | doi:10.17602/M2/M5919 |
| *Cercopithecoides williamsi* | STS 394a & b | doi:10.17602/M2/M5604  doi:10.17602/M2/M6577 | doi:10.17602/M2/M5508  doi:10.17602/M2/M6578 | doi:10.17602/M2/M5921  doi:10.17602/M2/M5922  doi:10.17602/M2/M5923 |
| *Cercopithecoides williamsi* | KB 5241 |  |  | doi:10.17602/M2/M5925 |
| *Cercopithecoides williamsi* | KB 5277 |  |  | doi:10.17602/M2/M5926 |
| *Cercopithecoides williamsi* | KB 680/686 |  |  | doi:10.17602/M2/M5927 |
| *Cercopithecoides williamsi* | SK 579 |  |  | doi:10.17602/M2/M5928 |
| *Cercopithecoides williamsi* | STS 300 |  |  | doi:10.17602/M2/M5929 |
| *Cercopithecoides williamsi* | STS 344 |  |  | doi:10.17602/M2/M5930 |
| *Cercopithecoides williamsi* | STS 559a |  |  | doi:10.17602/M2/M5924 |
| *Cercopithecoides williamsi* | STS 252 |  |  | doi:10.17602/M2/M5979 |
|  |  |  |  |  |
| Order Carnivora |  |  |  |  |
| Family Canidae |  |  |  |  |
| Tribe Canini |  |  |  |  |
| *Canis mesomelas* | STS 1582 | doi:10.17602/M2/M5291  doi:10.17602/M2/M5292 | doi:10.17602/M2/M5498 doi:10.17602/M2/M5499 |  |
| *Canis mesomelas* | TM 1583 | doi:10.17602/M2/M5293 | doi:10.17602/M2/M5501 |  |
| *Canis* sp. | KA 1288 | doi:10.17602/M2/M5294 | doi:10.17602/M2/M5502 |  |
| *Canis* sp. | KA 1556 | doi:10.17602/M2/M5295 | doi:10.17602/M2/M5504 |  |
| ***Canis brevirostris*** | STS 137 | doi:10.17602/M2/M5284  doi:10.17602/M2/M5285 | doi:10.17602/M2/M5490  doi:10.17602/M2/M5491 |  |
| *Canis mesomelas* | KA 73 | doi:10.17602/M2/M5288  doi:10.17602/M2/M5289  doi:10.17602/M2/M5290 | doi:10.17602/M2/M5494  doi:10.17602/M2/M5495  doi:10.17602/M2/M5496 |  |
| *Canis mesomelas* | KA 71 | doi:10.17602/M2/M5286  doi:10.17602/M2/M5287 | doi:10.17602/M2/M5492  doi:10.17602/M2/M5493 |  |
| ***Nyctereutes terblanchei*** | KA 1290 | doi:10.17602/M2/M5436 doi:10.17602/M2/M5437 | doi:10.17602/M2/M5563  doi:10.17602/M2/M5564 |  |
| Tribe Vulpini |  |  |  |  |
| ***Vulpes pattisoni*** | TM 1553 | doi:10.17602/M2/M5444 | doi:10.17602/M2/M5626 |  |
| ***Vulpes pulcher*** | KA 1289 | doi:10.17602/M2/M5441 | doi:10.17602/M2/M5627 |  |
|  |  |  |  |  |
| Family Felidae |  |  |  |  |
| Subfamily Felinae |  |  |  |  |
| ***Dinofelis barlowi*** | TM 1541 | doi:10.17602/M2/M5364 | doi:10.17602/M2/M5529 |  |
| ***Dinofelis barlowi*** | TM 1542 | doi:10.17602/M2/M5530 | doi:10.17602/M2/M5531 |  |
| *Dinofelis barlowi* | TM 1579 | doi:10.17602/M2/M5532 | doi:10.17602/M2/M5533 |  |
| *Dinofelis barlowi* | BF 55-22 | doi:10.17602/M2/M5362 | doi:10.17602/M2/M5527 |  |
| *Dinofelis barlowi* | BF 55-23 | doi:10.17602/M2/M5363 | doi:10.17602/M2/M5528 |  |
| ***Dinofelis piveteaui*** | KA 61 | doi:10.17602/M2/M5370  doi:10.17602/M2/M5371 | doi:10.17602/M2/M5534  doi:10.17602/M2/M5535 |  |
| *Dinofelis piveteaui* | KA 62 | doi:10.17602/M2/M5536 | doi:10.17602/M2/M5537 |  |
| *Dinofelis piveteaui* | KA 63 | doi:10.17602/M2/M5414 | doi:10.17602/M2/M5538 |  |
|  |  |  |  |  |
| Subfamily Machairodontinae |  |  |  |  |
| ***Machairodus transvaalensis*** | STS 130-299 | doi:10.17602/M2/M5422 | doi:10.17602/M2/M5546 |  |
| *Machairodus transvaalensis* | TM 1577 | doi:10.17602/M2/M5423 | doi:10.17602/M2/M5547 |  |
| ***Megantereon whitei*** | TM 856 | doi:10.17602/M2/M5428 | doi:10.17602/M2/M5552 |  |
| *Megantereon whitei* | KA 64 | doi:10.17602/M2/M5424  doi:10.17602/M2/M5425  doi:10.17602/M2/M5426 | doi:10.17602/M2/M5548  doi:10.17602/M2/M5549  doi:10.17602/M2/M5550 |  |
| *Megantereon whitei* | STS 1558 | doi:10.17602/M2/M5427 | doi:10.17602/M2/M5551 |  |
|  |  |  |  |  |
| Subfamily Pantherinae |  |  |  |  |
| *Panthera pardus* | KA 87 | doi:10.17602/M2/M5445 | doi:10.17602/M2/M5570 |  |
| *Panthera leo* | BF 1555 | doi:10.17602/M2/M5442  doi:10.17602/M2/M5443 | doi:10.17602/M2/M5568  doi:10.17602/M2/M5569 |  |
| *Panthera pardus* | SK 349 | doi:10.17602/M2/M5446  doi:10.17602/M2/M5447 | doi:10.17602/M2/M5571  doi:10.17602/M2/M5572 |  |
|  |  |  |  |  |
| Family Herpestidae |  |  |  |  |
| ***Atilax mesotes*** | KA 86 | doi:10.17602/M2/M5280  doi:10.17602/M2/M5281  doi:10.17602/M2/M5282 | doi:10.17602/M2/M5484  doi:10.17602/M2/M5485  doi:10.17602/M2/M5486 |  |
|  |  |  |  |  |
| Family Hyaenidae |  |  |  |  |
| *Crocuta crocuta* | KA 56 | doi:10.17602/M2/M5355 | doi:10.17602/M2/M5939 |  |
| *Crocuta crocuta* | KA 57a | doi:10.17602/M2/M5356 | doi:10.17602/M2/M5521 |  |
| *Crocuta crocuta* | KA 58 | doi:10.17602/M2/M5357  doi:10.17602/M2/M5358  doi:10.17602/M2/M5359  doi:10.17602/M2/M5360 | doi:10.17602/M2/M5522  doi:10.17602/M2/M5523  doi:10.17602/M2/M5524  doi:10.17602/M2/M5525 |  |
| *Crocuta crocuta* | CT 1 | doi:10.17602/M2/M5352 | doi:10.17602/M2/M5517 |  |
| *Crocuta crocuta* | CT 2 | doi:10.17602/M2/M5353 | doi:10.17602/M2/M5518 |  |
| *Crocuta crocuta* | CT 3 | doi:10.17602/M2/M5354 | doi:10.17602/M2/M5519 |  |
| *Crocuta crocuta* | SK 317 | doi:10.17602/M2/M5361 | doi:10.17602/M2/M5526 |  |
| ***Pachycrocuta brevirostris*** | KA 55 | doi:10.17602/M2/M5438  doi:10.17602/M2/M5439  doi:10.17602/M2/M5440 | doi:10.17602/M2/M5565  doi:10.17602/M2/M5566  doi:10.17602/M2/M5567 |  |
| *Parahyaena brunnea* | SK 326 | doi:10.17602/M2/M5477 | doi:10.17602/M2/M5590 |  |
| *Hyaena* cf. *hyaena* | KA 211Y | doi:10.17602/M2/M5420 | doi:10.17602/M2/M5544 |  |
| *Hyaena hyaena* | SK 314 | doi:10.17602/M2/M5419 | doi:10.17602/M2/M5543 |  |
| *Parahyaena brunnea* | SK 315 | doi:10.17602/M2/M5474 | doi:10.17602/M2/M5588 |  |
| *Parahyaena brunnea* | SK 316 | doi:10.17602/M2/M5476 | doi:10.17602/M2/M5589 |  |
| ***Chasmaporthetes nitidula*** | SK 301 | doi:10.17602/M2/M5299 | doi:10.17602/M2/M5509 |  |
| ***Lycyaenops silberbergi*** | STS 126 | doi:10.17602/M2/M5301 | doi:10.17602/M2/M5513 |  |
| *Lycyaenops silberbergi* | STS 130 | doi:10.17602/M2/M5602 | doi:10.17602/M2/M5516 |  |
| *Lycyaenops silberbergi* | SK 300 | doi:10.17602/M2/M5300 | doi:10.17602/M2/M5512 |  |
|  |  |  |  |  |
| Order Artiodactyla |  |  |  |  |
| Family Bovidae |  |  |  |  |
| Subfamily Caprinae |  |  |  |  |
| ***‘Bos’ makapaani*** | TM 315 | doi:10.17602/M2/M5283 | doi:10.17602/M2/M5487 |  |
|  |  |  |  |  |
| Family Suidae |  |  |  |  |
| *Metridiochoerus* sp. | STS 3074a & b | doi:10.17602/M2/M5434  doi:10.17602/M2/M5435 | doi:10.17602/M2/M5561  doi:10.17602/M2/M5562 |  |
| *Metridiochoerus andrewsi* | BF 1 | doi:10.17602/M2/M5429  doi:10.17602/M2/M5430  doi:10.17602/M2/M5431  doi:10.17602/M2/M5432  doi:10.17602/M2/M5975  doi:10.17602/M2/M5976  doi:10.17602/M2/M6579  doi:10.17602/M2/M6580 | doi:10.17602/M2/M5553  doi:10.17602/M2/M5554  doi:10.17602/M2/M5555  doi:10.17602/M2/M5556  doi:10.17602/M2/M5977  doi:10.17602/M2/M5978  doi:10.17602/M2/M6581  doi:10.17602/M2/M6582 | doi:10.17602/M2/M5582 |
| ***Phacochoerus antiquus*** | KA 89a & b | doi:10.17602/M2/M5488  doi:10.17602/M2/M5489 | doi:10.17602/M2/M5940  doi:10.17602/M2/M5941 | doi:10.17602/M2/M5584 |
| *Phacochoerus antiquus* | SK 382 | doi:10.17602/M2/M5482 | doi:10.17602/M2/M5606 |  |
| *Phacochoerus antiquus* | BF 3-335 | doi:10.17602/M2/M5497  doi:10.17602/M2/M5500 | doi:10.17602/M2/M5597  doi:10.17602/M2/M5598 |  |
| *Phacochoerus antiquus* | SK 4005 | doi:10.17602/M2/M5236 | doi:10.17602/M2/M5608 | doi:10.17602/M2/M5586 |
| Suidae indet. | SE 1069-1 | doi:10.17602/M2/M5455 | doi:10.17602/M2/M5623 |  |
|  |  |  |  |  |
| Order Eulipotyphla |  |  |  |  |
| Family Erinaceidae |  |  |  |  |
| ***Erinaceus (Atelerix) broomi*** | TM 1544 | doi:10.17602/M2/M5279 | doi:10.17602/M2/M5483 |  |
|  |  |  |  |  |
| Order Hyracoidea |  |  |  |  |
| Family Procaviidae |  |  |  |  |
| *Procavia transvaalensis* | ST 106  (TM 1462) | doi:10.17602/M2/M5480 | doi:10.17602/M2/M5610 |  |
| *Procavia antiqua* | ST 105 | doi:10.17602/M2/M5481 | doi:10.17602/M2/M5609 |  |
| *Procavia transvaalensis* | CO 1 | doi:10.17602/M2/M5479 | doi:10.17602/M2/M5611 |  |
| *Procavia transvaalensis* | CO 10 | doi:10.17602/M2/M5478 | doi:10.17602/M2/M5612 |  |
| *Procavia transvaalensis* | CO 11 | doi:10.17602/M2/M5475 | doi:10.17602/M2/M5613 |  |
| *Procavia transvaalensis* | CO 12a | doi:10.17602/M2/M5473 | doi:10.17602/M2/M5614 |  |
| *Procavia transvaalensis* | COB 102 | doi:10.17602/M2/M5472 | doi:10.17602/M2/M5615 |  |
| *Procavia transvaalensis* | KA 1-1190 | doi:10.17602/M2/M5459 | doi:10.17602/M2/M5621 |  |
| *Procavia transvaalensis* | KA 23 | doi:10.17602/M2/M5466 | doi:10.17602/M2/M5619 |  |
| *Procavia transvaalensis* | KA 48 | doi:10.17602/M2/M5464 | doi:10.17602/M2/M5620 |  |
|  |  |  |  |  |
| Order Lagomorpha |  |  |  |  |
| Family Leporidae |  |  |  |  |
| *Lepus sp.* | TM 1546 | doi:10.17602/M2/M5421 | doi:10.17602/M2/M5545 |  |
| *Pronolagus* sp. | TM 1509 | doi:10.17602/M2/M5457 | doi:10.17602/M2/M5622 |  |
|  |  |  |  |  |
| Order Perissodactyla |  |  |  |  |
| Family Equidae |  |  |  |  |
| *Equus capensis* | SK 3983 | doi:10.17602/M2/M5417 | doi:10.17602/M2/M5541 |  |
|  |  |  |  |  |
| Order Proboscidea |  |  |  |  |
| Family Elephantidae |  |  |  |  |
| *Elaphas recki* | STS 1863 | doi:10.17602/M2/M5416 | doi:10.17602/M2/M5540 |  |
